# On *Hydrangea peruviana*, an endangered species from Ecuador, and *Hydrangea oerstedii*, very common in Costa Rica and Panama, and seven threatened Central and South American Hydrangeas, which have been confounded with these

**DOI:** 10.3897/phytokeys.171.56351

**Published:** 2021-01-26

**Authors:** Marie-Stéphanie Samain, Carolina Granados Mendoza, Esteban Manuel Martínez Salas

**Affiliations:** 1 Red de Diversidad Biológica del Occidente Mexicano, Instituto de Ecología, A.C., 61600 Pátzcuaro, Michoacán, Mexico; 2 Departamento de Botánica, Instituto de Biología, Universidad Nacional Autónoma de México, 04510, Mexico City, Mexico; 3 Herbario Nacional de México, Departamento de Botánica, Instituto de Biología, Universidad Nacional Autónoma de México, 04510, Mexico City, Mexico

**Keywords:** Conservation, Ecuador, functional dioecism, hortensia, lianas, Neotropics, Peru, taxonomy

## Abstract

Hydrangea section Cornidia, currently consisting of 19 accepted taxa, occurs from Mexico to Chile and Argentina, with one species in southeast Asia. Its representatives are root-climbing lianas which may grow up to 60 m high in the tree canopy of temperate to (sub)tropical forests. Our extensive field work throughout its distribution area, study of herbarium specimens and ongoing molecular studies have resulted in the discovery of species new to science, as well as new insights into the circumscription of many taxa. We here present amended descriptions for seven *Hydrangea* species of Central and South America and discuss the taxonomical situation of two Colombian Hydrangeas, including an identification key, illustrations, and distribution maps. Field work was carried out in Costa Rica, Panama, Ecuador and Peru, including exploration in areas where the genus had not been collected before. These specimens and observations were complemented with the study of specimens of 41 herbaria of North, Central and South America, as well as Europe. Detailed morphological studies of all species were carried out, based on living plants in their natural habitat, as well as on dried specimens from our own collections and all available herbarium material. Type material was studied in detail for all species concerned. Based on an extensive number of morphological characters, combined with distribution patterns, phenological differences and ecological preferences, including molecular data in most cases, *Hydrangea peruviana* and *H. oerstedii* are clearly distinct taxa, as well as the other seven species mentioned here, which had been synonymized with either of these two species. The present study results in the recognition of 26 species in section Cornidia and exemplifies the urgent need for profound taxonomic studies in plants, as in many families we do not dispose of well-circumscribed units for conservation to mitigate the already occurring unprecedented loss of biodiversity.

## Abstract (es)

*Hydrangea* sección *Cornidia*, que actualmente consiste en 19 taxones aceptados, se distribuye desde México hasta Chile y Argentina, con una especie en el sureste asiático. Sus representantes son lianas trepadoras que pueden crecer hasta 60 m de altura en la copa de los árboles de los bosques templados a (sub)tropicales. Nuestro extenso trabajo de campo en toda su área de distribución, la revisión de especímenes de herbario y los estudios moleculares en curso han dado como resultado el descubrimiento de especies nuevas para la ciencia, así como conocimiento nuevo sobre la circunscripción de muchos taxones. Aquí presentamos descripciones enmendadas para siete especies de *Hydrangea* de Centro y Suramérica y discutimos la situación taxonómica de dos Hydrangeas colombianas, incluyendo una clave de identificación, ilustraciones y mapas de distribución. El trabajo de campo se llevó a cabo en Costa Rica, Panamá, Ecuador y Perú, incluída la exploración en áreas donde el género no había sido recolectado antes. Estos especímenes y observaciones se complementaron con el estudio de especímenes de 41 herbarios de Norte, Centro y Suramérica, así como de Europa. Se llevaron a cabo estudios morfológicos detallados de todas las especies, basados ​​en plantas vivas en su hábitat natural, así como en muestras secas de nuestras propias colecciones y todo el material de herbario disponible. El material tipo se estudió en detalle para todas las especies en cuestión. En base a una gran cantidad de caracteres morfológicos, combinados con patrones de distribución, diferencias fenológicas y preferencias ecológicas, incluyendo datos moleculares en la mayoría de los casos, *Hydrangea peruviana* y *H. oerstedii* son taxones claramente distintos, así como las otras siete especies mencionadas aquí, que habían sido sinonimizadas con cualquiera de estas dos especies. El presente estudio resulta en el reconocimiento de 26 especies en la sección *Cornidia* y ejemplifica la necesidad urgente de estudios taxonómicos profundos en plantas, ya que en muchas familias no disponemos de unidades de conservación bien circunscritas para mitigar la pérdida de biodiversidad sin precedentes.

## Introduction

The relatively poorly known Hydrangea L. section Cornidia Ruiz & Pav. consists of 19 currently accepted taxa (18 species and one variety) and a yet undefined number of species new to science and taxa that have been erroneously synonymized. The representatives of this section occur from northern Mexico to southern Chile and Argentina with one species, *Hydrangea integrifolia* Hayata, in southeastern Asia (Samain et al. 2014; Samain and Martínez Salas 2015; Samain et al. 2019). All taxa are evergreen root climbers growing up to 60 m high in the canopy of mostly primary forests, or rarely on boulders and rock walls (Granados Mendoza et al. 2014), generally functionally dioecious, rarely monoecious, with coriaceous leaves and hortensia-like whitish-, greenish-, yellowish-, reddish- or purplish-tinged inflorescences, with or without enlarged marginal flowers.

Hydrangea section Cornidia (hereafter shortened as *Cornidia*) is monophyletic, including the single Asian species (Samain et al. 2010; Granados Mendoza et al. 2013a, b, 2015; De Smet et al. 2015), and is sister to the section Calyptranthe Maxim., consisting of Asian climbing species (De Smet et al. 2015). Both clades together are sister to the section Asperae (Rehder) Y.De Smet & Samain, encompassing Asian shrubby species (De Smet et al. 2015). As already noted by Samain et al. (2019), we do not follow the nomenclatural changes proposed by Ohba and Akiyama (2016), who suggest recognition of *Cornidia* as a segregate genus within tribe Hydrangeeae, together with most of the other sections published by De Smet et al. (2015), as they do not take into account the evolutionary context in which the new classification of the tribe Hydrangeeae was presented.

The monography of the genus *Hydrangeas.s.* by McClintock (1957), which is entirely based on herbarium specimens, is the only available recent complete revision of this section and includes 12 accepted species, classified in two subsections, *Monosegia* Briq. and *Polysegia* Briq. As mentioned by Samain et al. (2014), the revision by McClintock (1957) oversimplifies the high morphological variation within *Cornidia* in the Neotropics, due to inappropriate synonymization of taxa (since the description of *Cornidia* as a genus by Ruiz and Pavón (1794), more than 40 taxa have been described within this group), and to the fact that many diagnostic morphological characters were not taken into account (Samain et al. 2010, 2019). Following the revision of the genus *Hydrangea* by Freire-Fierro (2004) for the Flora of Ecuador, who considered *Hydrangea oerstedii* Briq. as a variety of *Hydrangea peruviana* Moric. ex Ser., only eleven species were recognized before the start of our revision of the genus (Samain et al. 2014).

We have carried out extensive field work throughout the distribution area of *Cornidia* since 2009, and as a consequence, we realized that its representatives are much more common than previously known (albeit most of them are severely threatened, mainly because of habitat destruction) and that its incredible morphological variation definitely did not fit into the then eleven accepted species. However, contrary to Mexico where we have recently described seven new *Hydrangea* species and registered one new record of a species until then only known from Guatemala (Samain et al. 2014, 2019), an important portion of the morpho-species in Central and South America do coincide with earlier described species which are currently considered to be synonyms.

Apart from the considerable confusion over names, an additional challenge in this group is the functional dioecism, which we have observed in most individuals during our extensive field work throughout the Neotropics, and which is reflected by a notoriously different size and shape of flower receptacle, anthers and pistils between functionally female and male flowers (Samain et al. 2014, 2019; Samain and Martínez Salas 2015). Interestingly, some species do not show this functional dioecism at all (e.g., *H. seemannii* L.Riley which has bisexual flowers, Samain et al. 2019), whereas some others may show functionally dioecious or perfect flowers depending on the individual.

The above-mentioned issues emphasize the need for a complete and urgent revision of *Cornidia*, especially in the light of conservation of these species. Their pristine habitat with very specific conditions (near water, often flat topography near the plants, efficient drainage) makes them not only promising bio-indicators, but also poses an additional threat as these habitats are being destroyed because they are highly appreciated by local people for agriculture (Samain et al. 2019). Our recent publications on *Cornidia* focused on the Mexican species were based on 186 herbarium collections, of which 50% were collected by ourselves (Samain et al. 2014, 2019).

The present work aims at resolving the complex of nine species from Central and South American species that had been formally synonymized under *Hydrangea peruviana* (*H. schlimii* Briq., *H. caucana* Engl., *H. durifolia* Briq., *H. goudotii* Briq., *H. oerstedii* Briq., *H. panamensis* Standl., *H. peruviana* Moric. ex. Ser., *H. trianae* Briq. and *H. weberbaueri* Engl.). The main objective of the present study is to show that we are dealing with nine different species, primarily based on morphological characters, and strengthened by preliminary molecular data. The identification key which is presented here thus has no other aim than showing that these species can be distinguished with relative ease based on morphological characters that are straightforward to observe. Based on meticulous observations in the field and of herbarium specimens, we here present amended descriptions for seven *Hydrangea* species of Central and South America and discuss the taxonomic situation of two more species of this group, including an identification key of species as recognized here, illustrations, and distribution maps, as well as information about their diversity, their global conservation status, and their affinities with other *Cornidia* species.

## Material and methods

Field work focused on *Hydrangea* has been carried out by the authors during dry and rainy seasons in Costa Rica (2012, 2013), Ecuador (2012), Panama (2019) and Peru (2011, 2012, 2013), coinciding with the flowering and fruiting seasons, based on herbarium material of most of the species included. Exploring field work was not only carried out in the areas where previous collections had been made, but also in zones where Hydrangeas had not yet been recorded and where we suspected they would be present, based on our knowledge of their habitat preferences. Branches with inflorescences, flowers and fruits of all stages were collected and preserved. Moreover, in several individuals where architectural traits seemed to be important, whole branches were collected, of course without affecting the viability of the individual plants, cut and subsequently numbered in order to maintain the architecture available for further study. All specimens were deposited in local herbaria in the respective countries where we collected (CR (including INB), HOXA, PMA, QCNE and USM) with duplicates in the herbaria of the Instituto de Ecología, A.C. (IEB) in Pátzcuaro, Michoacán, Mexico, the Ghent University (GENT), in Ghent, Belgium and the National Herbarium of Mexico (MEXU) in Mexico City, Mexico.

Our field observations were complemented with a detailed study of relevant herbarium specimens of 41 herbaria in Europe, North, Central and South America (A, AAU, AMAZ, B, BM, BR, C, CAS, COL, CR, DUKE, E, F, G, GENT, GB, GH, HOXA, HUA, IEB, INB (now CR), K, LOJA, MA, MEXU, MICH, MO, MOL, MPU, NY, P, PMA, QCA, QCNE, QPLS, UC, UCH, UPS, US, USM, and WU; acronyms according to Thiers, continuously updated), most of them loaned, few (mainly type specimens) in high resolution on JSTOR Global Plants (https://plants.jstor.org/). A few herbaria, where loans were not allowed or logistically not possible, were visited in person to study the material in detail and take high quality photographs for later reference. A total number of 407 collections are included in this study, of which 75 have been collected by the authors of this paper. Both numbers may seem relatively low for a taxonomic revision; however, given that most of the species are rare to very rare on the one hand, and that hiking to primary vegetation followed by climbing of the host tree is required to reach inflorescences in most individuals on the other hand, these are highly representative. Indeed, as can be seen from the list of herbaria, most herbaria in the study area, as well as foreign herbaria with important collections (both recent and historical), have been consulted. Countries in the Specimens Examined section are listed alphabetically. For each specimen with reproductive structures studied, we mention whether it is functionally female (♀) or functionally male (♂), as well as which structures are present. Measurements were taken from dry herbarium specimens and three or more measurements per structure were taken when possible. Floral organ measurements were based on dry flowers. Colors were based on photographs of living plants and notes on herbarium labels. With respect to the leaf vein morphology, we use the standard terms we have used since our first treatment of this genus (Samain et al. 2014) in order to provide consistency: midvein for the central vein of the leaf, primary veins for the first order veins, secondary veins for the second order veins, and tertiary veins for the third order veins. The leaf characters in the identification key refer to the large, mature leaves on the stems and not to the leaves on the inflorescence axes.

Red List categories were obtained according to the IUCN Red List criteria (IUCN 2012). All known localities, including those of herbarium specimens without coordinates, were geo-referenced using Google Earth (2020). Extent of Occurrence (EOO) and Area of Occupancy (AOO) of all species were calculated with GeoCAT (Bachmann et al. 2011). Distribution maps were obtained with the same set of coordinates using ArcGIS v.10 software by Esri (www.esri.com).

## Results

### Taxonomy

The present treatment includes the *Cornidia* species *Hydrangea oerstedii* and *H. peruviana*, plus seven other species which had been erroneously synonymized with the former two. Amended morphological descriptions for seven of these and an identification key to the taxa treated here are provided. We do not repeat here the morphological description of the section as this has been published in an Open Access paper by Samain et al. (2019). All species included here are characterized by lateral and umbellate inflorescences with reddish to purplish marginal and reduced flowers, except for *H. panamensis*, which has been reported with pink, yellow or white marginal flowers; hence, the earlier confusion of all these species, although in fact they are easily distinguishable. There are other taxa with similar colors in this section, but these have never been synonymized with the two abovementioned species, although South American herbarium specimens of these taxa may also be identified as *H. peruviana*. These taxa will be treated in an upcoming manuscript with species surrounding *Hydrangea preslii* Briq.

It should be mentioned that our ongoing molecular studies in the *Cornidia* clade show that most of the species studied here are even not closely related, with the exception of *Hydrangea panamensis* and *H. peruviana* on the one hand, and *H. goudotii* and *H. trianae* on the other hand (Granados Mendoza 2013; Granados Mendoza et al. unpublished data). Hence, we are not treating a monophyletic group of species here. Nevertheless, given that the focus of the present paper is morphological-taxonomical, and as all nine species since the 1950s are continuously considered as *H. peruviana*/*H. oerstedii* on herbarium specimens, in revisions and treatments (McClintock 1957; Freire-Fierro 2004; Christenhusz 2009), as well as in local floristic lists and catalogues such as those of Costa Rica (Morales Quirós 2007), Ecuador (Jørgensen and León-Yánez 1999), Panama (Correa et al. 2004), and Peru (Brako and Zarucchi 1993), we consider it appropriate to show in a single treatment that we are dealing with clearly distinct taxa which deserve recognition at species level based on an extensive range of morphological characters.

Of the nine taxa treated below, we have observed six in the field throughout their distribution area. *Hydrangea caucana* Engl., *H. durifolia* Briq. and *H. schlimii* Briq. have not been collected recently and are known with certainty from nine herbarium specimens, the type collection and a putative additional herbarium specimen, or the type collection only, respectively. The three of them are endemic to Colombia, where we have not yet been able to collect Hydrangeas due to collection permit and export regulations.

### Key to the species of Hydrangea section Cornidia which have been confused with *Hydrangea oerstedii* and *Hydrangea peruviana*, including these two species

(Note: as mentioned above, this is a partial key that shows that these species are easy to distinguish. It cannot be used to identify all red-flowered Hydrangeas of Central and South America)

**Table:** 

1	Inflorescences mainly consisting of flowers with enlarged sepals; currently only known from the type locality in Colombia	**7. *H. schlimii* Briq.**
–	Inflorescences with only the marginal flowers with enlarged sepals, these flowers placed terminally on cymes or racemes, one per partial inflorescence, or rarely absent; occurring in Costa Rica, Panama, Colombia, Ecuador or Peru	**2**
2	Leaves up to 13 cm long and 6 cm wide	**3**
–	Leaves longer than 13 cm and wider than 6 cm	**6**
3	Leaf apex rounded with a very small acumen; acarodomatia on the abaxial side 0–2 per leaf; Costa Rica and Panama	**5. *H. panamensis* Standl.**
–	Leaf apex acute to acuminate, rarely mucronate; acarodomatia more numerous; occurring in Central and South America	**4**
4	Lamina very slightly spoon-shaped, elliptic to slightly obovate; leaf margin serrate to slightly dentate; endemic to Ecuador	**6. *H. peruviana* Moric. ex Ser.**
–	Lamina flat, ovate to lanceolate-elliptic; leaf margin glandular dentate; Costa Rica, Panama and Colombia	**5**
5	Free-growing branches and inflorescence axes pubescent with persistent reddish brown stellate hairs; endemic to Colombia	**1. *H. caucana* Engl.**
–	Free-growing branches glabrous, and inflorescence axes pubescent with caducous, appressed, white stellate hairs; Costa Rica and Panama	**4. *H. oerstedii* Briq.**
6	Leaves with primary veins and secondary veins parallel to each other and forming a distinct regular pattern with all veins arching towards the apex; Colombia, Ecuador and Peru	**9. *H. weberbaueri* Engl.**
–	Leaves with a pinnate leaf vein pattern	**7**
7	Free-growing branches and inflorescence axes glabrous	**8**
–	Free-growing branches with appressed white caducous stellate pubescence	**9**
8	Abaxial leaf side with a granulate texture due to remaining basal stalks of stellate pubescence; inflorescence axis terete; inflorescence 5–9.5 cm wide; cymes compact; currently known from Colombia only	**2. *H. durifolia* Briq.**
–	Abaxial leaf side pubescent with caducous, appressed, white, stellate hairs; inflorescence axis angled; inflorescence 6–28 cm wide; cymes lax; widely distributed in Costa Rica and Panama	**4. *H. oerstedii* Briq.**
9	Leaves markedly coriaceous; secondary and tertiary veins on the abaxial leaf side forming a reticulate network, connecting the primary veins; apical portion of flowering branch including leaves and buds densely pubescent with erect hairs; Colombia, Ecuador and Peru	**8. *H. trianae* Briq.**
–	Leaves papyraceous; secondary and tertiary veins on the abaxial leaf side not forming a reticulate network; apical portion of flowering branch including leaves and buds scarcely to densely pubescent with appressed hairs; Colombia and Ecuador	**9. *H. goudotii* Briq.**

#### 1. Hydrangea caucana

Taxon classificationPlantaeCornaleHydrangeaceae

Engl., Nat. Pflanzenfam. (ed. 2) 18a: 206. 1930.

EA90E0D3-706C-538C-83B3-BC56B4669C02

Figures 1 , 2

##### Type.

Colombia. Cauca: Caldas, Las Pavas Cimarronas, 1200–1600 m, ♀, flowers, *F.C. Lehmann 5106* (lectotype, designated by McClintock 1957, pg. 240 [as “isotype”], second-step designated here: K! [K000486133], isolectotypes: F! [F0066623F], K! [K000486134].

##### Description.

**Figure 1.**
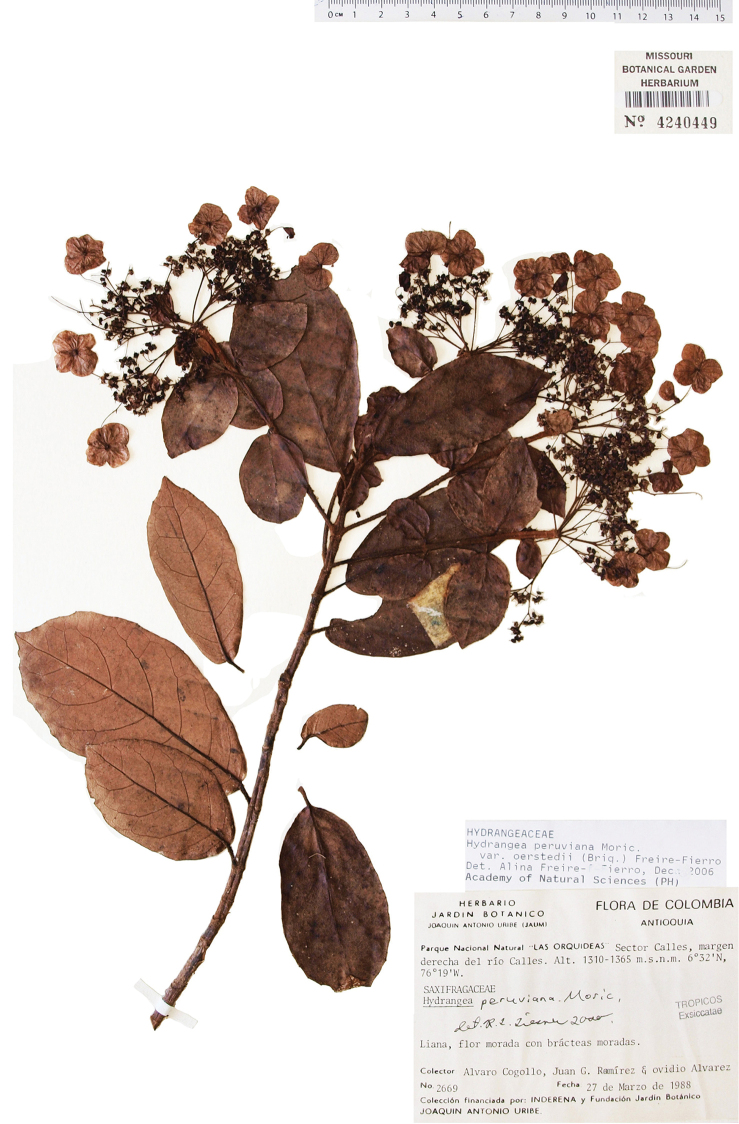
*Hydrangea caucana*. Branch with infructescences with mature fruits and enlarged marginal flowers. Image of specimen *A. Cogollo et al. 2669* (MO).

Root-climbing liana of probably not more than 15–20 m high; functionally dioecious; ***free-growing branches*** slightly quadrangular, densely pubescent with reddish brown stellate hairs; ***leaves*** decussate, petiole sulcate adaxially, terete abaxially, color reddish brown, densely pubescent with caducous, reddish-brown, stellate hairs, 0.6–1.5 cm long, leaving a semicircular scar on the branch when leaves shed; lamina flat, ovate to lanceolate-elliptic, 6–12 cm long, 2.6–5.5 cm broad, base rounded to decurrent, sometimes asymmetric, apex acuminate, leaf margin glandular dentate, teeth generally small, larger in only a few leaves, venation brochidodromous, veins 5–7 pairs, adaxial leaf side with marked midvein, primary and secondary veins lightly marked, primary veins join to form submarginal vein, sparsely pubescent with appressed, white stellate hairs, in young leaves more dense and reddish hairs, abaxially with protruding veins, primary veins sometimes alternating protruding and marked in the same leaf, dark brown green, densely pubescent with appressed stellate reddish hairs near the midvein, rest of the lamina more sparsely pubescent, acarodomatia present, numerous, consisting of a simple cavity, but often not very conspicuous as they lay hidden under the midvein pubescence, in axils of midvein and primary veins; ***inflorescence axis*** densely pubescent with persistent reddish brown, stellate hairs, more dense towards the apex, 8–21 cm long, many-ribbed, with up to 3 opposite or decussate leaf pairs and up to 3 scars of possibly kataphyll pairs below the inflorescence, deciduous, petiole 5–15 mm long, lamina 6–12.5 cm long, 2.5–5.5 cm broad, kataphylls not seen, ***apex of the floral axis*** woody, basally quadrangular, apically triangular, elongated bract scars visible, 3–4 mm broad, 1.5–2 mm high in functionally female plants, 3.5–5 mm broad, 2–2.5 mm high in functionally male plants, ***inflorescence bracts*** cucullate densely pubescent, hairs reddish-brown, stellate, increasing in size, lowermost bract 1.5 cm large, 1.2 cm broad, other bracts not visible, ***inflorescences*** lateral (Fig. 1), decussate, 1–3 pairs of inflorescences per flowering branch, flowering branch only continues growing vegetatively rapidly during inflorescence development, with up to 6 leaf and kataphyll pairs above the inflorescences, inflorescence axes with basal lignified parts of inflorescences of previous years not seen, kataphylls at the base of the inflorescence present, orbicular, inflorescence umbellate, buds not seen, in flowering stage 4–12 cm diameter, 2.5–7 cm high, with 6–9 main axes in functionally male plants, 6–8 main axes in functionally female plants, partial inflorescences cymes, secondary and tertiary inflorescence axes densely pubescent with reddish, stellate hairs, pubescence gradually decreasing towards flower insertion; ***enlarged marginal flowers*** always present, terminally placed in a cyme, sepals 4, separate, with marked veins, pistils 2, fertile or reduced, some flowers with mature fruit, in these cases only 1 sepal visible, 1.5–2.3 cm diameter, pedicel 1–2 cm long, reddish-purple; ***flower pedicel of reduced flowers***, 0.2–1.5 mm long in functionally male flowers, 0.5–2(–3) mm long in functionally female flowers, ***receptacle*** triangular in functionally male flowers, semiglobose in functionally female flowers, ***ovary*** inferior, ***calyx lobes*** 4, triangular in male flowers, nearly reduced to zero in female flowers, enlarging during fruit maturation, reduced, ***petals*** 4, bright red to purple, cucullate, 1.2 mm long; ***functionally male flowers***: hypanthium 1–1.2 mm broad, 0.8–1 mm high, stamens 8, well-developed, pink, filaments 0.5–1.5 mm long, anthers 0.5 mm long, 0.2 mm broad, pistils 2, reduced, 0.2 mm long, stigmas not penicellate; ***functionally female flowers***: hypanthium 1 mm diameter, 0.5 mm high, stamens 8, reduced, filaments and anthers together 0.2 mm long, pistils 2, 0.5–0.8 mm long, enlarging up to 2 mm during fruit maturation, stigmas slightly apically clavate and shortly penicellate; ***fruit*** a semiglobose capsule, apically with a thickened border, dark reddish brown, 0.8–1.2 mm high, 1.5 mm broad above, 1.5 mm diameter, opening between the two pistils to release seeds, seeds not seen.

##### Distribution.

**Figure 2.**
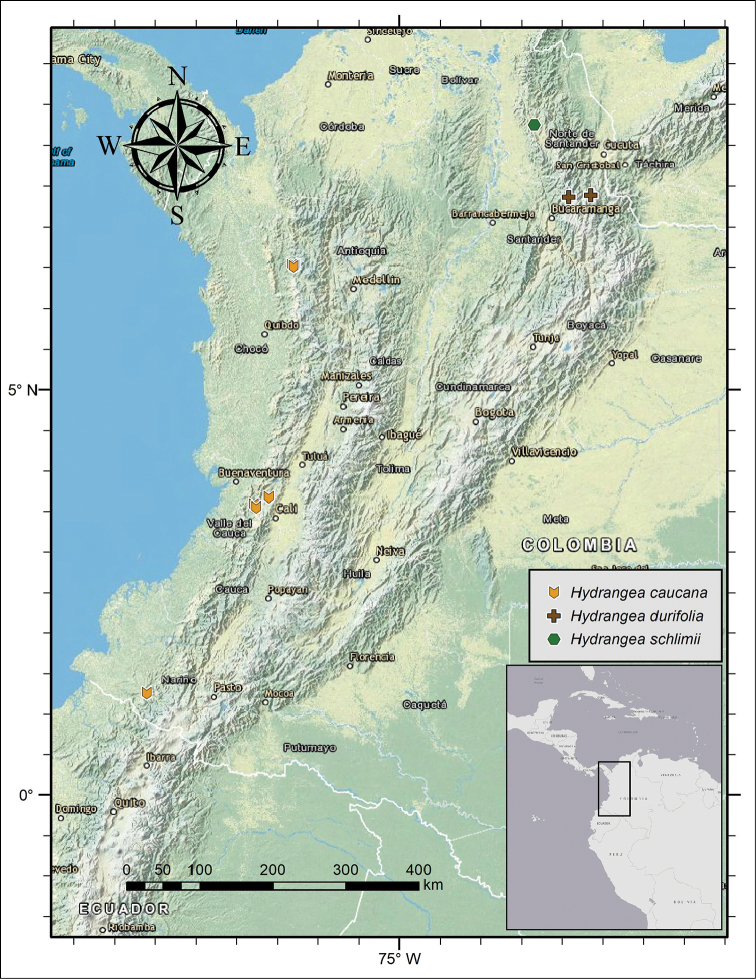
Distribution of *Hydrangea caucana*, *Hydrangea durifolia* and *Hydrangea schlimii*.

This species is endemic to Colombia and currently only known from the departments of Antioquia, Nariño and Valle de Cauca (Fig. 2).

##### Habitat.

*Hydrangea caucana* is known from mountain cloud forest at elevations between 750–1365 m.

##### Phenology.

*Hydrangea caucana* has been collected with flowers and fruits between November and March.

##### Notes.

McClintock (1957) cited the K material as lectotype albeit without designating a specific sheet, which was copied by Freire-Fierro (2004). The holotype in B was destroyed; a photo of this specimen is available in F. We here select the better of the two sheets at K in a second-step lectotypification.

*Hydrangea caucana* should not be considered a synonym of *H. peruviana* and can be distinguished from the latter species by the flat, ovate to lanceolate-elliptic leaves with a glandular dentate margin. In contrast, *H. peruviana* is characterized by very slightly spoon-shaped, elliptic to slightly obovate leaves with a serrate to slightly dentate margin. Moreover, *H. caucana* is currently only known from Colombia, whereas *H. peruviana* is restricted to Ecuador.

We have not observed this species in the field, and herbarium labels of the known specimens of *H. caucana* do not record the size of the plants.

The phylogenetic relationships of *Hydrangea caucana* are yet unknown as there was no fresh material available for our molecular study (Granados Mendoza et al. unpublished results).

##### Preliminary conservation status.

Based on the available herbarium collections, this species is Endangered according to the IUCN categories and criteria (IUCN 2012), with an AAO of 32 km^2^, fewer than five locations and an extensive reduction in both EOO and AOO due to habitat destruction and fragmentation. The most recent collection of this species we have seen is from 1992, so further exploration is needed to know the current distribution and conservation status of this species.

##### Additional specimens examined.

**Colombia. Antioquia**: Urrao, Aguadas, 1300 m, 8 Dec 1992, sterile, *Pipoly et al. 16776* (MO); Frontino, Vereda Venados, Parque Nacional Natural Las Orquídeas, margen izq. Río Venados (Garrucha y Alto Bonito), 6°32'N, 76°19'W, 800–850 m, 30 Jan 1995, ♂, flowers, *Pipoly et al. 18117* (MO, NY); same data as preceding, Parque Nacional Natural las Orquídeas, sector Calles, margen derecha del Río Calles, 1310–1365 m, ♀, fruits, 27 Mar 1988, *Cogollo et al. 2669* (COL, MO); same data as preceding, 1360 m, ♂, flowers, 19 Feb 1989, *Cogollo et al. 4091* (MO); **Nariño**: Mpio. de Ricaurte, Resguardo Indígena Nulpe Medio, camino a la quebrada La Conga, 1°6'N, 78°13'W, 750 m, 8 Jan 1996, ♂, flowers, *González & Ramírez 1636* (QCA); municipio Barbacoas, corregimiento Altaquer, Vías Las Vegas, al borde del río Veza, 870 m, flower buds, Mar 1995, *Fernández et al. 12459* (COL); **Valle de Cauca**: Cordillera Occidental, vertiente occidental, Hoya del Río Digua, lado derecho, La Elsa, 1000–1200 m, 9 Nov 1943, ♀, flowers, fruits, *Cuatrecasas 15326* (F, P, US); Cordillera Occidental, vertiente occidental, Hoya del Río Digua, lado derecho, entre Queremal y La Elsa, 1200–1160 m, ♀, 27, 29 Mar 1947, ♀, flowers, *Cuatrecasas 23994* (F, US).

#### 2. Hydrangea durifolia

Taxon classificationPlantaeCornaleHydrangeaceae

Briq., Annuaire Conserv. Jard. Bot. Genève 20: 406. 1919.

1ADBD397-AD9F-5950-B4F9-2D317F61A785

Figures 2 , 3 , 4

##### Type.

Colombia. Norte de Santander: Pamplona, ♂, flowers, *N. Funck & L.J. Schlim 1393* (lectotype, designated by McClintock 1957, pg. 238 [as “holotype”], second-step designated here: G! [G00223926]; isolectotypes: BR (photo in COL!), G! [G00301423], MPU! [MPU22860]).

##### Description.

The most complete description to date can be found in the treatment by Briquet (1919), pages 406–407.

##### Distribution.

*Hydrangea durifolia* is known from the type and an additional collection in northern Colombia (Fig. 2).

##### Notes.

**Figure 3.**
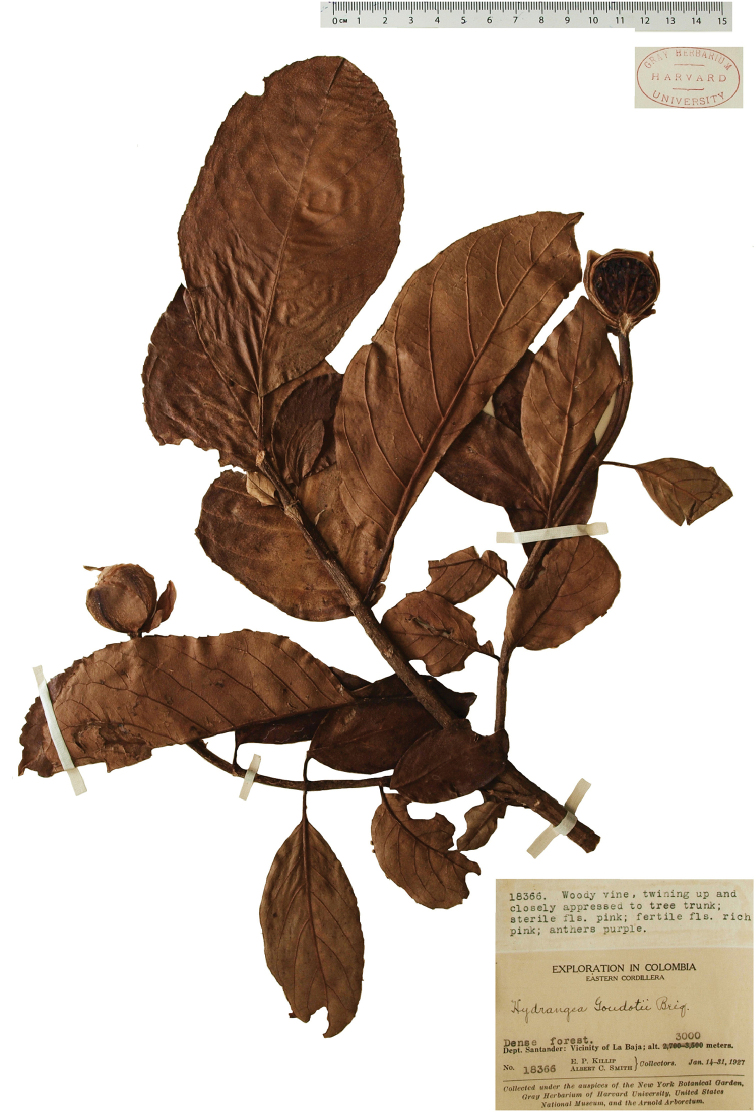
*Hydrangea durifolia*. Branch with distinct “vegetative” leaves, floral axes with leaves and inflorescence buds. Image of specimen *E.P. Killip & A.C. Smith 18366* (GH).

McClintock (1957) cited the type collection at G as “holotype”, but there are two specimens, as in the case of *H. caucana*, so we here designate a second-step lectotype with the better of the G sheets. She also mentioned the existence of a fragment and photo in F, but we have not seen these, despite a visit to this herbarium. Apart from the original syntype (*Holton 661*, G, with locality in the “regione neogranadino-bogotana”), there is only one other specimen (see details below) that with certainty belongs to this species amongst the material we have studied for this revision. The leaves of the type specimen and of the *Holton 661* specimen are part of the inflorescence, which complicates the recognition of this species, as “vegetative” leaves are important for a correct identification at species level. However, these leaves are present in the only additional specimen we could assign with certainty to this species (Fig. 3). We have noticed that we are only able to identify collections with mere inflorescences of *Cornidia* up to species level, after having studied many samples of a specific taxon, as the leaves on the inflorescence axis do not have easily definable species-specific characteristics in comparison with the “regular” leaves.

The herbaria COL and F each house a black and white photo of a specimen of the *Linden 1393* collection, presumably taken in the BR and G herbaria, respectively.

Because of the lack of material and therefore, the uncertainty about this species´ circumscription, it is currently not possible to present an amended description. However, we also have no elements to consider this species as a synonym of one of the other species treated here, although it is morphologically close to *H. oerstedii* Briq. Briquet (1919) distinguished *H. oerstedii* by the much less coriaceous leaves, the inflorescence which has twice the width, the lax cymes as opposed to the more compact ones in *H. durifolia*, the sepals of the marginal flowers being two or three times larger, more papyraceous, with more abundant joined and protruding veins, which we can confirm based on the type specimens of both taxa. The few specimens of *H. durifolia* are known from the Colombian departments of Norte de Santander and Santander, whereas *H. oerstedii* is distributed in Costa Rica and Panama. Exploring field work in forests at elevations of around 2000–3000 m in the departments of Norte de Santander and Santander in the area of Pamplona might lead to the rediscovery of this species and the material will be helpful to define its taxonomic status.

The phylogenetic relationships of *Hydrangea durifolia* are yet unknown as there was no fresh material available for our molecular study (Granados Mendoza et al. unpublished results).

##### Preliminary conservation status.

It is possible this species is Critically Endangered according to the IUCN categories and criteria (IUCN 2012), given its AAO of 8 km^2^, the existence of less than five locations and an extensive reduction in both EOO and AOO because of habitat destruction and fragmentation. However, we currently propose this species as Data Deficient, as its actual status is not well-known.

##### Additional specimen examined.

**Figure 4.**
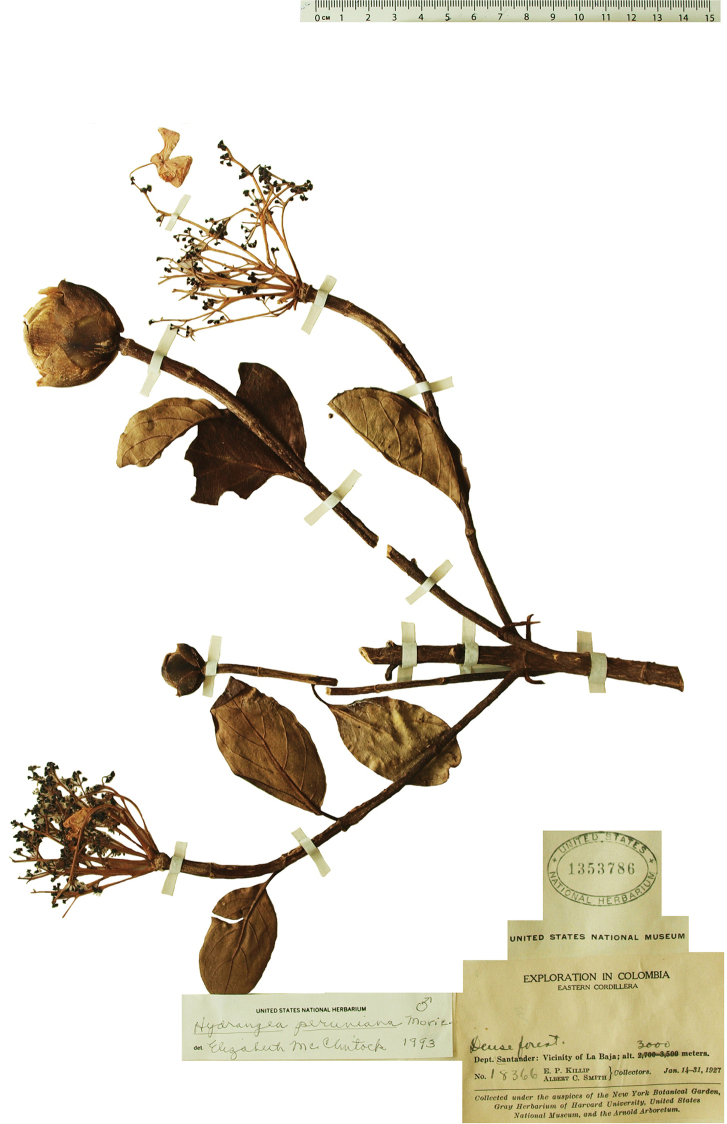
*Hydrangea durifolia*. Branch with inflorescence buds and inflorescences. Image of specimen *E.P. Killip & A.C. Smith 18366* (US).

**Colombia**. **Santander**: Vicinity of La Baja, 3000 m, 14–31 Jan 1927, ♂, inflorescence buds, flower buds, flowers, *Killip & Smith 18366* (GH (2), NY, US) (Figs 3–4).

#### 3. Hydrangea goudotii

Taxon classificationPlantaeCornaleHydrangeaceae

Briq., Annuaire Conserv. Jard. Bot. Genève 20: 404. 1919.

283A4545-1F61-553B-BB9D-511B2ADDF761

Figures 5 , 6

##### Type.

Colombia. Tolima: massif du Quindío á Portiohuelo, ♀, flower buds, flowers, *J. Goudot s.n.* (lectotype, designated by McClintock 1957, pg. 238 [as “holotype”]): G! [G00418961]; isolectotype: F! [F0066624F]).

##### Description.

**Figure 5.**
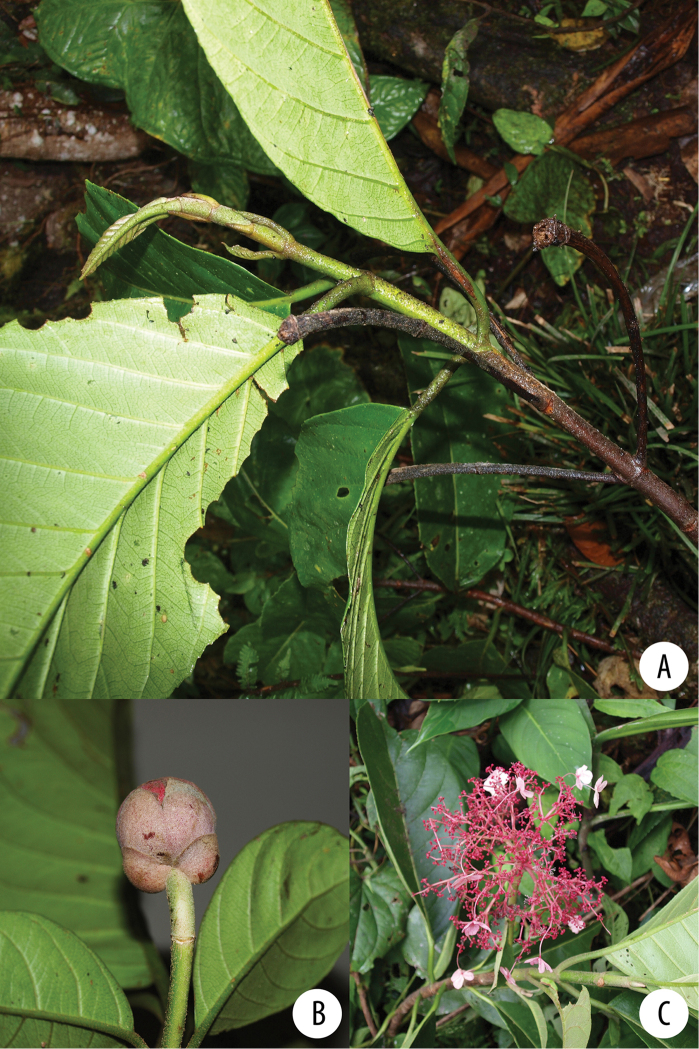
*Hydrangea goudotii***A** branch with leaves seen abaxially, old inflorescence axes and the vegetative portion of the flowering branch **B** inflorescence bud with cucullate inflorescence bracts and densely pubescent inflorescence axis **C** inflorescence of functionally male plant, with enlarged marginal flowers and a few flowers that still show stamens **A** field image of collection *Granados Mendoza et al. 2012-105***B, C** field images of collection *Granados Mendoza et al. 2012-43*.

Root-climbing liana of up to 10 m high; functionally dioecious; ***free-growing branches*** many-ribbed, slightly angular to quadrangular, apically with dense appressed white caducous stellate hairs; ***leaves*** papyraceous, decussate, petiole terete, rarely slightly quadrangular or sulcate adaxially, basally with broadly sulcate adaxially, color dark green, scarcely pubescent with small, white stellate hairs, 1.5–2.7 cm long, leaving a triangular scar on the branch when leaves shed; lamina elliptic to obovate, 14–25 cm long, 7–14 cm broad, base cuneate, slightly decurrent, sometimes asymmetric, apex mucronate to acuminate, leaf margin (widely) serrate, venation brochidodromous, veins 8–9 pairs, adaxial leaf side with midvein protruding along its whole length, primary and secondary veins also protruding, angle primary veins up to 50 degrees, secondary veins reticulate and connecting the primary veins resulting in a network with parallel secondary veins nearly perpendicular with respect to the primary veins, white stellate pubescence scarce, only near the leaf base, abaxially with protruding veins, opaque olive green, scarcely to densely pubescent with appressed stellate white hairs, the latter depending on the specimen, nearly sessile to shortly stalked, acarodomatia numerous, present in axils of midvein and primary veins in the lower 2/3 of the lamina, consisting of a simple cavity, glabrous or sometimes with stellate hairs in the entrance; ***inflorescence axis*** densely pubescent with appressed, white, stellate hairs (Fig. 5B), 6–15 cm long, gradually broadening towards the apex, with 2 opposite leaf pairs along the axis and one opposite kataphyll pair immediately below the inflorescence, deciduous, petiole 1 cm long, lamina 3.5–9.3 cm long, 2.5–6 cm broad, abaxially densely pubescent with white stellate hairs, ***apex of the floral axis*** woody, cone-shaped, elongated bract scars visible, thickening at the top, 8–9 mm broad, 5–6 mm high in functionally female plants, 6–7 mm broad, 2–2.5 mm high in functionally male plants, ***inflorescence bracts*** cucullate, dark green to light pink (Fig. 5B), coriaceous, abaxially densely pubescent with whitish stellate hairs, margin membranous and glabrous, veins not visible as a consequence of the pubescence, bracts increasing in size distally, consecutively and rapidly deciduous during inflorescence development, ***inflorescences*** lateral (Fig. 5C), opposite, 1–4 pairs of inflorescences per flowering branch, up to 3 decussate small leaf pairs (or their scars) between the two inflorescence pairs, flowering branch continues growing vegetatively very rapidly during inflorescence development, with already up to 4 decussate leaf pairs above the inflorescences when the upper inflorescences are still in bud, linear, with dense white stellate hairs, inflorescence umbellate, buds up to 3.4 cm broad and 3 cm high before opening, in flowering stage 5–12 cm diameter, 2.5–8 cm high, with 5–10 main axes in functionally male plants, 5–6 main axes in functionally female plants, partial inflorescences umbels, secondary and tertiary inflorescence axes with reddish-white, appressed, stellate hairs; ***enlarged marginal flowers*** always present (Fig. 5C), terminally placed in a cyme, up to 3.6 cm diameter in female plants, up to 1 cm in male plants, sepals with marked veins, pistils 2 or sometimes not at all developed, reduced stigma not developed, receptacle rudimentary, globose, nearly the same size as that of reduced flowers, further characters not observed in detail, pedicel in male plants up to 1.5 cm; ***flower pedicel of reduced flowers*** 0.1–2.2 mm long in functionally male flowers, 1 mm long in functionally female flowers, enlarging during fruit development, 1.5 mm long in mature fruits, ***receptacle*** broadly campanulate in functionally male flowers, semiglobose in functionally female flowers, ***ovary*** inferior, ***calyx lobes*** 4, triangular, 0.3–0.5 mm long, ***petals*** 4, pinkish to wine red, imbricate, cucullate, membranous, 2 mm long, 1 mm broad; ***functionally male flowers*** (Fig. 5C): hypanthium 1 mm diameter, 1.5 mm high, stamens 8, well-developed, filaments 1–1.5 mm long, anthers 1 mm long, 0.2 mm broad, pistils 2, reduced, 0.1 mm long, stigmas not penicellate; ***functionally female flowers***: hypanthium 1.5 mm diameter, 1–1.5 mm high, stamens extremely reduced, as such their number could not be determined and their filaments and anthers have not been measured, pistils 2, 1 mm long, enlarging up to 1.5 mm during fruit maturation, stigmas apically clavate and shortly penicellate, 0.2 mm long; ***fruit*** a semiglobose capsule, apically with a conspicuous border, dark reddish brown, 2 mm high, 2.5 mm broad above, 3 mm long, opening between the two pistils to release seeds, seeds not seen.

##### Distribution.

*Hydrangea goudotii* is endemic to Colombia and Ecuador. Further exploration for this species throughout both countries is required, as its currently known distribution pattern is fragmentary.

##### Habitat.

This species occurs in mountain cloud forest, sometimes on heavy slopes at elevations between 1000–2500 m.

##### Phenology.

This species has been collected in flower and fruit in January, March, June, July, August, and December. Although further studies on its biology are needed, it is likely that *H. goudotii* is characterized by two distinct flowering and fruiting periods: December–March and June–August. However, it remains to be investigated whether these two periods take place each year.

##### Notes.

Briquet (1919) based his description of *H. goudotii* on three collections, all by Goudot (without number): “Columbia: Ibaque (Ibagué), Cahi (Cali) [G00223928, G00418963]; rives du Combayma (Combeima) [G00418962]; massif du Quindiu (Quindio), a Portoihuelo (?) [G00418961]” (correct current locality names and G barcode numbers are placed between brackets). It seems all three collections are from the Central Cordillera between Ibagué and Armenia in the department of Tolima (R. Callejas, Universidad de Antioquia, Colombia, pers. comm.). McClintock (1957) selected the latter of the three syntypes mentioned in the original description by Briquet (1919) as lectotype, although she referred to it as holotype which is not correct according to the ICN (Art. 9, Turland et al. 2018). However, as in several other species for which McClintock (1957) realized lectotypifications and synonimizations, we doubt that she actually has had access to the involved specimens. In the case of *H. goudotii*, she considered the most representative specimen to be the collection which has been fragmented to take material to the herbarium F. Indeed, the specimen in F [F0066624F] consists, apart from the fragments of leaves and inflorescences, of a photo of the original Goudot collection. Careful examination of the latter shows that it consists of the branch which was designated as lectotype by McClintock (1957) [G00418961], which has been turned around 180 degrees, and an additional branch which was fragmented and is the F specimen. The latter is considered as isolectotype in that herbarium. However, because of the fragmentation of the original collection, the material that remained in G does lack fully developed enlarged marginal flowers.

*Hydrangea goudotii* should not be considered a synonym of *H. oerstedii* or H. peruviana var. oerstedii from which it can be distinguished by the appressed white caducous stellate pubescence on free-growing branches, these being glabrous in *H. oerstedii*. Moreover, *H. goudotii* is currently only known from Colombia and Ecuador, whereas *H. oerstedii* occurs in Costa Rica and Panama.

According to our molecular study, *Hydrangea goudotii* is closely related to *H. trianae* (Granados Mendoza et al. unpublished results).

##### Preliminary conservation status.

Although this species has an EOO of about 154,700 km^2^, it is Endangered according to the IUCN categories and criteria (IUCN 2012), with an AAO of 100 km^2^, as well as an extensive reduction in both EOO and AOO due to habitat destruction and fragmentation.

##### Additional specimens examined.

**Colombia. Antioquia**: Urrao, Vereda Calles, Parque Nacional Natural “Las Orquídeas’, margen derecha del Río Calles, en el filo NW de la Cabaña de Calles, 1450 m, 6 Dec 1993, ♀, inflorescence buds, flower buds, *Cogollo et al. 7857* (MO); Zona limítrofe del Parque Nacional Natural Las Orquídeas, vereda Calles, Alto de Palmitas, ca 1 km de la Cabaña de Calles del INDERENA, 6°32'N, 76°19'W, 1300–1400 m, 1 Dec 1993, inflorescence bud, *Pipoly et al. 17468* (MO); municipio Urrao, carretera Urrao–Caicedo, 1 km antes del Alto de Caicedo, bosques a lo largo de Quebrada Villa Riaga, 6°28'N, 76°10'W, 2180 m, 5 Dec 1986, ♀, fruits, *Callejas et al. 3165* (F, HUA); Alto de Cuevas, 10 km W of Blanquita, 12 km W of Nutibara, transect B, 1720 m, 3 Mar 1992, ♂, flowers, *Gentry et al. 76116* (MO); municipio de Venecia, Vereda El Rincón, camino a Cerro Bravo, 5°56'19"N, 75°42'17"W, 1600–2300 m, 5 Jul 2007, flower buds, *David et al. 2192* (HUA); La Ceja, 2300 m, Sep 1964, ♀, fruits, *Espinal 1777* (COL). **Caldas**: Pueblo Rico, La Selva, 1400 m, 4 Jan 1946, ♀, flower buds, fruits, *von Sneidern 5265* (AAU, C, F, GH, MICH, US); Mcpio. Pensilvania, Vereda Líbano, al lado del camino del puente de Líbano, ca. 2400 m, 11 Jul 1982, ♀, flowers, *Albert de Escobar & Brand 2095* (HUA); **Cauca**: municipio El Tambo, Correg. La Romelia, km 75 vía a La Gallera, 1700–2000 m, 29 Jan 1995, ♂, flower buds, flowers, *Ruiz et al. 361* (COL); **Nariño**: en la quebradita de El Osa, 1900 m, 8 Jan 1949, ♂, flowers, *Uribe 1913* (COL); **Risaralda**: municipio de Santuario, estribación Oriental de la Cordillera Occidental, transecto de las Colonias, hacia Alto del Tigre, 2500 m, 31 Jan 1983, ♀, fruits, *Torres et al. 1359* (COL).

**Figure 6.**
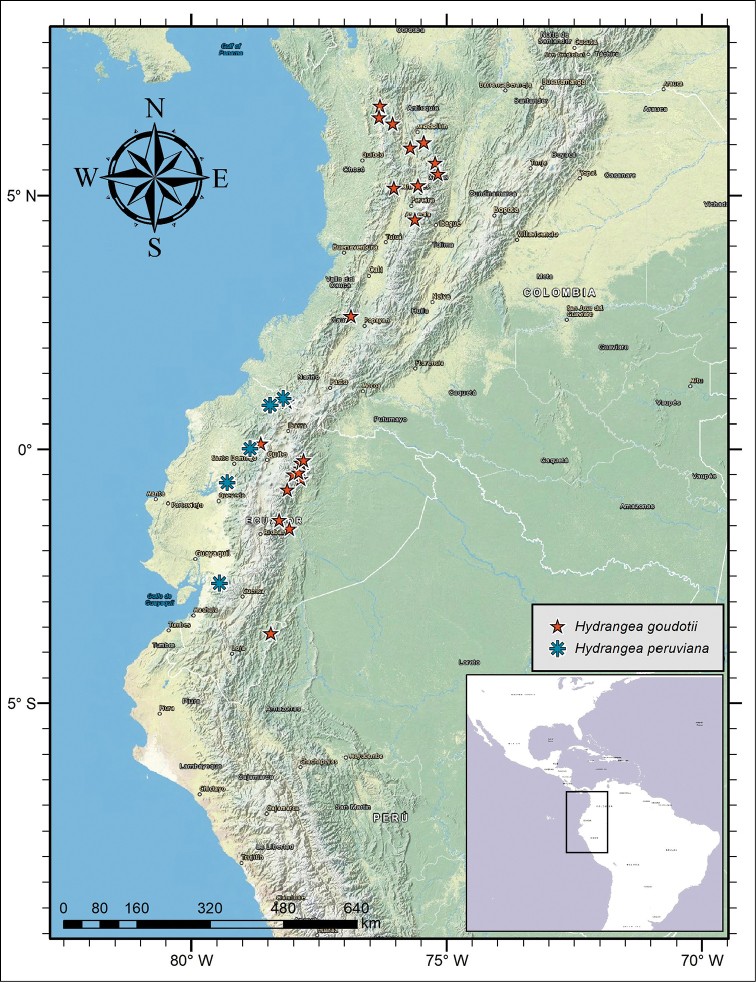
Distribution of *Hydrangea goudotii* and *Hydrangea peruviana*.

**Ecuador. Carchi**: near Maldonado, 1400 m, 30 Jul 1989, ♂, flowers, *van der Werff & Gudiño 10778* (QCNE); **Napo**: San Francisco de Borja, small forest high above banks of river beyond meadow at S. E. of town, hotel and square, 6 Jul 1980, ♂, flowers, *Sobel 2392* (NY); vicinity of Baeza, 1900 m, 27 Mar 1972, ♀, flowers, fruits, *Dwyer & McBryde 9601* (QCA); carretera El Chaco–Oyacachi, cerca del Río San Juan Grande, 0°16'S, 77°51'W, 1730 m, 15 Dec 1993, ♀, fruits, *Freire-Fierro & Yánez 2694* (QCA); El Chaco, fincas en la parte Este del pueblo, 0°13'S, 77°48'W, 1700–2000 m, 15 Mar 1991, ♂, flowers, *Gavilanes & Quezada 470* (AAU, QCA); Cerro Antisana, montane forest 2 mi. S. E. Borja, 5700 ft, 3 Aug 1960, ♀, flowers, *Grubb 1216* (K, NY); Mera, surroundings of Sevilla de Oro, 6.9 airline km S [of] Shell, 1°33'40.5"S, 78°4'29.3"W, 1080 m, 15 Jun 2012, inflorescence bud, *Granados Mendoza et al. 2012-43* (GENT, HOXA, IEB, MEXU, QCNE); El Chaco, ca. 2.1 airline km W of Sardinas, 0°23'2.7"S, 77°51'23.9"W, 1996 m, 10 Jul 2012, inflorescence bud, *Granados Mendoza et al. 2012-105* (GENT, HOXA, IEB, MEXU, QCNE); same data as preceding, ca. 2 airline km W of Sardinas, 0°23'1.3"S, 77°51'22.4"W, 1987 m, 10 Jul 2012, sterile, old inflorescences axes of previous year visible, *Granados Mendoza et al. 2012-107* (GENT, HOXA, IEB, MEXU, QCNE); Tena, Cordillera de los Huacamayos, localidad Pigui Yacu (Verde Yacu) Río Cushillo Yacu (Río Grande se origina en la cabecera de Sisahua), 1620 m, 30 Dec 1995, ♀, flowers, *Jaramillo & Tapia 18583* (QCA); Quijos, Cosanga. Cerca de la población de Cosanga, a 500 m del Río Cosanga, 2000 m, 11 May 1990, ♂, flowers, *Palacios 4895* (MO, QCNE); **Pichincha**: Nanegal, Sendero de la Unión de los Ríos, secondary lower montane rain forest along Río Umachaca, 00°07.5'N, 78°37.5'W, 1450 m, 24 Jun 1996, ♂, flowers, *Kelch et al. 31803* (QCNE); Quito, Parroquia Nanegal, montane rain forest along trail from Río Umachaca to Loma Sta. Lucía, c. 6 km airline SE of Nanegal, near top ridge, 00°07.5'N, 78°37'W, 1635 m, 6 Sep 1993, inflorescence buds, *Webster et al. 30392* (QCNE); Quito, Nanegal, Reserva Biológica Maquipucuna, 1200–1700 m, 20 May 1991, ♀, flowers, *Tipaz & Quelal 207* (AAU, MO, NY, QCNE); Quito, Maquipucuna Reserve, trail to Hacienda Esparragos, 00°07'N, 78°38'W, c. 1400 m, 27 Aug 1989, ♂, flowers, *Webster et al. 27062* (QCA); **Tungurahua**: Baños, ca. 2 km E of Río Verde on touristic path before tunnel on road to Baños–Mera, 1°23'25.1"S, 78°16'51.2"W, 1721 m, 16 Jun 2012, sterile, *Granados Mendoza et al. 2012-45* (GENT, HOXA, IEB, MEXU, QCNE); **Zamore-Chinchipe**: in the vicinity of the mining camp, at the Río Tundaime, along road to military base El Condor, 3°37'31"S, 78°26'26"W, 1000 m, 5 Nov 2004, ♀, fruits, *van der Werff et al. 19315* (LOJA, QCNE).

#### 4. Hydrangea oerstedii

Taxon classificationPlantaeCornaleHydrangeaceae

Briq., Annuaire Conserv. Jard. Bot. Genève 20: 407–408. 1919.

A6A06BF8-69D7-5127-A5F4-3C53C7EAB501

Figures 7 , 8

Cornidia radiata Oerst., Vidensk. Meddel. Naturhist. Foren. Kjobenhaven 1856: 42. 1856. Type. Costa Rica. Cartago: in monte Candelaria, 6000–7000 ft, Feb 1847, ♂, flowers, *A.S. Oersted 1782* (lectotype, designated by McClintock 1957, pg. 238 [as “holotype”]: C!, isolectotypes: F! [V0066575F], US! [00097007]).
Hydrangea peruviana var. oerstedii (Briq.) Freire Fierro, Fl. Ecuador 73: 34. 2004. Type. Based on Hydrangea oerstedii Briq.

##### Type.

Based on (replacement name for) *Cornidia radiata* Oerst.

##### Description.

**Figure 7.**
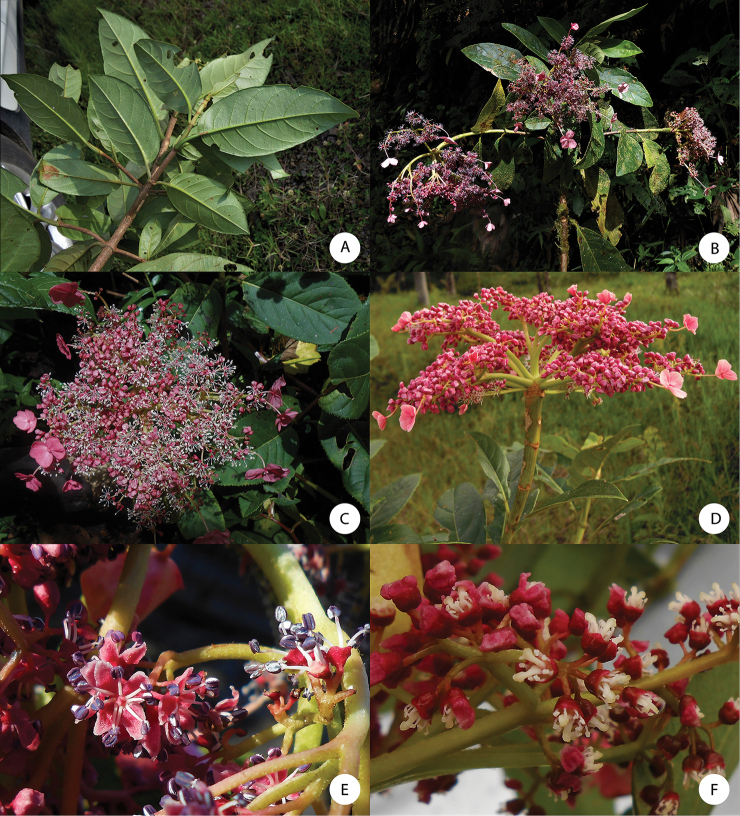
*Hydrangea oerstedii***A** branch with leaves seen abaxially **B** flowering branch of functionally male plant with three inflorescences with enlarged marginal flowers and many flowers with large stamens **C** inflorescence of functionally male plant with enlarged marginal flowers, most flowers in bud with petals visible, and a few open flowers with long stamens **D** close-up of functionally male inflorescence **E** close-up of functionally male flowers with reduced pistils **F** close-up of functionally female flowers with reduced stamens and large pistils. **A** field image of collection *Samain & Martínez 2012-044***B** field image of collection *Samain & Martínez 2013-025***C** field image of collection *Samain & Martínez 2012-044***D** field image of collection *Samain et al. 2019-003***E** field image of collection *Samain & Martínez 2013-025***F** field image of collection *Samain & Martínez 2013-021*.

Root-climbing liana of up to 60 m high, often bending downwards, functionally dioecious; ***free-growing branches*** quadrangular, glabrous (Fig. 7A); ***leaves*** decussate, petiole flattened or sulcate adaxially, terete abaxially, color dark green, pubescent with caducous, appressed, white stellate hairs, 1.5–3 cm long, leaving a rhomboid scar on the branch when leaves fall; lamina flat, ovate to lanceolate-elliptic, 8–25 cm long, 4–14 cm broad, base cuneate, apex acuminate, leaf margin glandular-dentate, venation brochidodromous, veins 6–9 pairs, adaxial leaf side with midvein and primary veins sunken, secondary veins slightly visible, primary veins joining to form a submarginal or intramarginal vein, pubescent with caducous, appressed, white, stellate hairs, older leaves glabrous, abaxially with protruding veins, secondary veins connecting the primary veins, opaque olive green, densely pubescent with caducous, appressed, white, stellate hairs, acarodomatia numerous, consisting of simple cavities, in axils of midvein and primary veins; ***inflorescence axis*** angular, pubescent with caducous, appressed, white, stellate hairs, 8–42 cm long, with 3–6 decussate leaf below the inflorescence, persistent, petiole 1–1.5 cm long, lamina 6–13 cm long, 2.5–8 cm broad, ***apex of the floral axis*** woody, cone-shaped, elongated bract scars visible, thickening at the top, 6–11 mm broad, 3–7 mm high in functionally female plants, 10 mm broad, 5 mm high in functionally male plants, ***inflorescence bracts*** cucullate, cream-colored, coriaceous, abaxial surface tubercled, margin pubescent, adaxially white pubescent, margin membranous, veins slightly marked, bracts increasing in size, lowermost bract 3 cm large, 4 cm broad, higher bracts up to 5 cm large, 5.5 cm broad, consecutively and rapidly deciduous during inflorescence development, ***inflorescences*** lateral, decussate, 1–4 pairs of inflorescences per flowering branch (Fig. 7B), sometimes only one inflorescence developing, flowering branch continues growing vegetatively with the same vigor and periodicity above the inflorescences as below these, inflorescence axes with basal lignified parts of inflorescences of previous years visible in well-collected specimens, allowing to observe growth and flower periodicity, these rests 18–51 cm apart, with 5–11 decussate leaf pairs, but constant within one specimen (*Samain et al. 2012-047*, *Samain et al. 2012-072*), medulla central in the branch, disappearing in older branches, leaving a hole only, inflorescence umbellate, buds up to 4–5 cm broad and 3.5–4 cm high before opening, in flowering stage 6–28 cm diameter, 4–17 cm high, with 8 main axes in functionally male plants (Fig. 7C, D), 5–14 main axes in functionally female plants (Fig. 7F), partial inflorescences cymes, lax, secondary and tertiary inflorescence axes towards the exterior glabrous, towards its center densely pubescent with whitish stellate hairs; ***enlarged marginal flowers*** generally present (Fig. 7B–D), rarely absent (e.g. *Haber 1511*), terminally placed in a cyme, sepals 4, pinkish red to wine red, whitish in old inflorescence and infructescences, separate at their base, with marked veins, palmate, pistils 2 or reduced, central part of the flower often amorph, 1–1.5 cm diameter, growing up to 4 cm diameter during flower development, further characters not observed in detail, pedicel 1–4 cm long; ***flower pedicel of reduced flowers*** (0.2–)0.5–2(–4) mm long in functionally male flowers, 2–0.5(–2) mm long in functionally female flowers, ***receptacle*** broadly triangular in functionally male flowers, semiglobose to semirectangular in functionally female flowers, ***ovary*** inferior, ***calyx lobes*** 4, triangular, 0.2–0.3 mm long, 0.8 mm broad, ***petals*** 4, pinkish red to purple, valvate, cucullate, papyraceous, 1–2 mm long, 0.6–1.0 mm broad; ***functionally male flowers*** (Fig. 7E): hypanthium 1.5–2.0 mm diameter, stamens 8, well-developed, filaments 1.5–3 mm long, anthers 0.8–1.2 mm long, 0.5–0.8 mm broad, pistils 2, reduced, 0.5 mm long, transparent, stigmas not penicellate; ***functionally female flowers*** (Fig. 7F): hypanthium 1.2–2 mm diameter, 1 mm high, stamens 8, reduced, filaments 0.5 mm long, anthers 0.5 mm long, 0.2 mm broad, pistils 2, 1.2–1.5 mm long, rarely enlarging up to 2 mm during fruit maturation, stigmas apically clavate and shortly penicellate; ***fruit*** a semiglobose to semirectangular capsule, apically with a conspicuous, thickened border, ribs 4–6, dark brown with dark reddish brown margin, 1.8–3 mm high, 1.8–3 mm broad above, 1.5–2.5 mm diameter, opening between the two pistils to release seeds, seeds not seen.

##### Distribution.

**Figure 8.**
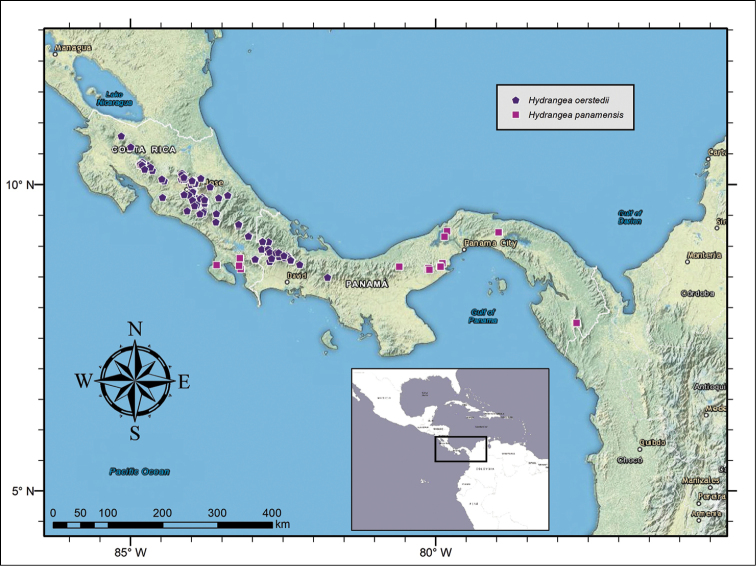
Distribution of *Hydrangea oerstedii* and *Hydrangea panamensis*.

*Hydrangea oerstedii* is very abundant in Costa Rica and Panama. It is the most common species of this study (Fig. 8).

##### Habitat.

This species occurs in mountain cloud forest at elevations between 1100 and 2500 m. In contrast to most of the other *Hydrangea* species, it is common to find this species in disturbed habitats, even when there is no primary forest left and only the very humid microhabitat and protection against direct sunlight remain.

##### Phenology.

This species has been collected with flowers and fruits throughout the year.

##### Notes.

Not to be considered as a variety of *H. peruviana*, from which it can be distinguished by the flat, ovate to lanceolate-elliptic leaves with white pubescence abaxially and a glandular dentate margin. In contrast, *H. peruviana* is characterized by very slightly spoon-shaped, elliptic to slightly obovate leaves with reddish pubescence adaxially and a serrate to slightly dentate margin. Moreover, *H. oerstedii* is a relatively common species in Costa Rica and Panama, whereas *H. peruviana* is a very rare species from Ecuador.

Freire Fierro (2004) also mentioned the presence of an isotype in K, but although this specimen bears the same locality data on its label as the type, it cannot be confirmed that it concerns the same collection, given that no number is mentioned.

According to our molecular study, *Hydrangea oerstedii* is not related to any of the species included in this study (Granados Mendoza et al. unpublished results).

##### Preliminary conservation status.

With an EOO of slightly more than 20,760 km^2^ and an AAO of 428 km^2^, this species is Near Threatened according to the IUCN categories and criteria (IUCN 2012). Although its habitat is continuously being destroyed and fragmented, this species is robust, and basically only needs a host tree that remains of the primary forest. As the older plants bend downwards and often have their eye-catching inflorescences just a few meters above ground level, it is even possible that this species is favored when trees are being logged. Nevertheless, should large areas of forest be destroyed, this species may well enter into a threatened category.

##### Additional specimens examined.

**Costa Rica. Alajuela**: Alajuela, Distrito 3° Carrizal, 13.1 km N of Heredia via Rte. 114 (10.9 km. N of Barva), 20 ft. from rd., 10°5'26.4"N, 84°9'56.71"W, 1500–2000 m, 15 May 1990, ♀, fruits, *Grantham 90.0433 & Parsons* (CAS); San Ramón, Distrito 1° San Ramón, Vicinity of San Ramón, La Palma de San Ramón, 14 Dec 1926, ♀, fruits, *Brenes 5221* (CR, NY); R.B. Monteverde, Cordillera de Tilarán, Sendero Rancho Alegre, 10°17'40"N, 84°45'W, 1000 m, 4 Apr 1995, ♀, fruits, *Martínez 379* (CR, MO); La Palma, Picada San Antonio, 1 Dec 1922, ♀, fruits, *Brenes 3807* (CR, F, GH); Reserva Biológica Bosque Nuboso de Monteverde, al lado del Sendero Chomogo, 1.15 km al NE de la caseta de entrada, 10°18'22.23"N, 84°47'11.12"W, 1710 m, 2 Aug 2012, ♀, fruits, *Samain & Martínez 2012-045bis* (CR, GENT, IEB, MEXU); Peñas Blancas, R.B. Monteverde, Peñas Blancas River Valley, Atlantic slope, 10°20'N, 84°50'W, 1250 m, 12 Oct 1985, ♂, flowers, *Haber 3102* (CR); Reserva Biológica Bosque Nuboso de Monteverde, al lado del Sendero Camino, 10°17'59.87"N, 84°46'58.35"W, 21 Jan 2013, 1519 m, ♂, flowers, *Samain & Martínez 2013-025* (CR, GENT, IEB, MEXU); cerca de División continental (Ventana), 1550–1580 m, 14 Dec 1976, ♀, flower buds, fruits, *Dryer 1080* (CR, F, MO); Monteverde Reserve, Atlantic Slope, Río Peñas Blancas valley, ridge trail above Quebrada Leona, 1050–1300 m, 17 Jan 1985, ♀, flowers, fruits, *Haber 1304* (MO); Monteverde Reserve, Atlantic Slope, Río Peñas Blancas valley, 960 m, 19 Nov 1984, ♀, fruits, *Haber 1035* (MO); same data as preceding, 1300 m, 20 Mar 1985, ♀, flower buds, *Haber 1511* (MO); Reserva Biológica Monteverde, Río Peñas Blancas, Parcela de Rockwell, 10°19'N, 84°43'W, 820 m, 21 Dec 1988, ♂, flowers, *Bello 632* (CR, MO); Alajuela, no protegida, Cuenca del Sarapiquí, Hotel Posada Volcán Poás, Viento fresco, 10°10'27.81"N, 84°10'10.072"W, 1800 m, *13 Mar 2003*, ♀, old fruits, *Kriebel 2911* (CR); San Ramón, Cerros de la Palma, 1500 m, 24 Jan 1984, ♀, fruits, *Gómez-Laurito 9802* (CR, F); San Ramón, La Palma, 13 Mar 1929, ♀, fruits, *Brenes 19368* (CR); La Palma, Picada de San Antonio, Dec 1922, ♀, old fruits, *Brenes 19366* (CR); La Palma de San Ramón, 22 Dec 1922, ♀, old fruits, *Brenes 19367* (CR); Bosque Húmedo en San Pedro de San Ramón, 11 Jan 1924, ♀, old fruits, *Brenes 19370* (CR); La Palma, Picada de San Antonio, Dec 1922, ♀, old fruits, *Brenes 19371* (CR); San Ramón, Bosque húmedo de las colonias de San Pedro, 1200 m, 11 Jan 1924, ♀, fruits, *Brenes 3984* (F); **Cartago**: Cartago, Distrito 5° San Francisco, 0.5 km N. of Tapantí, 9°47'56.64"N, 83°55'37.57"W, 1300 m, 7 Nov 1971, ♀, young fruits, *Lent 2210* (F, GB, US); El Guarco, Distrito 2° San Isidro, dry wooded slopes about 13 km south of the Taras Intersection along the Interamerican Highway or about 18 km north of El Empalme, 9°47'52.05"N, 83°56'49.10"W, 4 Jul 1976, ♂, old flowers, *Wilbur 19852* (DUKE); Empalme, Cartago, 9°43'5.91"N, 83°56'50.32"W, 2300 m, 1 Dec 1983, ♂, flower buds, flowers, *Gómez 22176* (CR, F, MO, UC); Cartago, slopes in the vicinity of Palo Verde and the Quebrada Cangreja on the road to Estrella, 9°47'0.07"N, 83°57'11.48"W, 1600 m, 19 Dec 1974, ♀, fruits, *Wilbur 18437 & Luteyn* (CAS, DUKE, F); Turrialba, Distrito 8° Tayutic, Turrialba, Jicotea, por la fila al lado oeste del Río Jicotea, Finca del señor Israel Martínez, 9°47'5"N, 83°33'15"W, 1100–1200 m, 7 Dec 1994, ♀, fruits, *Sánchez et al. 424* (CR, F, K); Cartago, hills above Platanillo, 9°49'31.24"N, 83°24'16.91"W, 915 m, 7 Nov 1967, ♂, old flowers, *Stone 2324* (DUKE); Paraíso, Distrito 3° Orosi, about 15 km south of Tapantí along the new road, on the east slope above the Río Grande de Orosi near the concrete bridge, 9°46'44.28"N, 83°47'46.78"W, 1500 m, 30 Dec 1970, ♀, fruits, *Burger 7525 & Liesner* (F, GB); Cartago, Distrito 5° San Francisco, about 3–5 km S of Muñeco along the Río Sombrero, 9°45'38.34"N, 83°54'21.62"W, 1400–1500 m, 25 Feb 1978, ♀, fruits, *Wilbur 24976* (DUKE); Cartago, ca. 2.5 km S of Muñeco, 9°46'15.95"N, 83°54'17.34"W, 1500 m, 25 Feb 1978, ♀, fruits, *Almeda & Nakai 3937* (CAS, CR); El Guarco, Distrito 2° San Isidro, Alto de La Estrella, 26–27 Mar 1924, ♀, fruits, *Standley 39080* (US); Cartago, al lado del camino Copatchi–San Rafaël Arriba, 9°49'47.52"N, 84°2'22.83"W, 1838 m, 27 Jul 2012, sterile, *Samain & Martínez 2012-034* (CR, GENT, IEB, MEXU); al lado del camino Copatchi–Quebradilla, 9°49'54.96"N, 84°1'38.37"W, 1931 m, 27 Jul 2012, sterile, *Samain & Martínez 2012–035* (CR, GENT, IEB, MEXU); El Guarco, Cuenca del Reventazón, San Isidro, Ruta al Cerro de la Muerte, entrada a Palo Verde, 1 km después de la entrada, 9°47'56"N, 83°56'51"W, 1800 m, 26 Nov 1998, inflorescence buds, *Rodríguez et al. 4208* (CR, MO); camino San Isidro de El General–Cartago, 9°41'23.46"N, 83°54'12.64"W, 2532 m, 11 Aug 2012, ♀, fruits, *Samain & Martínez 2012-072* (CR, GENT, IEB, MEXU); camino Cartago–San Isidro de El General, 9°46'16.46"N, 83°59'1.03"W, 1770 m, 23 Jan 2013, ♂, flowers, *Samain & Martínez 2013-030* (CR, GENT, IEB, MEXU); same data as preceding, 9°46'16.46"N, 83°59'1.03"W, 1769 m, 23 Jan 2013, ♀, fruits, *Samain & Martínez 2013-031* (CR, GENT, IEB, MEXU); same data as preceding, 9°41'23.72"N, 83°54'12.58"W, 2489 m, 23 Jan 2013, ♀, fruits, *Samain & Martínez 2013-032* (CR, GENT, IEB, MEXU); Paraíso, P.N. Tapantí, Valle del Reventazón, Río Grande de Orosi, fila innominada al sur del cruce de la toma del Río Humo, 9°43'5"N, 83°46'50"W, 1500–1600 m, 21 Jun 1996, floral buds, *Morales 5394 et al.* (CR, MO); Cartago, Paraíso, Cordillera de Talamanca, Río Grande de Orosí, Tapantí, bosque alterado por crecientes del Río Villegas, cerca de la toma de agua, 9°41'50"N, 83°46'50"W, 1500 m, 22 Jul 1994, ♂, old flowers, *Morales 3059* (CR, F); Cartago, El Guarco, No protegida, Cuenca del Pirrís–Damas, Cerros de la Muerte, 9°47'47.6085"N, 83°59'54.3782"W, 2100 m, 18 Feb 2002, ♂, young flowers, *González 1528* (CR); Cartago, Paraíso, P.N. Tapantí, Valle del Reventazón, Estación Tapantí, Sendero La Heliconia, 9°45'20"N, 83°47'W, 1300 m,10 Jun 1995, ♂, old flowers, *Mora 661* (CR); Cartago, Paraíso, P.N. Tapantí–Macizo de la Muerte, Cuenca del Reventazón, P.N. Tapantí, Sendero Árboles Caídos, 9°45'4"N, 83°47"W, 1300–1413 m, 18 Jun 2005, ♂, old flowers, *Morales 13225* (CR); Cartago, Paraíso, No protegida, Cuenca del Reventazón, entrada a Monte Sky, 9°44'53.9065"N, 83°50'4.8525"W, 1537 m, 5 Dec 2000, ♀, fruits, *González 1225* (CR); Cartago, Paraíso, P.N. Tapantí, Valle del Reventazón, Sector Dos Amigos, Sendero Rancho Negro, 9°45'20"N, 83°47'W, 1300 m, 20 Jul 1994, floral buds, *Morales 289* (CR); Cartago, La Unión, San Rafael, Cerros de La Carpintera, Campo Iztarú de los Scouts, Alrededores del área administrativa, 9°53'24.8"N, 83°58'6.8"W, 1730 m, 13 Oct 2010, ♂, old flowers, *Cascante 2250* (CR); Prov. de Cartago, La Unión, Tres Ríos, 1350 m, 12 Apr 1953, ♀, fruits, *Córdoba 327* (CR): Cartago, San Nicolás, Cerros de la Carpintera, Campo Escuela Iztarú, propiedad de la Asociación Scouts de Costa Rica, sendero hacia la cima de los cerros, 9°52'55.8"N, 83°58'40.3"W, 1854 m, 11 Jun 2007, ♀, fruits, *Estrada 3977* (CR); Cartago, Paraíso, Orosí, Camino del pueblo de Río Macho al Embalse, área de cafetales y pequeños bosques alterados, 9°46'15"N, 83°51'15"W, 1500 m, 26 Jan 2010, ♀, fruits, *Cascante 2179* (CR); Tapantí, 1300 m, 15 Jul 1937, floral buds, *Valerio 1658* (F); Cartago, very steep slopes with open forest and many epiphytes about 10 km south of Tapantí along the new road on the east slope above th Río Grande de Orosí, 9°42'N, 83°47'W, 1400–1600 m, 10–24 Jun 1968, ♀, fruits, *Burger & Stolze 6108* (F); Turrialba, Las Cortinas 1 km al norte del Monumento Guayabo, Manglares del Río Guayabo, 9°57'50"N, 83°41'30"W, 1100 m, 13 Oct 1992, ♀, fruits, *Rivera 2007* (CR, F, MO); Refugio Nacional de Vida Silvestre Tapantí, about 7–15 km beyond the main entrance to the reserve; roadsides and trails through wet montane rainforest, 1500–1650 m, 3 Mar 1991, ♀, fruits, *Almeda et al. 6912* (CR, MO); **Guanacaste**: Tilarán, Área no protegida, Cordillera de Tilarán, Reserva Biológica del Colegio de Monteverde, 10°21'20"N, 84°49'50"W, 1600–1700 m, 10 Jan 1992, ♀, fruits, *Bello 4328* (CR, MO); Z.P. Tenorio, Cordillera V Tilarán, Tierras Morenas, Río San Lorenzo, 1050 m, 10°36'40"N, 84°59'45"W, 10 May 1994, ♂, old flowers, *Rodríguez 13* (CR, MO); Santa Elena, cerca de la entrada del Parque Selvatura, 10°20'31.23"N, 84°47'56.94"W, 1618 m, 1 Aug 2012, ♂, *Samain & Martínez 2012-044* (CR, GENT, IEB, MEXU); Santa Elena, en el límite de la Reserva Bosque Nuboso Santa Elena, 10°20'35.12"N, 84°47'50.53”W, 1659 m, 31 Jul 2012, ♀, fruits, *Samain & Martínez 2012-045* (CR, GENT, IEB, MEXU); Reserva Bosque Nuboso Santa Elena, al lado del camino a la Laguna de Arenal, 827 m al NE de la caseta de entrada, 10°20'58.70"N, 84°47'33.56"W, 1582 m, 31 Jul 2012, ♀, fruits, *Samain & Martínez 2012-046* (CR, GENT, IEB, MEXU); Reserva Bosque Nuboso Santa Elena, al lado del camino a la Laguna de Arenal, 1.27 km al NE de la caseta de entrada, 10°21'14.67"N, 84°47'34.77"W, 1555 m, 31 Jul 2012, ♀, old inflorescence axes, fruits, *Samain & Martínez 2012-047* (CR, GENT, IEB, MEXU); Reserva Bosque Nuboso Santa Elena, al lado del camino a la Laguna de Arenal, 415 m al NE de la caseta de entrada, 10°20'48.66"N, 84°47'40.36"W, 1643 m, 31 Jul 2012, ♀, fruits, *Samain & Martínez 2012-048* (CR, GENT, IEB, MEXU); al lado del camino de Santa Elena al Mirador Lodge, 1.01 km desde la desviación hacia la Reserva Bosque Nuboso Santa Elena, 10°21'9.35"N, 84°48'6.97"W, 1545 m, 20 Jan 2013, inflorescence buds, floral buds, *Samain & Martínez 2013-019* (CR, GENT, IEB, MEXU); al lado del camino de Santa Elena al Mirador Lodge, 1.74 km desde la desviación hacia la Reserva Bosque Nuboso Santa Elena, 10°21'32.08"N, 84°48'9.79"W, 1495 m, 20 Jan 2013, ♀, inflorescence buds, flowers, *Samain & Martínez 2013-021* (CR, GENT, IEB, MEXU); al lado del camino de Santa Elena al Mirador Lodge, 2,069 m desde la desviación hacia la Reserva Bosque Nuboso Santa Elena, 10°21'43.64"N, 84°48'16.32"W, 1509 m, 20 Jan 2013, ♀, fruits, *Samain & Martínez 2013-022* (CR, GENT, IEB, MEXU); Heredia, Heredia, Distrito 6° San José de la Montaña, Cantón de Barva. Cerca de Porrosatí, 10°6'N, 84°7'W, 2000 m, 6 Jan 1991, ♂, flowers, *Hammel 18228* (CR, K, MO); **Heredia**: Distrito Varablanca, Vara Blanca de Sarapiquí, north slope of Central Cordillera, between Poás and Barba volcanoes, 10°11'2.85"N, 84°9'12"W, 1680 m, 1 Jan 1938, ♀, flowers, fruits, *Skutch 3393* (K, MO, US); Region between San Rafaél and the Río San Rafaél about 3 km east of Vara Blanca, 1800 m, 10°10'17.96"N, 84°8'21.84"W, 23 Feb 1976, ♀, flower buds, flowers, fruits, *Utley & Utley 4166* (DUKE, MO); Barva, No protegida, Cuenca del Tárcoles, entre Porrosatí y Sacramento, faldas del Volcán Barva, 10°5'22"N, 84°6'22"W, 1950 m, 24 Apr 2002, ♀, young fruits, *Hammel 22523* (CR); Barva, P.N. Braulio Carrillo, S slope of west forest, 1950 m, 16 Jan 1965, ♀, flower buds, flowers, *Hatheway 1275* (CR); Barva, Montaña, La Isla, Sacramento, 2500 m, ♂, old flowers, 13 Jan 1987, *Gómez-Laurito 11304* (CR, F); Barva, Cordillera Central, entre Porrosatí y Sacramento, faldas del Volcán Barva, 10°5'22"N, 84°6'22"W, 2000 m, 24 Jan 1996, ♂, flowers, *Hammel 20131* (CR, MO); cord. V. Central, faldas de V. Barva, Porrosatí, camino al volcán Barva, 10°5'45"N, 84°7'10"W, 2000 m, 12 Nov 1993, ♂, flowers, *Jiménez 1406* (CR, MO); faldas del Volcán Barva, entre Porrosatí y Sacramento, 2047 m, 10°5'38.20"N, 84°6'12.75"W, 6 Aug 2012, ♀, fruits, *Samain & Martínez 2012-041* (CR, GENT, IEB, MEXU); same data as preceding, 10°5'38.28"N, 84°6'15.92"W, 2035 m, 28 Jul 2012, inflorescence buds, *Samain & Martínez 2012-042* (CR, GENT, IEB, MEXU); Vara Blanca, terreno del restaurante Vara Blanca, 10°9'32.40"N, 84°9'20.28"W, 1842 m, 15 Jan 2013, ♂, inflorescence buds, flowers, *Samain & Martínez 2013-001* (CR, GENT, IEB, MEXU); faldas del Volcán Barva, entre Porrosatí y la entrada al parque, 10°7'17.65"N, 84°7'23.48"W, 2447 m, 18 Jan 2013, ♀, flowers, *Samain & Martínez 2013-012* (CR, GENT, IEB, MEXU); Heredia, 13.1 km N of Heredia via Rte. 114 (10.9 km N of Barva), 20 ft from road, 1500–2000 m, 15 May 1990, *Grantham & Parsons 0013-90* (CAS); **Limón**: Talamanca, Cordillera de Talamanca, Quebrada Kuisa, al paso del Sendero entre Ujarrás y San José Cabécar, 9°20'N, 83°13'55"W, 2100 m, 13 Mar 1993, ♀, fruits, *Fernández 653* (CR, MO); P.N. La Amistad, cuenca del Sixaola, sobre transecto 5, 9°21'18.1080"N, 83°13'29.9820"W, 1848 m, ♀, fruits, 25 Feb 2007, *Solano 4048* (PMA); Parque Internacional La Amistad, sendero Transtalamancana, entre Ujarras y San José Cabécar, alrededores del Río Lori, 9°21'18.6080"N, 83°13'19.2000"W, 1850–1900 m, 25 Feb 2007, ♀, fruits, *Rodríguez et al. 10829* (CR, PMA); Talamanca, P.N. La Amistad, Cuenca del Sixaola, transecto 5, 9°21'18.108"N, 83°13'29.892"W, 1848 m, 25 Feb 2007, ♀, fruits, *Solano 4046* (CR); Guápiles, Los Angeles, San Miguel, siguiendo el camino que lleva a San Gerardo de Oreamuno, volcán Irazú, 10°6'10"N, 83°50'45"W, 1300 m, 23 Feb 1990, ♂, flowers, *Herrera 3789 & Schik* (CR, MO); Cordillera de Talamanca, headwaters of the unnamed western branch of the Río Teribe, between the Río Sini and the continental divide at Cerro Bekom, 9°10'45"N, 83°3'30"W, 2500–2600 m, 21&27 Mar 1984, inflorescence bud, floral buds, *Davidse et al. 26158* (CR, MO); **Puntarenas**: Distrito Monteverde, Z. P. Arenal-Monteverde, Cuencas del Lagarto y Guacimal, Reserva Biológica Monteverde, a 1 km del inicio del Sendero principal, 10°19'17"N, 84°48'35"W, 1540 m, 1 Jun 1999, ♀, flower buds, flowers, *Hurtado 156* (CR, NY); on and near Continental Divide about 2 to 5 km. east and southeast of Monteverde, 10°18'9.94"N, 84°47'44.07"W, 1580–1700 m, 17–20 Mar 1973, ♀, fruits, *Burger 8613 & Gentry, Jr.* (DUKE, F, MO); Buenos Aires, P.N. La Amistad, Cuenca Térraba-Sierpe, sendero de Bekon a Tres Colinas, 9°9'42.965"N, 83°4'1.44"W, 2400–2500 m, 18 Apr 2002, ♂, flowers, *Alfaro 3978 et al.* (CR, MO); Z.P. Las Tablas, Cuenca Térraba-Sierpe, Zona Protectora Las Tablas, Surá, 7 km NO de Progreso, 8°54'17"N, 82°45'3"W, 2050 m, 18 Jan 1987, ♂, old flowers, *Navarro 579* (CR, MO); Puntarenas, Reserva Biológica Monteverde, Ojo de Agua, Finca de Leonel Hernández, lado pacífico de la reserva, 10°15'N, 84°46'W, 1600 m,18 Nov 1987, ♀, flower buds, flowers, *Haber 7811 & Bello* (CR, MO); Monteverde Community, Pacific slope, 1450 m, 10°20'N, 84°50'W, 13 Aug 1986, ♂, old flowers, *Haber ex Bello 5295* (MO); Reserva Biológica Monteverde, Pacific slope and continental divide, 10°18'N, 84°48'W, 1600 m, 17 Jun 1992, ♀, fruits, *Haber & Zuchowski 11210* (CR, MO); R.B. Monteverde, Cordilla de Tilarán, Monteverde, camino hacia Alto Cebadilla, 10°19'30"N, 84°51'W, 1300 m, 9 Feb 1994, ♀, fruits, *Ramírez 242 & Poveda* (CR, MO); Monteverde Cloud Forest Reserve, Cordillera de Tilarán, pacific slope of continental divide, Pantanoso trail, 1500–1620 m, 29 Dic 1984, ♀, fruits, *Pounds 379* (MO); Forest along trail between Las Alturas and Cotonsito, along the Río Cotón, 8°56'30"N, 82°48'W, 1400 m, 31 Aug 1983, ♂, flowers, *Davidse 24651* (CR, F, MO, MEXU); Foothills of the Cordillera de Talamanca, lower montane forest along the Río Bella Vista, NW of Las Alturas, 8°57'N, 82°51'W, 1450–1600 m, 30 Aug 1983, ♂, inflorescence buds, flowers, *Davidse 24300* (CR, MO); Monteverde Cloud forest Reserve, continental divide near La Ventana and elfin forest, 10°20'N, 84°50'W, 1600 m, 14 Dec 1985, ♂, inflorescence buds, flowers, *Haber ex Bello 3959* (CR, MO); same data as preceding, ♂, flowers, *Haber ex Bello 3969* (CR, MO); forest and forest edges on and around Wilson’s finca, 6 km south of San Vito de Java, about 4000 ft, 16 Aug 1967, floral buds, *Raven 21806* (F, MO); Puntarenas, Coto Brus, Z.P. Las Tablas, Cuenca Terraba-Sierpe, Sendero Sura, Zonas Protectora Las Tablas, 8°56'57"N, 82°44'37"W, 1920 m, 20 Dec 1997, ♂, old flowers, *Navarro 827* (CR, MO); Puntarenas, Puntarenas, Reserva Biológica Monteverde, Ojo de Agua, Finca de Leonel Hernández, lado pacífico de la reserva, 10°15'N, 84°46'W, 1600 m, 18 Nov 1987, floral buds, *Haber 7811* & Bello (CR, MO); Puntarenas, Bob Wilson´s Forest, San Vito de Java, near the Panamanian border, 22 Aug 1965, ♂, old flowers, *Walker 164* (F); Puntarenas, near Bob Wilson’s finca at San Vito Vine, 20 Aug 1965, ♂, old flowers, *Croat 1067* (MO); Foothills of the Cordillera de Talamanca, lower montane forest along the Río Bella Vista, NW of Las Alturas, 8°57'N, 82°51'W, 1450–1600 m, 30 Aug 1983, floral buds, *Davidse 24281* (MO); Monte Verde, 1500 m, 27 Dec 1984, ♂, inflorescence buds, flower buds, flowers, *de Nevers & Charnley 4427* (MO, PMA); **San José**: Desamparados, Distrito 2° San Miguel, Altos de Tablazo, about 5 km SE of Higuito on Calle Tablazo or about 12 km SE of Desamperados, 9°49'54.82"N, 84°2'28.28"W, 1800–1900 m, 30 Jan 1976, ♂, inflorescence bud, flowers, *Utley & Utley 3838* (DUKE, F); Distrito 8° San Cristobal, Cordillera de Talamanca. 4 km. north of El Empalme, 9°44'45.61"N, 83°58'36.99"W, 2070 m, 3 Mar 1986 ♂, flowers, *Almeda & Anderson 5298* (CAS); Distrito 9° Rosario, about 12 km. S of Higuito on the slopes of Altos Tablazo, 9°49'53.39"N, 84°2'26.69"W, 1800 m, 20 Feb 1978, ♂, flower buds, flowers, *Almeda 3757 & Nakai* (CR, CAS); Dota, Distrito 3° Copey, near Finca La Cima, above Los Lotes, North of El Copey, 9°40'15.4"N, 83°54'48.85"W, 2100–2400 m, 21–22 Dec 1925, ♀, flower buds, flowers, *Standley 42569* (US); Pérez Zeledón, Distrito 1° San Isidro de El General, roads above San Isidro de Coronado, 10°0'6.90"N, 84°0'21.38"W, 1400–1600 m, 1 Dec 1937–1 Jan 1938, ♀, flowers, *Allen 534* (F, GH); Vasquez de Coronado, Distrito 3° Jesús, La Hondura, 10°3'44.72"N, 83°58'55.21"W, 1300–1700 m, 16 Mar 1924, ♀, fruits, *Standley 37781* (US); Lisière des forets a L´Alto de la Palma, 10°2'55.35"N, 83°59'22.02"W, 1542 m, 30 Aug 1898, ♀, fruits, *Tonduz 12507* (US); La Palma, 10°2'55.71"N, 83°59'18.93"W, 1600 m, 3 Feb 1924, ♀, fruits, *Standley 33163* (US); Río Claro valley (Río La Hondura drainage) below La Palma northeast of San Jerónimo, 10°3'N, 83°58'W, 19 Nov 1969, 1000–1200 m, inflorescence buds, *Burger 6290 & Liesner* (CR, F, GB, NY); Woods above Río Hondura, 10°3'30"N, 83°58'28.86"W, 1150 m, 5 Dec 1971, ♀, fruits, *Lent 2272* (COL, CR, F, MEXU); heavily wooded slope leading from Alto La Palma to Bajo la Hondura about 12 km NNE of San Vicente de Moravia in Route 220 in the saddle between Irazú and Barba, 10°3'19.33"N, 83°59'10.46"W, 1200 m, 19 May 1971, ♀, fruits, *Wilbur 14687* (DUKE); Desamparados, Distrito 2° San Miguel, Altos de Tablazo, about 5 km SE of Higuito on Calle Tablazo or about 12 km SE of Desamparados, 9°49'54.82"N, 84°2'28.28"W, 1800–1900 m, 30 Jan 1976, ♀, fruits, *Utley & Utley 3844* (DUKE, F, MO); Perez Zeledon, Distrito, 4° Rivas, P. I. La Amistad, Cordillera de Talamanca, Entre las Nacientes de las quebradas Barranca y Río Blanco, Finca San Carlos, 9°31'47"N, 83°35'30"W, 05 Apr 1995, 2350 m, ♀, fruits, *Aguilar 3816 & Garrote* (CR, F, K, NY); Dota, San Gerardo de Dota, 2291 m, 9°34'18.56"N, 83°48'4.46"W, 24 Jan 2013, ♂, old flowers, *Samain & Martínez 2013-035* (CR, GENT, IEB, MEXU); Escazú, San Antonio, Z.P. Cerros de Escazú, falda SE del Alto Hierbabuena, 9°50'30"N, 84°7'35"W, 2100 m, 15 Oct 1992, ♀, flowers, fruits, *Morales 767* (CR, F, MO); Perez Zeledon, al lado del camino a San Gerardo de Dota, 4.22 km al SO del crucero con la Carretera Interamericana San Isidro de El General–Cartago, 9°33'27.20"N, 83°48'14.31"W, 2299 m, 11 Aug 2012, sterile, *Samain & Martínez 2012-068bis* (CR, GENT, IEB, MEXU); Turrubares, San Luis, Z.P. Cerros Turrubares, faldas del Cerro Bares, 9°47'30"N, 84°28'30"W, 1600 m, 6 Nov 1990, ♀, fruits, *Jiménez 919 et al.* (CR, F, MO); Bajo de Hondura, P.N. Braulio Carrillo, 10°4'N, 83°58'W, 1100–1200 m, 23 Jan 1983, ♂, old flowers, *Davidse 23195* (CR, MO); along Ca-2 on western ascent of Cerro de la Muerte, north of turnoff for road 222, 2000 m, 27 Feb 1976, ♂, inflorescence buds, flowers, *Croat 32829* (MO); San José, Sendero del camino viejo, hacia el Río La Hondura, P.N. Braulio Carrillo, 1250 m, 6–9 Dec 1983, ♂, old flowers, *Zamora & Carlson 418* (MO); San José, Aserrí, Cerros de Escazú, Tarbaca, bosque secundario en las cabeceras de la quebrada Saurez, 9°50'15"N, 84°7'10"W, 1900–2000 m, 6 Nov 1993, ♀, young fruits, *Morales 1979* (CR); San José, Vasquez de Coronado, R.F. Cordillera Volcánica Central, Cuenca del Sarapiquí, Cascajal de Coronado, calle Monserrat, 10°1'4"N, 83°56'31"W, 1700–1800 m, 6 Jul 2000, ♂, old flowers, *Vargas 459* (CR); San José, Moravia, No protegida, cuenca del Sarapiquí, paso de la Palma, de las Lecherías, 10°2'50"N, 83°59'20"W, 1500 m, 8 Nov 2003, floral buds, *Morales 10053* (CR); San José, Pérez Zeledón, Cordillera de Talamanca, Las Nubes de Santa Elena, tajo a orilla de la carretera, 9°23'30"N, 83°35'50"W, 1210 m, 7 Aug 1995, ♀, fruits, *Mora 245* (CR); San José, Parque Nal. Braulio Carrillo, camino entre El Bajo de la Hondura y La Montura, ca. 1200 m, 1 May 1982, ♂, old flowers, *Gómez-Laurito 8408* (CR, F); San José, Terrazú, San Lorenzo, R.F. Los Santos, fila San Isidro, 9°34'20"N, 84°4'10"W, 1350 m, 5 Nov 1997, ♂, flowers, *Valverde 384* (CR); San José, Dota, Santa María, Cerca de Palmitera de Dota, 1700 m, 20 Dec 1963, ♂, flowers, *Jiménez 1466* (CR, F); San José, Dota, Copey, R.F. Los Santos, San Gerardo de Dota, orillas del río Savegre, 2200 m, 26 Nov 1985, ♂, old flowers, *Soto s.n.* (CR); San José, Pérez Zeledón, Páramo, R.F. Los Santos, Lira, Estribaciones Noroeste de Cerro Lira, 9°31'30"N, 83°51'40"W, 1800 m, 8 Dec 1994, ♂, old flowers, *Martén 673 & Herrera* (CR, F); San José, P.N. Braulio Carrillo, Sendero del Camino Viejo, hacia El Río La Hondura, 1250 m, 9 Dec 1983, ♂, old flowers, *Zamora 417* (CR); without locality, ♂, old flowers, *Warcewicz 27368* (MO, photo from Delessert Herbarium).

**Panama. Bocas del Toro**: Cordillera de Talamanca, headwaters of the Río Culubre, 6 airline km NW of the peak of Cerro Echandi on the Costa Rican–Panamian international border, 09°05'00"N, 82°50'30"W, 2450–2600 m, 2–3 Mar 1984, ♀, fruits, *Davidse et al. 25254* (CR, MEXU, MO, PMA); E branch, headwater of Colubre River, 2300–2500 m, 2 Mar 1984, inflorescence bud, *Gómez et al. 22303* (MO); La Pata del Cedro, ridgetop, 09°04.270'N, 82°44.174'W, 1750 m, 11 Mar 2004, ♀, fruits, *Monro & Alfaro 4315* (CR, MO, PMA); **Chiriquí**: 1.4 mi S of Cerro Punta, 1850 m, 28 Dec 1963, ♀, fruits, *Graham 297* (MICH); along road and in pasturelands and stream banks above Las Nubes, NW of Cerro Punta, 1900–2100 m, 26 May 1973, ♀, fruits, *Luteyn 3797* (DUKE, MICH); Parque Internacional La Amistad, 100 m al SSW de la oficina, 8°53'36.3"N, 82°36'58.1"W, 2207 m, 10 May 2019, ♂, inflorescence buds, flowers, *Samain et al. 2019-003* (IEB, MEXU, PMA); Parque Internacional La Amistad, sendero El Retoño, 190 m al WNW de la oficina, 8°53'39"N, 82°37'0.53"W, 2227 m, 10 May 2019, ♀, flowers, *Samain et al. 2019-006* (IEB, MEXU, PMA); along trail between N fork of Río Palo Alto and Cerro Pate Macho, ca 6 km NE of Boquete, 8°48'N, 82°23.5"W, 1600–1700 m, 6 Feb 1986, ♂, old flowers, *Grayum et al. 6370* (MO); Alto Pineda, end of road, right turn just before cooperativa entrance to Cerro Punta, 8400 ft, 11 Apr 1979, ♂, inflorescence axes, old flowers, *Hammel et al. 6976* (MO); District Boquete, Alto Quiel, Finca Lerida, 05 Nov 1994, *Quiroz 166 & Garrido* (F, NY, PMA) (old inflorescence axes, flower buds); District Boquete, Bajo Chorro, 6000 ft, 7 Jan 1938, ♀, fruits, *Davidson 74* (F, GH, MO, UC, US); edge of forested slope above Cerro Punta toward Bajo Grande in Quebrado Bajo Grande, about 6500 ft, 14 Jan 1970, ♀, flowers, fruits, *Wilbur 10923 et al.* (DUKE, F, MICH, MO); Jurutungo, de la Fca. Los Quetzales, luego hacia la der. para luego bajar por el Río, 8°54'N, 82°43'W, ca. 1900 m, 24 Sep 1996, ♀, fruits, *Aranda 3251 et al.* (DUKE, MO, NY, PMA); Las Nubes, 5 km NW of Cerro Punta, 6000–6500 ft, 19 Jul 1975, ♂, flowers, *Mori & Bolten 7248* (MO); Monte Azul, 1.4 mi N of Entre Ríos on E slopes of Cerro Punta, 3 mi by road from town to Cerro Punta, 2250 m, 22 Nov 1979, ♀, flower buds, *Antonio 2693* (MEXU, MO, PMA); pastured area and bordering woods NE of Cerro Punta about 2.5 km, about 7200 ft, 29 May 1972, ♀, fruits, *Wilbur et al. 17149* (DUKE, MICH); road to Cerro Punta National Park from Alto Quiel and Boquete, 8°51'N, 82°29'W, c. 1850 m, inflorescence buds, 16 Jan 1986, *McPherson 8031* (MEXU, MO, PMA); trail up Cerro Pate Macho, 08°50'N, 82°25'W, 1500–1900 m, 7 Jan 1983, ♀, fruits, *Stein et al. 1215* (MEXU, MO); Valley of the upper Río Chiriquí Viejo, 18 Jan 1938, ♀, fruits, *White & White 90a* (MO); Vicinity of “New Switzerland”, central valley of Río Chiriquí Viejo, 1800–2000 m, 6–14 Jan 1939, ♂, flowers, *Allen 1400* (UC, US); 7 km northwest of Cerro Punta, Las Nubes region, 7200 ft, 11 Feb. 1978, floral buds, *Hammel 1428* (MO, PMA); vicinity of Fortuna, 8°45'N, 82°15'W, ca. 1250 m, 28 Apr 1986, ♀, fruits, *McPherson 9115* (MEXU, MO); Chiriquí, Boquete, Bajo Mono, 1600 m, Jan 1979, ♀, fruits, *Beliz 463* (PMA); Boquete, Sendero a Culebra, 1800 m, 18 Jan 2004, sex not visible because of insect damage, *Cáceres et al. 1836* (UCH); vicinity of Bajo Mona and Quebrada Chiquero, 1500 m, 18 Jul 1940, ♀, fruits, *Woodson & Schery 511* (GH, MO); distrito San Felix, Comarca Gnöbe Buglé, km 0 al 10 entre Hato Chami y Hato Ratón, ± 27–37 km al N de la 8°29'41"N, 81°46'16"W, 1386–1576 m, 20 May 2001, ♀, fruits, *Galdames et al. 4691* (F, PMA); Reserva Forestal Fortuna, Sendero La Casa Rosada, bosque cercano al Río Hornito, 8°42'00"N, 82°13'26"W, 1147 m, 31 Jan 2013, ♂, old flowers, *Ortiz 1172 et al.* (PMA). Without locality, either in Costa Rica or Panama (“Costa Rica et Veragua “), without date, ♂, flowers, *Warszewicz 1* (G).

#### 5. Hydrangea panamensis

Taxon classificationPlantaeCornaleHydrangeaceae

Standl., J. Wash. Acad. Sci. 17(1): 10. 1927.

3BC1AECF-02F8-52FF-9F70-5DDA0330A6E0

Figures 8 , 9

##### Type.

Panama. Colón, along Río Fato, 10–100 m, floral buds, *H.F. Pittier 3919* (holotype: US! [00097000], isotypes: A!, BM! [BM000028808], C!, F! [V0066620F], GH! [00042780], K! [K000486137], NY! [00007170]) floral buds.

##### Description.

**Figure 9.**
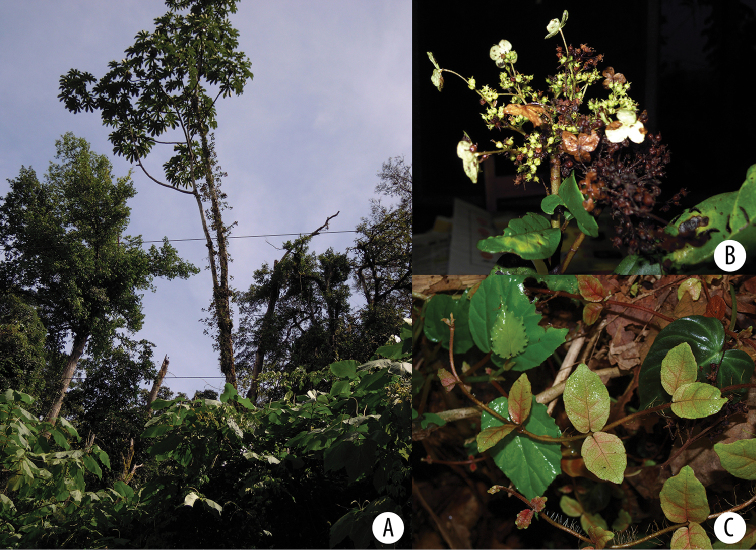
*Hydrangea panamensis***A** plant growing along the stem of a *Cecropia***B** infructescence with young and mature fruits, and enlarged marginal flowers. *Hydrangea peruviana***C** stolons with adventitious roots and decussate leaves. **A, B** field images of collection *Samain & Martínez 2012-063***C** field image of collection *Granados Mendoza et al. 2012-112*.

Root-climbing liana of up to 20 m high, never reaching above the lower branches of its host tree canopy (Fig. 9A), sometimes bending downwards, functionally dioecious; ***leaves*** decussate, petiole sulcate adaxially, terete abaxially, color reddish brown, densely pubescent with caducous, appressed, reddish, stellate hairs, 1–2 cm long, leaving a nearly triangular scar on the branch when leaves fall; lamina obovate to slightly elliptic, 5–12 cm long, 3–6 cm broad, base cuneate to rounded, slightly asymmetric, apex rounded with a very small acumen, leaf margin very slightly glandular-dentate, slightly revolute, venation brochidodromous, veins 4–6 pairs, adaxial leaf side with midvein and primary veins marked, primary veins join to form submarginal vein, pubescent with caducous, appressed, stellate hairs, the basal stalk reddish, the rest whitish, opaque pale green, abaxially with protruding midvein, primary veins alternately protruding and marked, secondary veins marked, regularly with concave cleavages on the junction of primary and secondary veins, opaque pale reddish brown, densely pubescent with appressed, stellate hairs, the basal stalk reddish, the rest whitish, acarodomatia very rare, 0–2 per leaf, consisting of a simple cavity in the axil of the midvein and primary veins; ***inflorescence axis*** pubescent with appressed, small, yellow-reddish, stellate hairs, shedding in older specimens, 2–10 cm long, quadrangular, with 1 decussate leaf and 1 kataphyll pair below the inflorescence, rapidly deciduous, ***apex of the floral axis*** woody, quadrangular, elongated bract scars visible, thickening at the top, 2–3 mm broad, 1–1.5 mm high in functionally female plants, 2–3 mm broad, 1 mm high in functionally male plants, ***inflorescence bracts*** cucullate, membranous, densely pubescent with reddish stellate hairs, veins palmate, bracts increasing in size, lowermost bract 1.5 cm large, 1.1 cm broad, higher bracts up to 2 cm large, 1.6 cm broad, ***inflorescences*** lateral, decussate, 1–3 pairs of inflorescences per flowering branch, flowering branch only continues growing very rapidly during inflorescence development, with up to 7 leaf pairs above the inflorescences, giving a very homogeneous aspect, inflorescence axes with basal lignified parts of inflorescences of previous years not observed, leaves or kataphylls present at the base of the inflorescence, inflorescence umbellate (Fig. 9B), buds up to 2 cm broad and 1.5 cm high before opening, in flowering stage 3–10 cm diameter, 1.5–5 cm high, with 5–8 main axes in functionally male plants, 5–8 main axes in functionally female plants, partial inflorescences racemoid, secondary and tertiary inflorescence axes with yellowish white stellate hairs, pubescence slightly decreasing towards flower insertion; ***enlarged marginal flowers*** always present (Fig. 9B), terminally placed in a raceme, sepals white, yellow or pink, fertile or reduced, when fertile stamens 8, pistils 2, fruit developing, when reduced only sepals developed, central part of the flower amorph, 0.5–0.7 cm diameter when flowering, 1.5–2 cm diameter with mature fruits, pedicel 1.2 cm long when flowering, 1.5–2 cm long with pedicel 1.5–2 cm long with mature fruits; ***flower pedicel of reduced flowers*** 0.5–2 mm long in functionally male flowers, 0.5–2 mm long in functionally female flowers, ***receptacle*** broadly triangular in functionally male flowers, semiglobose in functionally female flowers, ***ovary*** inferior, ***calyx lobes*** 4, broadly triangular, 0.2–0.3 mm long, ***petals*** 4, white, valvate, cucullate, membranous, 1.5–2 mm long, 1.5–1.8 mm broad; ***functionally male flowers***: hypanthium 1.2–1.8 mm diameter, 0.8–1 mm high, stamens 8, well-developed, filaments 1 mm long, anthers 0.5 mm long, 0.2 mm broad, pistils 2, reduced, 0.1–0.2 mm long, stigmas not penicellate; ***functionally female flowers***: hypanthium 1.5–1.6 mm diameter, 1–1.5 mm high, stamens 8, reduced, filaments 0.5 mm long, anthers 0.1 mm long, 0.1 mm broad, translucent, pistils 2, 1 mm long, enlarging up to 1.8 mm during fruit maturation, stigmas apically clavate and shortly penicellate; ***fruit*** a semiglobose capsule, apically with a conspicuous, slightly revolute border, 6–8 well marked ribs, dark reddish brown, 1.5 mm high, 3–3.5 mm broad above, 2 mm diameter, opening between the two pistils to release seeds, seeds not seen.

##### Distribution.

This species is known from Costa Rica and Panama (Fig. 8).

##### Habitat.

*Hydrangea panamensis* grows in tropical rainforest between 200 and 1000 m elevation. It usually occurs near water streams at lower elevations. Of all the species of the present study, this is the one that grows at the lowest elevation.

##### Phenology.

*Hydrangea panamensis* has been collected with flowers and fruits between June and September.

##### Notes.

Not to be considered a synonym of *H. peruviana* from which it can be distinguished by the rounded leaf apex with a very small acumen and the very few acarodomatia (0–2/leaf) on the abaxial leaf side vs. the acute to acuminate, rarely mucronate leaf apex and the many acarodomatia (present in both axils of midvein and primary veins and those of primary and secondary veins) of *H. peruviana*. Moreover, their known distribution areas are far away from each other, with *H. panamensis* only growing in Central America and *H. peruviana* being endemic to Ecuador.

The label of the specimen *Gentry 5569B* mentions that the flowers are red, whereas the label of the specimen *Mori & Bolten 7674* says “summit of ovary of fertile flowers reddish-pink”. It seems there is some color variation in this species in both the marginal and reduced flowers. Both flower types are generally white, and this is also the color we have observed in the field ourselves.

According to our molecular study, *Hydrangea panamensis* is closely related to *H. peruviana* (Granados Mendoza et al. unpublished results).

##### Preliminary conservation status.

Although this species has an EOO of about 50,123 km^2^, it is Endangered according to the IUCN categories and criteria (IUCN 2012), with an AAO of 60 km^2^, as well as an extensive reduction in both EOO and AOO as a consequence of habitat destruction and fragmentation.

##### Additional specimens examined.

**Costa Rica. Puntarenas**: Golfito, Playa Cacao, Cuenca Superior de Quebrada Nazareno, 8°37'50"N, 83°11'30"W, 200 m, 11 Jun 1994, ♂, flowers, *Herrera & Rivera 7152* (CR, F, K, MO); Golfito–La Gamba (km 37), gap by the waterfall – Sendero Ozelot near the Rainforest Lodge, 8°41'N, 83°13'W, 70–300 m, 20 Jun 1997, inflorescence buds, *Weissenhofer W105* (CR, WU); Golfito, Bosque de los Austriacos, Fila Gamba, Avilán woodcutter way, 8°41'N, 83°13'W, 250 m, 3 Jun 1997, ♂, old flowers, *Huber & Weissenhofer 747-3.6.97* (CR); Osa, al lado del camino Rincon–Bahía Drake, 2.23 km al ONO de Rancho Quemado, 8°41'35.39"N, 83°35'7.42"W, 237 m, 8 Aug 2012, ♀, fruits, *Samain & Martínez 2012-063* (CR, GENT, IEB, MEXU); Cerro Anguciana, cabeceras del Río Piedras Blancas, 4.96 km al NE de Piedras Blancas, 8°48'27.49"N, 83°12'18.05"W, 676 m, 9 Aug 2012, ♀, fruits, *Samain & Martínez 2012-064* (CR, GENT, IEB, MEXU).

**Panama. Darién**: Cana and vicinity, 2000–6500 ft, 7 Apr 1908–8 Jun 1908, old inflorescence branches, *Williams 775* (NY); **Coclé**: between Cerro Pilón and el Valle de Antón, 700–900 m, 15 May 1967, ♀, flowers, *Duke & Dwyer 13952* (MO); region north of El Valle de Anton, 1000 m, 27 Sep 1946, ♀, fruits, *Allen 3712* (E, GH, F, MO, P, UPS); alrededores de El Valle de Antón, Altos de La Mesa, 1.3 km al N de la bifurcación hacia Río Indio, 8°38'38” N, 80°06'51"W, 735 m, ♂, flowers, 13 Aug 2014, *Galdames et al. 7676* (PMA); Distrito La Pintada, cerca de las cabañas de ANAM, 8°40'05"N, 80°35'34"W, 800–900 m, 14 Aug 2007, ♀, fruits, *Hernández et al. 528* (PMA); **Panama**: Canal Zone, Barro Colorado Island, E. Of Wheeler 13, 18 Aug 1970, ♀, flowers, *Foster 1797* (DUKE, F, PMA, UC); Barro Colorado Island, forest 200 m E of Wheeler Trail 1300, 18 Aug 1970, ♀, fruits, *Croat 11850* (AAU, DUKE, E, F, MO); pipeline road, 14 Sep 1971, ♀, fruits, *Gentry 1792* (DUKE, F, GH, MO); Panamá, North of El Llano, 500–800 m, 25 Jul 1972, ♀, flowers, *Gentry 5569B* (MO); Cerro Campana, 45 km SW of Panama City on Inter-American Hwy, 8 Aug 1975, ♀, fruits, *Mori & Bolten 7674* (AAU, MO); Distrito de Capira, Parque Nacional Altos de Campana, Sendero de Interpretación, 8°40'54"N, 79°55'40"O, ca. 750 m, 23 Aug 1990, ♀, fruits, *Galdames 880* (PMA); Parque Nacional Altos de Campana, Sendero de interpretación, 1 km al este del campamento de los guardaparques de INRENARE, parcela 10-8, 8°40'N, 79°55'W, 800–900 m, 19 Aug 1993, ♂, flowers, *Correa & Montenegro 9780* (PMA, UCH).

#### 6. Hydrangea peruviana

Taxon classificationPlantaeCornaleHydrangeaceae

Moric. ex Ser., Prodr. [A.P. de Candolle] 4: 14. 1830.

3FE03029-B613-5ADB-A241-E6CE06102D2F

**Figure 10.**
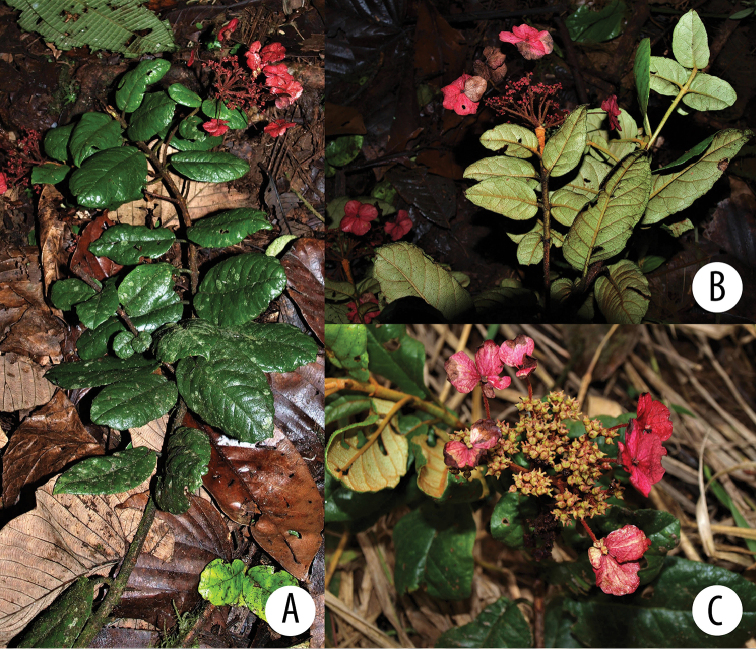
*Hydrangea peruviana***A** branch with inflorescence **B** branch with leaves seen abaxially and inflorescence **C** close up of infructescence with maturing fruits and enlarged marginal flowers. **A, B** field images of collection *Granados Mendoza et al. 2012-111***C** field image of collection *Granados Mendoza et al. 2012-112*.

**Figure 11.**
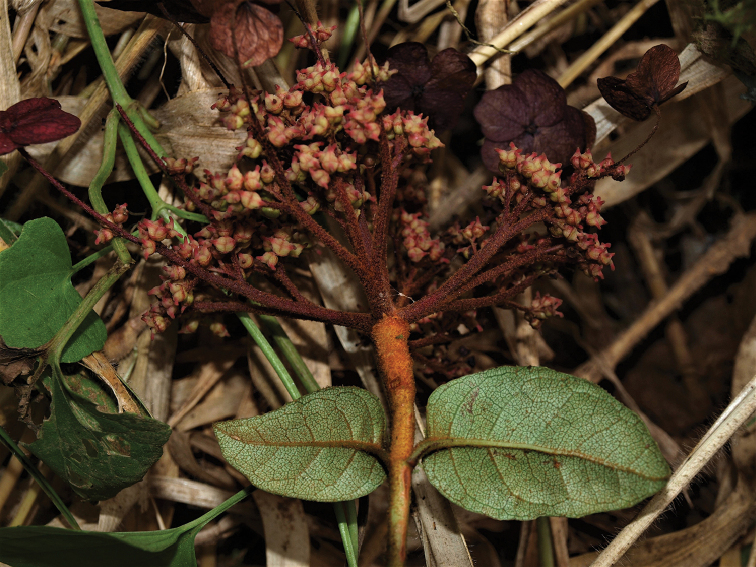
*Hydrangea peruviana*. Infructescence with young fruits and densely pubescent apex of the inflorescence axis. Field image of collection *Granados Mendoza et al. 2012-112*.

Figures 6 , 9 , 10 , 11

Cornidia peruviana (Moric. ex DC.) Small, North American Flora 22(2): 161. 1905.

##### Type.

Ecuador. “In Peruvia prope Huyaquaquil”, ♀, fruits, *J.A. Pavón s.n.* [*J.J. Tafalla s.n.*] (holotype: G! [G00301424], isotypes: F!, MA! [MA-811940])

##### Description.

Root-climbing liana of up to 30 m high, up to 20 cm diameter, functionally dioecious; ***leaves*** decussate, coriaceous, petiole sulcate adaxially, clasping its branch, color reddish brown, densely pubescent with partially caducous, reddish simple and stellate hairs, 0.5–2 cm long, leaving a semicircular scar on the branch when leaves shed; lamina very slightly spoon-shaped (Figs 9C, 10A, B), elliptic to slightly obovate, 7.5–12.8 cm long, 4.2–5.4 cm broad, base rounded to decurrent, sometimes asymmetric or very slightly cordate, apex acute to acuminate, rarely mucronate, leaf margin serrate to slightly dentate, venation brochidodromous, veins 6–10 pairs, adaxial leaf side with midvein and primary veins slightly protruding secondary veins marked, primary veins join to form submarginal vein, pubescent with small, stellate whitish pubescence, abaxially with protruding midvein and primary veins, sometimes with a few smaller less visible primary veins between the clearly visible primary veins, marked secondary veins, secondary and tertiary veins forming a reticulate network, connecting the primary veins, reddish brown, pubescent with small, stellate reddish hairs, especially on the veins, young leaves densely pubescent, acarodomatia numerous, present in axils of midvein and primary veins as well as axils of secondary veins, veins broadening the acarodomatia, consisting of a cavity, rarely with hairs; ***inflorescence axis*** densely pubescent with brownish stellate hairs (Fig. 11), 7–12 cm long, broadening towards the apex, with 3–4 opposite or decussate leaf or kataphyll pairs below the inflorescence, generally not deciduous, petiole 2–4 mm long, adaxially sulcate, lamina nearly orbicular to obovate, 1.3–4.2 cm long, 1.2–2.8 cm broad, densely pubescent, scars of 2 pairs of kataphylls present, ***apex of the inflorescence axis*** woody, cone-shaped, slightly quadrangular, elongated bract scars visible (Fig. 11), narrower at the top, 4–5 mm broad, 2–3 mm high in functionally female plants, male inflorescences not seen; ***inflorescence bracts*** not seen, ***inflorescences*** lateral, opposite, 1 pair of inflorescences per flowering branch, sometimes only one inflorescence developing (Fig. 10A,B), flowering branch continues growing vegetatively very rapidly during inflorescence development, including additional branching, with up to 5 leaf pairs above the inflorescences and below the first branch, with kataphylls opposite the branches, with dense reddish stellate hairs, inflorescence umbellate, buds not seen, in flowering stage 4.5–10 cm diameter, 4–7 cm high, 3–7 main axes in functionally female plants, partial inflorescences cymes, secondary and tertiary inflorescence axes with dense reddish stellate hairs; ***enlarged marginal flowers*** always present (Figs 10–11), terminally placed in a cyme, 1.5–2 cm diameter, sepals 1–4, sepals with marked veins, remnants of 2 pistils visible, further characters not observed in detail, pedicel 1.2–2.4 cm long; male flowers not seen, (0–)1–1.5(–2) mm long in functionally female flowers, ***receptacle*** semiglobose in functionally female flowers, ***ovary*** inferior, ***calyx lobes*** 4, triangular, papyraceous, 0.25 mm long, 0.25 mm broad, ***petals*** not seen; ***functionally female flowers***: hypanthium 1.5 mm broad, 1 mm high, 8 well-marked ribs, stamen scars visible but too small to detect a number, pistils 2, 0.2–0.3 mm long, enlarging up to 2–2.5 mm during fruit maturation, stigmas apically clavate and shortly penicellate; ***fruit*** a semiglobose capsule (Figs 10, 11C), apically with a revolute border, dark reddish brown, 1.5 mm high, 2 mm broad above, 3 mm diameter, opening between the two pistils to release seeds, seeds not seen.

##### Distribution.

*Hydrangea peruviana* is a rare species which is known from Ecuador only (Fig. 6). Apart from the type collection, it is only known of six collections since 1980, two of which were realized during the explorations in the framework of our revision of the Neotropical Hydrangeas. It was found in a primary mountain cloud forest flowering very high (about 30 m) in the tree canopy. The cloudy environment in combination with the height at which the specimens were flowering made them very difficult to spot, potentially being the reason why this species has been so rarely collected by botanists.

##### Habitat.

This species has been reported in rainforest and cloud forest at elevations between 682 and 1300 m.

##### Phenology.

This species has been collected with flowers and fruits in March and July. Only female plants have been observed. There are no collections known of male individuals.

##### Notes.

Since the revision by McClintock (1957), most of the species mentioned in this paper had been lumped in *H. peruviana*. Following the treatment of the genus *Hydrangea* for Ecuador by Freire-Fierro (2004), *H. oerstedii* was reduced to a variety of *H. peruviana*, consequently all species of the present study belonged to what was until recently considered as a species complex. However, based on our extensive study of herbarium specimens, including type material, and field observations, it became clear that *H. peruviana* is a very distinct taxon which can easily be recognized by the densely pubescent reddish brown leaves with an acute to acuminate, rarely mucronate, apex, a serrate to slightly dentate margin and many characteristically shaped acarodomatia.

In contrast to what might be expected because of its name, *H. peruviana* is not known from Peru, the type locality area of Guayaquil now being the second largest city of neighboring Ecuador, and this country´s main harbor. However, at the time of its collection in the late 18^th^ century, modern-day Peru and most of Spanish-ruled South America belonged to the Viceroyalty of Peru.

As mentioned by Macbride (1938), it is generally accepted that their collections in the area that correspond with present-day Ecuador were not realized by Spanish botanists Ruiz and Pavón, but by their collaborator Juan José Tafalla.

According to our molecular study, *Hydrangea peruviana* is closely related to *H. panamensis*, the two of them unrelated to the other species of this study (Granados Mendoza et al. unpublished results).

##### Preliminary conservation status.

Although this species has an EOO of about 13,515 km^2^, it is Endangered according to the IUCN categories and criteria (IUCN 2012), with an AAO of 20 km^2^, less than five locations, as well as an extensive reduction in both EOO and AOO because of habitat destruction and fragmentation.

##### Additional specimens examined.

**Ecuador. Carchi**: Camino Chical–Peñas Blancas–Tobar Donoso, colec. a 5 horas de camino, 1°0'N, 78°12'W, 6 Dec 1993, 1200 m, sterile, *Freire-Fierro 2616* (AAU, QCA); **Esmeraldas**: environs of Lita, on the Ibarra–San Lorenzo R.R., 550–650 m, 11 Jun 1978, ♀, fruits, *Madison et al. 5251* (F, QCA); **Pichincha**: km 87–84 old road Quito–Santo Domingo, 1200–1300 m, 21 Mar 1980, ♀, fruits, *Dodson 9733* (MO); Los Ríos, Road Patricia Pilar–Montañas de Ila, km 18, N side of Torre de Bijagual, below antenas, 00°38'S, 79°17'W, 620–680 m, 28 Feb 1993, ♀, fruits, *Øllgaard & Borchsenius 100686* (QCA, QCNE); **Santo Domingo de los Tsáchilas**: 5.3 airline Km SW of Corina Parral, 0°39'20.8"S, 79°17'27.7"W, 693 m, 11 Jul 2012, ♀, fruits, *Granados Mendoza et al. 2012-111* (GENT, IEB, MEXU, QCNE); same data as preceding, 0°39'21.5"S, 79°17'29.2"W, 682 m, 11 Jul 2012, ♀, fruits, *Granados Mendoza et al. 2012-112* (GENT, HOXA, IEB, MEXU, QCNE).

#### 7. Hydrangea schlimii

Taxon classificationPlantaeCornaleHydrangeaceae

Briq., Annuaire Conserv. Jard. Bot. Genève 20: 400. 1919.

28642B68-D16C-5AD9-82E3-C006045A0DEA

Figures 2 , 12

##### Type.

Colombia. Norte de Santander, Ocaña, without date, ♂, old flowers, *J.J. Linden 1139* (lectotype, designated by McClintock 1957, pg. 238 [as “holotype”]: G; isotypes: BR! [BR0000005119318], US! [00097002]).

##### Description.

The most complete description to date can be found in the treatment by Briquet (1919), pages 400–401.

##### Distribution.

This species is currently known from the type locality in Colombia only (Fig. 2).

##### Notes.

This species concerns a very distinct taxon with more enlarged “marginal” flowers than any other member of *Cornidia*. In fact, from the type specimen it seems that most flowers possess enlarged sepals. It is only known from its type collection made around 1850 and to our knowledge has not been collected since. It cannot be excluded that it concerns a local “mophead” mutation, but in the absence of recently collected material and as the leaf morphology of the type specimens cannot be matched with better-known species, we decide here to recognize this taxon as a distinct species, although we cannot present an amended description at this time, given that we only dispose of the type specimen, which already was given an excellent description by Briquet (1919).

As in the case of *H. durifolia*, exploring field work in forests around Ocaña in the Colombian department of Norte de Santander might lead to the rediscovery of this species, which will be helpful to define its taxonomic status.

McClintock (1957) cited as “holotype” the collection in G, which we have not been able to locate. Based on the many enlarged marginal flowers and the inflorescence leaf morphology, this species should not be considered a synonym of *H. oerstedii*.

The phylogenetic relationships of *Hydrangea schlimii* are yet unknown as there was no fresh material available for our molecular study (Granados Mendoza et al. unpublished results).

##### Preliminary conservation status.

We currently propose this species as Data Deficient (DD), as its taxonomic status in unclear, it is only known from the type locality, and no recent collections are available.

**Figure 12.**
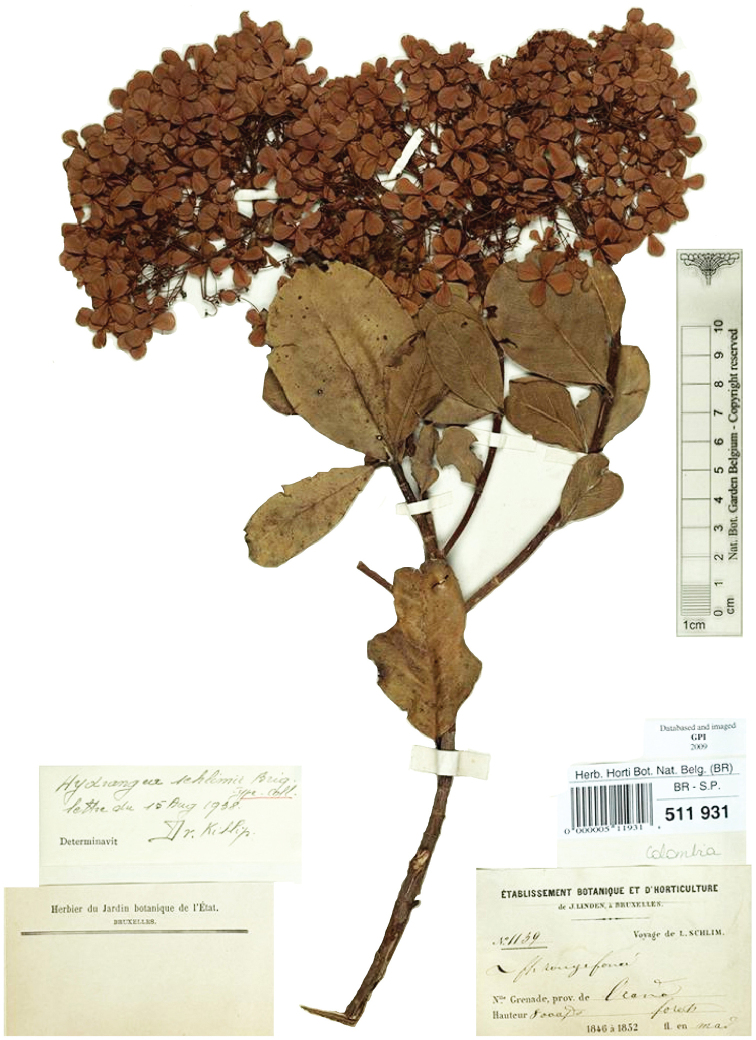
*Hydrangea schlimii*. Branch with inflorescences with many enlarged flowers. Image of specimen *Linden 1139* (BR).

#### 8. Hydrangea trianae

Taxon classificationPlantaeCornaleHydrangeaceae

Briq., Annuaire Conserv. Jard. Bot. Genève 20: 403. 1919.

BE4C15D3-218F-5201-8C13-0229F9D56841

Figures 13 , 14

H. lehmannii Engl., Nat. Pflanzenfam. (ed. 2) 18a: 207. 1930. Type. Colombia. Valle del Cauca: Cali, 4 Oct. 1900, ♀, fruits, *F.C. Lehmann 9074* (lectotype, designated by Freire-Fierro 2004, pg. 33, second-step designated here: K! [K000486136]; isolectotypes: B [destroyed, F neg. 4144], K! [K000486135])
Hydrangea platyphylla Briq., Annuaire Conserv. Jard. Bot. Genève 20: 401. 1919. Type. Colombia. Tolima: Mariquita, ♂, old flowers, *J.J. Linden 894* (lectotype, designated by McClintock 1957, pg. 238 [as “holotype”], second-step designated here: G! [G00439775, F neg. 27369]; isotypes: F [V0066625F]!, G! [G00439776, G00223929], GENT! [BR0000005119332, BR0000005119660] (photos of both on one sheet in COL!, GH!, K!, NY!), K! [K000486132]!

##### Type.

Colombia. Tolima: Quindío, ♂, flowers, *J.J. Triana s.n.* (holotype: G-DC n.v. (photo in GENT!), isotypes: BM!, BR [BR0000005119011, BR0000005119974, BR0000005119646]!., COL [COL000369449] !, F! [V0066627F], MEXU! [acc. # 14858], MICH!)

##### Description.

**Figure 13.**
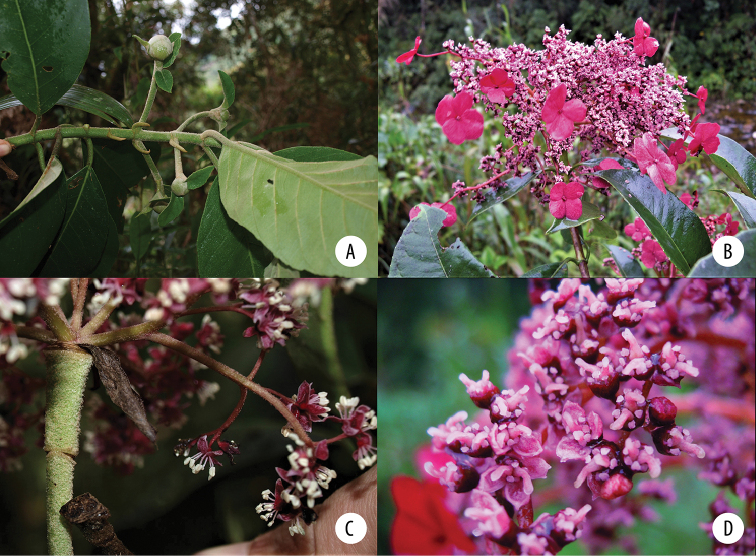
*Hydrangea trianae***A** branch with leaves seen abaxially and inflorescence buds **B** functionally female inflorescence with enlarged marginal flowers **C** basal portion of functionally male inflorescence with densely pubescent apex of the inflorescence axis, and a few open male flowers with large stamens **D** close-up of functionally female flowers with petals, reduced stamens and large pistils. **A, C** field images of collection *Samain et al. 2011-064***B, D** field images of collection *Samain et al. 2011-067*.

Root-climbing liana of up to 20 m high, functionally dioecious; ***free-growing branches*** many-ribbed, slightly angular, old branches quadrangular, with erect, white stellate hairs (Fig. 13A); ***leaves*** decussate, petiole terete, sometimes abaxially flattened, color dark green, sometimes drying black, pubescent with small, erect, whitish to yellowish stellate hairs, 1.5–4 cm long, leaving a semicircular scar on the branch when leaves shed; lamina spoon-shaped, obovate to elliptic, (slightly) asymmetric, coriaceous, 15–29 cm long, 8.5–18 cm broad, with varying length-width ratios, base cuneate, acute or decurrent, apex rounded to acuminate, leaf margin (slightly) dentate, venation brochidodromous, veins 8–9 pairs, adaxial leaf side with protruding midvein and primary veins, secondary veins slightly protruding, primary veins join to form submarginal vein, dark green, slightly pubescent with white stellate hairs, especially near the margin, abaxially with protruding veins, midvein notoriously protruding, opaque olive green, pubescent with stellate white hairs, primary veins branching towards the margin, ending in a submarginal vein very near the margin, secondary and tertiary veins forming a reticulate network, connecting the primary veins, acarodomatia numerous, consisting of an often small simple cavity in axils of midvein and primary veins, sometimes covered by a membrane; ***inflorescence axis*** in the axils of kataphylls of 1–1.4 cm long or fully grown leaves, pubescent with erect, white, stellate hairs (Fig. 13C), 6–25 cm long, many-ribbed, with 2 opposite rapidly shedding leaf or kataphyll pairs along the axis, petiole of the leaves 5–10 mm long, lamina obovate to lanceolate, (slightly) asymmetric, 2–7 cm long, 1.4–4.3 cm broad, margin glandular dentate, small, appressed, stellate, abaxially and adaxially with white hairs along the midveins, similar pubescence between the veins but not appressed and larger, veins 6 pairs, all protruding, in the case of kataphylls, these initially protecting the small inflorescence buds (Fig. 13A), followed by elongation of the axis occurring between the kataphylls and the inflorescence bud, ***apex of the inflorescence axis*** woody, quadrangular, elongated bract scars visible, thickening at the top, 7–8 mm broad, 2 mm high in functionally female plants, 6–7 mm broad, 2–3 mm high in functionally male plants, inflorescence bracts cucullate, dark green, coriaceous, abaxially (densely) pubescent with small, erect, whitish and yellowish stellate hairs, ***inflorescences*** lateral, opposite, 1–2 pairs of inflorescences per flowering branch, flowering branch continues growing rapidly during inflorescence development, with up to three decussate leaf pairs above the inflorescences, with appressed, whitish and yellowish stellate hairs, dense when young, rapidly shedding, inflorescence axes with basal lignified parts of inflorescences of previous years visible in well-collected specimens, allowing to observe growth and flower periodicity, these rests 2.5–5 cm apart, 7 cm when inflorescence originate in the axils of the regular leaves, with none or one decussate leaf pair (or the scars of these leaves) in between, inflorescence umbellate, buds up to 4 cm broad and 4.5 cm high before opening, in flowering stage 7–19 cm diameter, 4–12 cm high, with 5–9 main axes in functionally male plants, 4–8 main axes in functionally female plants, partial inflorescences dichasia, secondary and tertiary inflorescence axes with whitish and/or reddish stellate hairs; ***enlarged marginal flowers*** generally present (but e.g. absent in *Samain et al. 2011-064*) (Fig. 13B), terminally placed in a cyme, 1.8–4 cm diameter, sepals with marked palmate veins, fully separated or fused at the base, sometimes one or two reduced or even absent, pistils very rudimentary, pedicel 1–3.5 cm long; ***flower pedicel of reduced flowers*** 1–2 mm long in functionally male flowers, 1–5 mm long in functionally female flowers, ***receptacle*** semiglobose in functionally male flowers, broadly semiglobose in functionally female flowers, ***ovary*** inferior, ***calyx lobes*** 4, triangular, translucid, 0.2–0.5 mm long, 0.6–1 mm broad, ***petals*** 4, adaxially red, abaxially purple with white margin, valvate, cucullate with a small mucron at the apex, coriaceous, 1.5–3 mm long, 1–1.5 mm broad; ***functionally male flowers*** (Fig. 13C): hypanthium 1.5–2 mm diameter, 1.5 mm high, stamens 8, well-developed, filaments 1.2–2 mm long, anthers 1–1.5 mm long, 0.5–1 mm broad, purple, pistils 2, very reduced, 0.5 mm long, stigmas not penicellate; ***functionally female flowers*** (Fig. 13D): hypanthium 1.2–2.2 mm diameter, 1.5 mm high, stamens 8, very reduced, filaments 0.2–0.5 mm long, anthers 0.2–0.4 mm long, 0.1–0.2 mm broad, pistils 2(–3), 1.5–2 mm long, broad at the base, narrower in the middle and broadening towards the apex, enlarging up to 3.2 mm during fruit maturation, stigmas apically clavate and shortly penicellate, 0.5 mm long; ***fruit*** a semiglobose capsule, apically with a conspicuous border, dark reddish brown, 2.5 mm high, 4–4.5 mm broad above, 3–4 mm diameter, the part around the pistils thickening upwards up to 1 mm during fruit maturation, opening between the two pistils to release seeds, seeds not seen.

##### Distribution.

**Figure 14.**
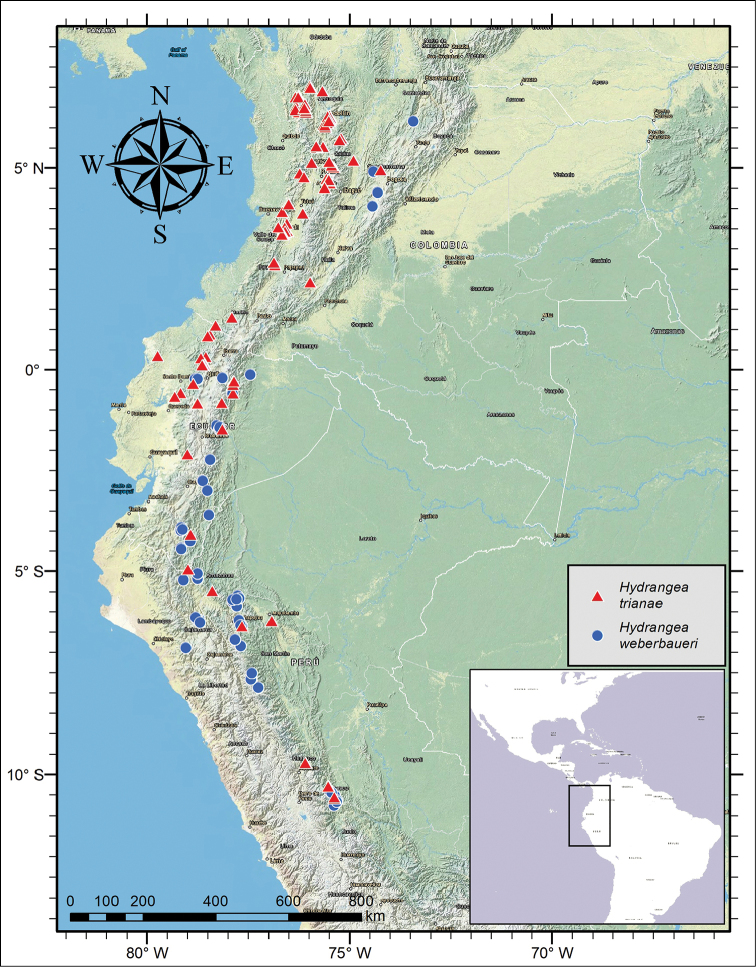
Distribution of *Hydrangea trianae* and *Hydrangea weberbaueri*.

*Hydrangea trianae* is a widespread species occurring in Colombia, Ecuador and Peru (Fig. 14).

##### Habitat.

*Hydrangea trianae* is known from cloud forest and remnants of this vegetation type at elevation between 400–3680 m. Moreover, it has been noted to occur in disturbed or late secondary forests.

##### Phenology.

This species has been collected with flowers and fruits throughout the year.

##### Notes.

*Hydrangea trianae* should not be considered a synonym of *H. peruviana*, from which it can be distinguished by the notoriously coriaceous leaves, the abaxial reticulate network of secondary and tertiary veins connecting the primary ones, and the abaxial white pubescence.

*Hydrangea lehmannii* was synonymized with *H. peruviana* by McClintock (1957), whereas she considered *H. platyphylla* as a synonym of *H. oerstedii*.

With respect to the type material of *Hydrangea lehmannii*, McClintock (1957) cited the K material as an isotype, albeit without designating a specific sheet, and this was also cited by Freire-Fierro (2004).

With respect to the type material of *H. platyphylla*, McClintock (1957) cited G material as holotype, which effectively lectotypified the name. However, there are two specimens of this collection in G, therefore requiring second-step lectotypification, which is done here.

One of the F specimens (V0066626F), which supposedly is an isotype of *H. platyphylla*, does not belong to this species based on leaf venation, probably due to a labeling error; however, it is not possible to propose any other identification as the material is very limited.

Briquet (1919) distinguished *H. trianae* and *H. platyphylla* based on leaf shape and venation, as well as size of cymes, sepals of enlarged marginal flowers and styles, which can be contributed to the sex of the single collections of both species he used for his descriptions, the specimens of *H. trianae* being functionally female and the ones of *H. platyphylla* which are functionally male.

The specimen of *Triana 4668* in NY does not correspond with *H. trianae* or any of the other species treated here, in contrast with the specimen with the same number in COL. Triana’s numbers do not correspond to field collection numbers as currently used, but instead are generic numbers based on the Endlicher system (Kirkbride 1982; Aymard 2017).

According to our molecular study, *Hydrangea trianae* is closely related to *H. goudotii* (Granados Mendoza et al. unpublished results).

##### Preliminary conservation status.

Although this species has an EOO of nearly 726,300 km^2^, it is Endangered (B2abiii) according to the IUCN categories and criteria (IUCN 2012), with an AOO of 372 km^2^, as well as an extensive reduction in both EOO and AOO as a consequence of habitat destruction and fragmentation.

##### Additional specimens examined.

**Colombia. Antioquia**: Argelia, km 17 of road Sonsón–Argelia (2 km past fork in road to Nariño), 2160 m, 3 Oct 1987, ♀, fruits, *Zarucchi et al. 6164* (HUA, MO); Frontino, Correg. Nutibara, Nutibara–La Blanquita road, region of Murrí, Alto de Cuevas, 6°45'N, 76°20'W, 1700–1800 m, 19 Apr 1988, ♂, old flowers, *Luteyn et al. 12047* (HUA, K, MO); Urrao, Parque Nacional Natural las Orquideas, vereda Calles, 06°32'N, 76°19'W, 1350–1450 m, 5 Dec 1993, ♀, inflorescence buds, flowers, *Pipoly et al. 17737* (MO); Jardín, km 20 of road Jardín–Riosucio (dpto. Caldas), ca 15 km SSE of Jardín, Alto de Ventanas, 29 Oct 1988, 2700–2790 m, ♂, flower buds, *Zarucchi et al. 6962* (HUA, MO); road between Jardín and Río Sucio, ca 9 km from Jardín, 2300–2400 m, 29 Jan 1989, ♂, flowers, *MacDougal & Roldán 3581* (HUA, MO); Frontino, region of Murrí, road between Nutibara and La Blanquita, 19.2 km from centro de Nutibara, 1560 m, 11 Feb 1989, ♂, old flowers, *MacDougal et al. 3929* (CR, HUA, MO); Frontino, Corregimiento Nutibara, Región Murí, márgen de río, 1500 m, 11 Jul 1986, ♀, old inflorescences, *Acevedo et al. 1260* (HUA, NY, US); along road between Caramanta and Supia (Risaralda), ca 4 mi S of Caramanta, 2030 m, 27 Jan 1990, ♀, flowers, *Croat 70038* (MO); Medellín y Guarne, Parque Ecológico Piedras Blancas, laguna de Guarne, 2350 m, 26 Aug 1995, ♂, old inflorescences, *Roldán & Medina 2385* (HUA, MO); Parque Ecológico Piedras Blancas, sector Lajas, 2350 m, 23 Sep 1995, ♂, flowers, *Fonnegra et al. 5643* (HUA, MO); Parque Nacional Natural “Las Orquideas”, sector Calles, margen derecha del río Calles, 1250 m, 30 May 1988, ♀, fruits, *Cogollo et al. 3087* (COL, MO); Urrao, Corregimiento Encarnación, sitio El Río, 1 hora de camino del Páramo de Frontino, 3120 m, 7 Apr 1989, inflorescence buds, *Callejas et al. 7774* (HUA, NY); same data as preceding, 3000–3150 m, 8 Apr 1989, ♀, flower buds, flowers, *Callejas et al. 7835* (HUA, MO); Envigado, Vereda “El Escobero”, Costa Occidental Cerro “San Luis”, 2100–2250 m, 15 May 1996, ♂, inflorescence bud, flower buds, flowers, *Correa & Carona 1073* (COL, HUA, MO); cerca de San José de San Andrés, 1 May 1948, inflorescence buds, floral buds, *Correa & Velasquez 45* (US); bosque bajo de la cumbre cerca de Santa Elena, camino entre Medellín y Río Negro, 2300–2500 m, 3 Aug 1945, ♀, fruits, *Medina 239* (US); municipio de Caldas, Alto de San Miguel, 2200 m, 8 Jul 1996, ♂, flowers, *Monsalve 38* (F); municipio de Peque, 7°2'6.7"N, 75°58'27"W, 2500 m, 19 Nov 1995, ♀, fruits, *Benítez et al. 584* (COL); municipio de Peque, vereda Romeral, 6°59'18.4"N, 75°58'02.5"W, 2650 m, 16 Nov 1995, floral buds, *Benítez et al. 452* (COL); municipio Caldas, Reserva “Alto de San Miguel”, cuenca alta del río Medellín, 6°5'N, 75°38'W, 2100 m, ♂, old flowers, *Roldán et al. 2516* (HUA); municipio de Caldas, vereda La Corrala, Finca “La Zarza”, Alto del Gallinazo, ca. 2600 m, 4 Feb 1986, inflorescence buds, *Albert de Escobar y Stein 6238* (HUA); municipio de Urrao, Finca La Clara, cerca del río Mina y La Clara, 2340 m, 6 Dec 1984, ♀, fruits, *Orozco et al. 1782* (COL); camino de Urrao al páramo de Frontino, 2400 m, 19 May 1985, ♂, flower buds, flowers, *Cárdenas 128* (HUA); Argelia, vereda La Vibora, antiguo camino de Sonsón a Argelia, 2200–2700 m, 28 Aug 1989, ♂, flowers, *Hoyos y Avendaño 1300* (HUA); municipio Ríonegro, vereda Yarumales, 20–30 km SE de Medellín en la vía a Rionegro, 6°15'N, 75°28'W, 2140–2410 m, 12 Nov 1990, ♀, fruits, *Callejas & Roldán 9628* (HUA); municipio de Urrao, Páramo de Frontino, El Río, 3020 m, 6 Jan 1985, ♀, fruits, *Londoño et al. 669* (HUA); Páramo de Frontino, Urrao – zona situada entre el 15 y la Esperanza, 2980–3680 m, 18 May 1985, ♀, fruits, *Rentería 4082* (HUA): Medellín, corregimiento de Santa Elena, Sector de Piedras Blancas, 6°17'N, 75°32'W, 2400 m, May 1998, ♂, flowers, *Cardona y Alzate 551* (HUA); Medellín, corregimiento de Santa Elena, Reserva Natural Montevivo, sector Casapalo, 6°12'48"N, 75°29'32.2"W, 2500 m, 30 Sep 2002, ♀, fruits, *David & Idarraga 291* (HUA); municipio de Caldas, vereda La Corrala, Finca “La Zarza”, Camino La Zarza – Alto del Gallinazo, ca. 2440 m, 10 Nov 1987, ♂, old flowers, *Albert de Escobar et al. 8022* (HUA); municipio de Urrao, Páramo de Frontino, 6°29'N, 76°7'W, 2890–2250 m, 16 May 1987, ♂, old flowers, *Estrada et al. 108* (HUA); municipio de Medellín, corregimiento de Santa Elena, vereda El Llano y Perico, cabeceras y recorrido Q. San Pedro, 2550 m, 8 May 1996, ♀, fruits, *Giraldo et al. 785* (HUA); **Caldas**: Vereda la Corrala, Finca La Zarza, al lado del camino entre la cascada y el Alto del Gallinazo, 2550 m, 27 Apr 1987, floral buds, *Albert de Escobar 7576* (HUA, UC); **Chocó**: municipio San José del Palmar, hacia el galápago, 1380 m, 11 Nov 1985, ♀, fruits, *Lozano et al. 4920* (COL); **Cundinamarca**: San Francisco, hacienda “La Laja”, 2880 m, 30 May 2004, ♂, flowers, *Parra et al. 465* (COL); **Huila**: municipio de la Argentina, Quebrada del pueblo, 1850 m, 25 Sep 1984, ♀, fruits, *Lozano et al. 3981* (COL); **Nariño**: Mpio. de Ricaurte, Reserva Indígena Gualcalá, Santa Fé, camino al Río Gualcalá, 1°18'N, 77°54'W, 1100–1200 m, 18 Dec 1995, inflorescence buds, floral buds, *Ramírez & González 9135* (QCA). **Quindío**: Parque Nacional de Cocora, Quebrada Santa Lucía, 3000 m, 08 May 1990, ♀, flowers, *Franco 3095* (NY); **Risaralda**: municipio de Pereira, corregimiento La Florida, SFF Otún Quimbaya, 4°44'17"N, 75°34'01"W, 1900 m, 22–28 Feb 2004, floral buds, *Alzate et al. 1962* (F); municipio de Pereira, orilla del camino entre Ceylan y el Cedral, 2150 m, 4 Dec 1989, ♀, fruits, *Franco et al. 2929* (COL); Mpio. Pereira, Parque Nat. Reg. UCUMARI, 2200–2450 m, ♀, fruits, *González 1635* (COL); municipio de Pereira, Parque Regional Ucumari, vereda la Pastora, camino de el Ceilán a El Cedral, borde de la quebrada Miraflores, 2340 m, 30 Jul 1989, floral buds, *Galeano 222* (COL); municipio de Apía, vereda “La Cumbre”, 2285 m, 24 Feb 1983, ♀, fruits, *Torres et al. 2192* (COL); **Tolima**: Quindio, El Roble, old inflorescence axis, *Triana 358* (US); same data as preceding, ♂, flowers, *Triana 359* (US); same data as preceding, ♂, flowers, Triana 2805 (US); **Valle del Cauca**: San Antonio, about 25 km W of Cali, 1800 m, 25 Jan 1988, ♂, flowers, *van der Werff & Giraldo 9746* (MEXU, MO); same data as preceding, *van der Werff & J. Giraldo 9747* (CR, MO); Cordillera Occidental, vertiente occidental, Hoya del Río Digua, lado izquierdo, Piedra de Moler, 900–1180 m, 19–28 Aug 1943, ♀, fruits, *Cuatrecasas 14974* (F); Cali, forests of West Cordillera above Cali, 2000–2200 m, June 1900, ♀, young fruits, *B.T. 623* (NY); Finca Zingara, km 18 de la carretera Calia–Buenaventura, km 4 vía a Dapa, corregimiento de la Elvira, cordillera Occidental, 1900 m, 23 Jan 1994, ♀, flowers, *Giraldo-Gensini & Agredo 130* (MO); same data as preceding, 26 Feb 1995, ♀, fruits, *Giraldo-Gensini & Agredo 611* (MO); same data as preceding, 25 Aug 1996, ♀, fruits, *Giraldo-Gensini & Corrales 765* (MO); Cuenca del Río Cali, cercanías de Peñas Blancas, 10 Jan 1963, sterile, *López Figueiras 8139* (US); Cordillera Occidental, vertiente oriental, Hoya del río Cali, río Pichindé, entre los Cárpatos y El Olivo, 2920–2025 m, 5 Aug 1946, inflorescence buds, *Cuatrecasas 21935* (F, P, US); Cordillera Occidental, Los Farallones, extremo N, vertiente oriental, Almorzadero, bosques, 2980 m, 13 Oct 1944, ♂, flowers, *Cuatrecasas 18113* (F, US); Yumbo, Finca La Samaria, NE of Darien, near Lago Calima (reservoir), 1700–1750 m, 14 Feb 1984, ♀, flowers, *Juncosa 2181* (MO); Peñas Blancas, los Cárpatos, 1600–1700 m, 9 Sep 1988, inflorescence buds, *Franco et al. 2509* (COL, MO); Bosque de San Antonio, W of Cali, near television tower, 2250 m, 15 Jul 1984, ♀, fruits, *Gentry 48079 et al.* (MO); Buga, Carretera a El Retiro, a 1 km de carretera Buga–El Placer (desvío a Retiro está a 12 km antes de El Placer), Cordillera Central, vertiente occidental, al lado de carretera, 2300 m, 15 Sep 1991, ♂, flowers, *Silverstone-Sopkin & Giraldo-Gensini 6400* (MO); Restrepo, Río Calima, Cusumbo, 680 m, 21 Feb 1989, ♀, fruits, *Devia & Prado 2581* (US); El Silencio, Hacienda Himalaya, Cordillera Occidental W of Yumbo, transect 1, 1860 m, 2 Jan 1989, ♂, flowers, *Gentry et al. 65414* (MO); Argelia, Vereda Las Brisas, 2050–2200 m, 21 Jan 1983, ♀, fruits, *Díaz P. 3854* (COL, MO); Manizales, vereda La Esperanza, Reserva Torre Cuatro, cerca de la quebrada La Siberia, 5°1'41"N, 75°23'10"W, 2650–2750 m, 28 Mar 1999, ♂, old flowers, *Alvear et al. 314* (COL); Manizales, 1851–1857, ♂, old flowers, *Triana s.n.* (K); Neira, Cementos Caldas, 5°9'45"N, 75°29'43.1"W, 2203 m, 29 Jul 2004, ♀, fruits, *Mancera 553* (COL); Manizales, Reserva Río Blanco, 2600 m, 28 Feb 2003, sterile, *Sanin 39* (HUA); Manizales, Río Blanco, El Paso nivel, 2500 m, 10 Jul 2003, sterile, *Sanin 958* (HUA). Cauca, Parque Nacional Munchique, El Tambo, vereda La Romelia, la Gallera, 1950 m, 25 Jul 1993, ♀, fruits, *Velayos et al. 6891* (COL); El Tambo, Parque Nacional Munchique, vereda La Romelia, la Gallera, 2385 m, 26 Jul 1993, ♂, old flowers, *Velayos et al. 6917* (COL). With three localities: Quindio: Mariquita, Roble, 2080 m; Antioquia, Manizales, 2140 m: Buenaventura, Cordillera Occidental, 2190 m, Jul 1833, ♂, old flowers, *Triana 4668* (COL); same data as preceding, floral buds, *Triana 1866* (photo in F of a specimen in G).

**Ecuador. Carchi**: prominent hillcrest directly N of Lita, on N side of Río Mira and just to E of Río Baboso, on steep W-facing slope, 760 m, 7 Aug 1994, sterile, *Boyle 3476* (MO); along Chical–Pailon–San Marcos trail, 01°06'N, 78°18'W, 600–1200 m, 18–19 Nov 1983, ♂, flowers, *Kvist et al. 48670* (AAU, QCA, QCNE); El Pailon, ca. 45 km above Maldonado along a footpath to Tobar Donoso, 0°49'S, 78°9'W, 800 m, 1 Dec 1979, ♀, fruits, *Madison & Besse 7226* (QCA); **Esmeraldas**: Quininde, Bilsa Biological Station, Montañas de Mache, 35 km W of Quininde, 5 km W of Santa Isabel, slope between banana plantation and stream SE of Station, 400–600 m, 9 Dec 1994, ♀, fruits, *Bass & Pitman 320* (MEXU, MO, QCNE); same data as preceding, 31 Dec. 1994, ♀, flowers, *Pitman & Marsh 1133* (MO, QCNE); San Lorenzo, Alto Tambo, Frente Finca del Sr. Lalama, a 1.5 km del sector de El Cristal, 650 m, 13 May 1992, ♂, flowers, *Quelal & Luteyn 489* (MO, QCNE); **Imbabura**: Cordillera Occidental, La Union, lower Intag Valley, 4600 ft, 20 Sep 1944, ♀, flowers, *Drew E-684* (NY, US); **Los Ríos**: Quevedo, Centinela–La Pirámide, vía Santa Domingo de los Colorados–Quevedo entrando por Patricia Pilar km 41, 650 m, 25 Feb 1992, ♂, flowers, *Quelal & Tipaz 347* (MO, QCNE); Collections from path following ridge line at El Centinela at crest of Montañas de Ila on road from Patricia Pilar to 24 de Mayo at km 12, Patricia Pilar is at Km 45 on road from Sto. Domingo to Quevedo, 600 m, 4 Feb 1983, ♀, fruits, *Dodson 13654* (MO, QCNE); same data as preceding, 6 Apr 1980, inflorescence bud, *Dodson s.n.* (MO); **Napo**: Quijos, along road 1 km SE of Cosanga, 00°35'S, 77°52'W, 2000 m, 31 Jul 1990, floral buds, *Webster & Richerson 28512* (QCNE); El Chaco, San Juan Chico, camino hacia el Río Oyacachi, 00°17'S, 77°52'W, 2000 m, ♀, fruits, *Alvarez 97 et al.* (QCNE); Baeza–Sardinas Alto, col. en borde de carretero, al NO de Sardinas Alto, a 1.5 km, 00°22'S, 77°52'W, 1780 m, 15 Dec 1993, inflorescence buds, *Freire-Fierro & Yánez 2693* (QCA, QCNE); hills above R. San Juan, at confluence with R. Oyacachi, ca 10 km W of El Chaco, 00°17'S, 77°51'W, 1800–2000 m, ♀, fruits, *Ståhl et al. 2354* (QCA); Napo–Pastaza, Mera, 26 Mar 1940, ♀, fruits, *Lugo 126* (C, MO); **Pichincha**: along road and trail from Maquipucuna Lodge to Ecolodge Santa Lucia, 2 km N of Maquipucuna entrance, 1400 m, 15 Mar 2006, ♀, fruits, *Croat 95938* (MO); Maquipucuna, 5 km E of Nanegal, Transect # 5, 1550 m, 10 Feb 1991, ♀, flowers, *Gentry & Valencia 73174* (AAU, MO, QCNE); Quito, Nanegal, Bosque protector Maquipucuna, 5 km airline SE of Nanegal, disturbed rainforest above Río Tulambi, 00°07.5'N, 78°38.5'W, 1500 m, 31 Aug 1993, ♂, old flowers, Webster et al. 30003 (QCNE); Reserva Biológica Maquipucuna, Nanegal, colectada en primer lindero, camino del Inca, 1600 m, 9 Jan 1992, ♂, flowers, *Tipaz et al. 593* (AAU, MO, QCNE); Maquipucuna Tropical Reserve, western slopes of Andes, northern boundary of reserve, 10 km north of Nanegalito, 1200 m, 2 Dec 1988, inflorescences, floral buds, *Neill et al. 8643* (MO, QCNE); Reserva Biológica Maquipucuna, 1300 m, 31 Jan 1991, ♀, flowers, *Alvarado 406* (COL, MO, QCNE); along road and trail from Maquipucuna Lodge to Ecolodge Santa Lucia, 2 km N of Maquipucuna entrance, 00°07'19"N, 78°37'06"W, 1400 m, 15 Mar 2006, ♀, fruits, *Croat et al. 95939* (MO, QCNE); Mindo, on western slopes of Andes, 1200 m, 16 Apr 1994, ♂, flowers, *Neill & Asanza 10349* (MO, QCNE); 11 km W of Tandapi, trail along Chictoa River, tributary of Río Pilatón, 1350–1550 m, 26 Oct 1974, ♀, fruits, *Gentry et al. 12073* (MO, QCA); **Tungurahua**: Baños, 2.8 airline km SSW of Río Negro, near El Coral, 1481 m, 1°25'59.5"S, 78°12'1.5"W, 13 Jun 2012, sterile, *Granados Mendoza et al. 2012-38* (GENT, HOXA, IEB, MEXU, QCNE); ca. 2 km E of Río Verde on touristic path before tunnel on road to Baños–Mera, 1629 m, 1°23'41.5"S, 78°16'53.2"W, 16 Jun 2012, sterile, *Granados Mendoza et al. 2012-44* (GENT, HOXA, IEB, MEXU, QCNE); Zamora-Chinchipe, Zamora, Jamboe Bajo, Eastern border of Podocarpus National Park, 1100 m, 5 Nov 1996, sterile, *Clark 3283* (MO); Westkordillere, 1000 m, 28 Sep 1934, sterile, *Heinrichs 741* (NY).

**Peru. Amazonas**: Bagua, Aramango, 8.86 km SE of Aramango, 1.23 km SW of Nueva Esperanza, 1806 m, 5°28'28.9"S, 78°23'08.4"W, 3 Jul 2011, sterile, *Samain et al. 2011-110* (GENT, IEB, MEXU, USM); Chachapoyas, Soloco, Paraje las Tinas, 5.26 km SE of Quitachi, E of road to Pampa del Tío, 2108 m, 6°21'06.45"S, 77°38'52.95"W, 22 Jun 2011, ♂, flowers, *Samain et al. 2011-064* (IEB, GENT, MEXU, USM); **Cajamarca**: San Ignacio, San José de Lourdes, localidad Estrella del Oriente, 1630 m, 2 Sep 1997, ♀, fruits, *Campos 4326 & Rodriguez* (F, MO, NY); **Huánuco**: Chinchao, 0.86 km ESE of Carpish, 330 m SE of road Huánuco–Tingo María, 2681 m, 9°42'24.3"S, 76°05'11.2"W, 17 Jul 2011, sterile, *Samain et al. 2011-185* (GENT, IEB, MEXU, USM); Chinchao, Carpish, 2626 m, 9°42'21.29"S, 76°05'14.46"W, 29 Nov 2013, ♂, inflorescence buds, flowers, *Samain et al. 2013-109* (IEB, GENT, MEXU, USM); same data as preceding, 9°42'20.41"S, 76°05'15.79"W, 2610 m, 29 Nov 2013, ♀, fruits, *Samain et al. 2013-112* (IEB, GENT, MEXU, USM); 0.85 km ESE of Carpish, 330 m SE of road Huánuco–Tingo María, 2700 m, 9°42'24.5"S, 76°05'11.6"W, 17 Jul 2011, sterile, old inflorescence axes, *Samain et al. 2011-186* (GENT, IEB, MEXU, USM); **Pasco**: Oxapampa, Valle de San Alberto, east of Oxapampa, above hydroelectric plant, at base of Cordillera Yanachaga, 1900 m, 1 Jan 1984, ♂, flowers, *Foster 7703 et al.* (F, MEXU, NY, USM); Oxapampa, Huancabamba, Sector Tunqui, Parque Nacional Yanachaga Chemillén, camino al valle del Palcazú, 10°16'24"S, 75°30'37"W, 2006 m, 15 Sep 2009, floral buds, *Castillo et al. 1006* (HOXA); Oxapampa, Huancabamba, Parque Nacional Yanachaga Chemillen, parte media de la Quebrada Muchuy Mayo, sector Tunqui, 10°17'30"S, 75°31'05"W, 1800 m, 26 Oct 2007, functionally male, inflorescence buds, young flowers, *Monteagudo et al. 15627* (HOXA); Oxapampa, sector San Alberto, 10°32'58"S, 75°21'58"W, 2353 m, 23 Dec 2007, ♀, fruits, *Rojas et al. 5052* (HOXA); **San Martín**: Moyobamba, Jepelacio, Cascada Paccha, 1.15 km SW of San Miguel, road Jepelacio–Carrizal, 6°13'19.5"S, 76°55'14.9"W, 1229 m, 28 Jun 2011, ♀, fruits, *Samain et al. 2011-092* (IEB, GENT, MEXU, USM).

#### 9. Hydrangea weberbaueri

Taxon classificationPlantaeCornaleHydrangeaceae

Engl., Nat. Pflanzenfam. (ed. 2) 18a: 207. 1930.

4FA144A1-D935-53C7-A2CF-8074F5035EF7

**Figure 15.**
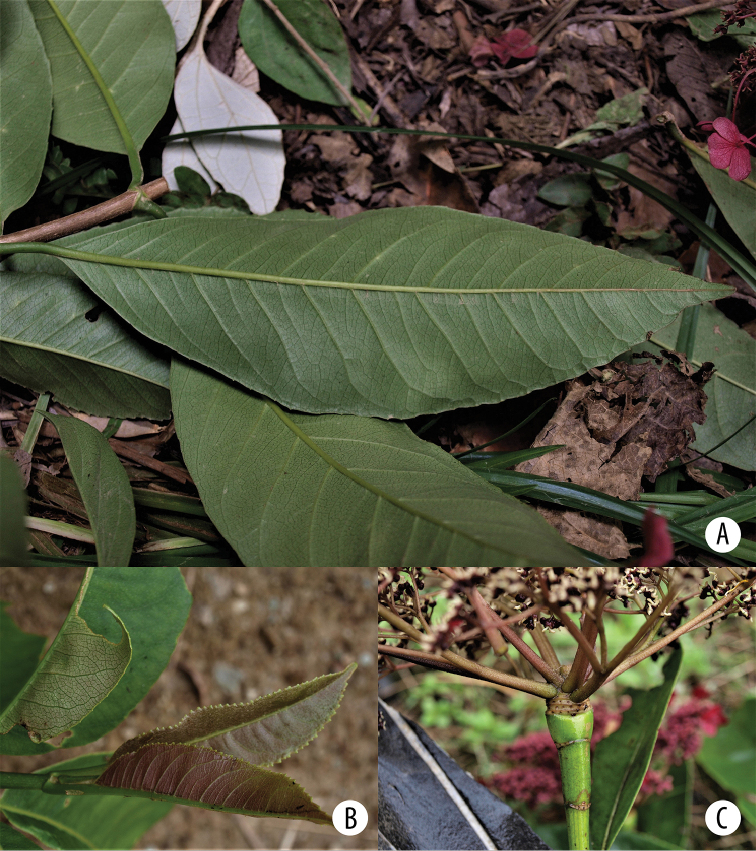
*Hydrangea weberbaueri***A** apical portion of a branch with young leaves where the characteristic leaf venation and margin can be observed **B** adaxial leaf side showing the typical leaf venation of this species **C** glabrous apex of the inflorescence axis of a functionally female inflorescence. **A** field image of collection *Granados Mendoza et al. 2012-23***B** field image of collection *Samain et al. 2011-056***C** field image of collection *Samain et al. 2011-068*.

**Figure 16.**
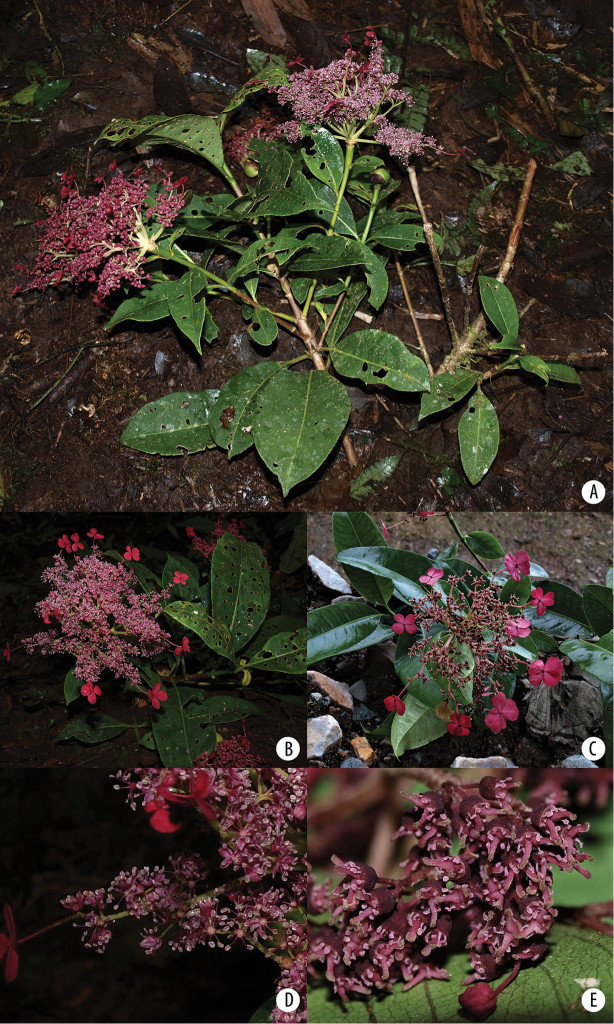
*Hydrangea weberbaueri***A** branch with inflorescences with enlarged marginal flowers **B** functionally male inflorescence with enlarged marginal flowers, and reduced flowers with large stamens **C** functionally female inflorescence with enlarged marginal flowers **D** close-up of functionally male flowers with large stamens and reduced pistils **E** close-up of functionally female flowers with reduced stamens and large pistils. **A, B** field images of collection *Granados Mendoza et al. 2012-16***C** field image of collection *Granados Mendoza et al. 2012-21***D** field image of collection *Samain et al. 2011-068*.

Figures 14 , 15 , 16

##### Type.

Peru. Amazonas: Cheto, ♀, inflorescence bud, flower buds, flowers, *A. Weberbauer 4372* (holotype: B (destroyed), F neg. 4146); lectotype: F! (fragment)). Peru. Amazonas: Chachapoyas, Soloco, Paraje Olia, 1.44 km S of Quitachi, W of road to Pampa del Tío, 6°20'2.85"S, 77°41'2.91"W, 2304 m, 22 Jun. 2011, ♀, flowers, M.S. Samain et al. 2011-062 epitype, designated here: USM!; isoepitypes: GENT!, IEB!, MEXU!).

##### Description.

Root-climbing liana of up to 20 m high, functionally dioecious; ***free-growing branches*** quadrangular, slightly fissured; ***leaves*** decussate, petiole flattened adaxially, sulcate in young leaves (Fig. 15B), terete abaxially, color dark brown to black, glabrous, leaving a semicircular to triangular scar on the branch when leaves fall, 1.0–2.6 cm long; lamina (ob)ovate to lanceolate, 13–21 cm long, 6–12 cm broad, base cuneate, sometimes asymmetric, apex rounded to acuminate, leaf margin slightly glandular-dentate, venation brochidodromous, veins 6–10 pairs, adaxial leaf side with midvein protruding, primary veins lightly marked, secondary veins barely visible, moderately pubescent with white, stellate hairs, caducous, abaxially with protruding midvein and primary veins, secondary veins marked, opaque olive green, secondary veins in between the primary veins parallel to these and forming a distinctive regular pattern with veins arching towards the apex (Fig. 15A), sometimes, primary and secondary veins starting from nearly the same position on the midvein, densely pubescent with stellate reddish hairs, especially on the veins, caducous, sometimes nearly glabrous, acarodomatia numerous, not very conspicuous as they are hidden under the pubescence, in axils of midvein and primary veins, sometimes forming a small membrane partially covering the cavity; ***inflorescence axis*** pubescent with simple white hairs to glabrous (Fig. 15C), 9–47 cm long, with 3–5 decussate leaf pairs below the inflorescence, persistent, petiole 0.5–1 cm long, lamina 2–11.5 cm long, 1.1–7.5 cm broad, adaxially glabrous, abaxially pubescent with small stellate hairs, ***apex of the inflorescence axis*** woody, cylindrical, elongated bract scars visible, thickening at the top, 6–10 mm broad, 4–7 mm high in functionally female plants, 5–7 mm broad, 4 mm high in functionally male plants, inflorescence bracts cucullate, cream-colored, coriaceous, margin membranous and glabrous to densely pubescent with yellowish stellate hairs, veins darker, parallel, bracts increasing in size, lowermost bract 2.5–3 cm large, 2.4–2.5 cm broad, ***inflorescences*** lateral, decussate, 1–3 pairs of inflorescences per flowering branch (Fig. 16A), sometimes only one inflorescence developing, flowering branch continues growing vegetatively with the same vigor and periodicity above the inflorescences as below these, inflorescence axes with basal lignified parts of inflorescences of previous years visible in well-collected specimens, allowing to observe growth and flower periodicity, these rests 53 cm apart (in between the main branch ramified and those branches also carried inflorescences that flowered at the same time as the last inflorescences, *Samain et al. 2013-130*), medulla central in the branch, disappearing in older branches, leaving a hole only, inflorescence umbellate, buds up to 4.1 cm broad and 3.6 cm high before opening, in flowering stage 8–22.5 cm diameter, 3.5–14 cm high, with 6–10 main axes in functionally male plants, 7–14 main axes in functionally female plants, partial inflorescences cymes, secondary and tertiary inflorescence axes with reddish stellate hairs, pubescence gradually increasing towards flower insertion; ***enlarged marginal flowers*** sometimes present (Fig. 16A–C), terminally placed in a cyme, sepals with marked veins, (2–)4, separate at the base, veins marked, palmate, reticulate, pistils 2, reduced, central part of the flower generally amorph, further characters not observed in detail, 1.5–3.7 cm diameter, pedicel 1–2.5 cm long; ***flower pedicel of reduced flowers*** 3–7 mm long in functionally male flowers, 3 mm long in functionally female flowers, ***receptacle*** triangular in functionally male flowers, rectangular in functionally female flowers, ***ovary*** inferior, ***calyx lobes*** 4, triangular, 0.2 mm long, ***petals*** 3–4, purple, valvate, cucullate, papyraceous, 2.2–2.5 mm long, 1.2–1.3 mm broad; ***functionally male flowers*** (Fig. 16D): hypanthium 1.1–1.8 mm diameter, stamens 8, well-developed, filaments 3 mm long, anthers 0.8–1.0 mm long, 0.6 mm broad, pistils 2, reduced, 0.5 mm long, stigmas not penicellate; ***functionally female flowers*** (Fig. 16E): hypanthium 1.0–1.8 mm diameter, stamens 8, reduced, filaments 0.1–0.4 mm long, anthers 0.1 mm long, 0.3–0.4 mm broad, pistils 2, 0.7 mm long, enlarging to 2–3 mm during fruit maturation, stigmas apically clavate and shortly penicellate; ***fruit*** a rectangular capsule, apically with a conspicuous border, ribs 4–6, pale reddish brown, 1.5 mm high, 2.5 mm broad above, 1.5 mm diameter, opening between the two pistils to release seeds, seeds not seen.

##### Distribution.

*Hydrangea weberbaueri* is known from Colombia, Ecuador and Peru (Fig. 14).

##### Habitat.

This species grows in tropical rainforest and mountain cloud forest at elevations between 974–3500 m.

##### Phenology.

This species has been collected with flowers and fruits throughout the year.

##### Vernacular names and use.

The following vernacular names are indicated on the specimen *Ellemann 75381* (AAU, LOJA, MO, QCA): bejuco matapalo (Spanish), yura huanutic caspic (Quichua). Its use as fuel wood is also indicated on the label of these specimens. This is the only species of this study of which vernacular name and use have been recorded.

##### Notes.

Not to be considered a synonym of *H. peruviana* from which it can be distinguished by the notorious regular venation pattern with primary veins and secondary veins parallel to each other, all of them arching towards the apex.

No duplicate material of the original type collection has been located in the herbarium MOL of the Universidad Nacional Agraria La Molina in Lima, Peru, nor any other herbarium where we have searched. The fragment held in F that is the obligate lectotype is fragmentary and does not have the diagnostic features of this taxon, therefore we designate an epitype here for diagnostic purposes. The epitype specimen was collected approximately 8.5 km south of the original type location, which at the time of our field work was the nearest location to Cheto with primary forest. Unfortunately, satellite images (Google Earth 2020) show that the forest where the epitype has been collected was destroyed for land use change less than five years after our visit.

A Weberbauer collection with the same number as the original type is present in the herbarium USM, but this is an entirely different species (with branched inflorescences) which does not belong to this group. The label on the USM specimen is not the original Weberbauer label, suggesting this represents a labeling error.

The leaf venation in the specimens from Colombia does slightly differ from that in the material from Ecuador and Peru, although it is still very recognizable. Therefore, we decided to include these in *H. weberbaueri*, although future studies on Colombian hydrangeas may well show it concerns a distinct and new species.

According to our molecular study, *Hydrangea weberbaueri* is not closely related to any of the species mentioned here, but it occurs in a clade which is sister to the one with *H. goudotii* and *H. trianae* (Granados Mendoza et al. unpublished results).

##### Preliminary conservation status.

Although this species has an EOO of nearly 660,000 km^2^, it is Endangered according to the IUCN categories and criteria (IUCN 2012), with an AAO of 224 km^2^, as well as an extensive reduction in both EOO and AOO resulting from severe habitat destruction and fragmentation.

##### Additional specimens examined.

**Colombia. Cundinamarca**: Quebrada la Aguadita, 2 km arriba de la carretera a Fusagasugá, 1800 m, 28 Nov 1960, ♀, fruits, *Idrobo 4186* (COL); Cordillera Oriental, municipio San Bernardo, Vereda de Venecia, margen izquierda del río Chorrera, afluente del Sumapáz, 2450 m, 21 Jul 1981, floral buds, *Jaramillo et al. 6844* (COL); Sasaima, vereda San Bernardo, quebrada La María y Río Dulce, 1800 m, sterile, Jan 1958, sterile, *García-Barriga 15757* (COL); **Santander**: municipio Suaita, corregimiento San José de Suaita, carretera a La Veterana, bosque al lado de la carretera, vereda San Imidio, 6°9'44.5"N, 73°25'09.4"W, 1800–1910 m, 6 Apr 2003, ♀, fruits, *Betancur et al. 10122* (COL, HUA).

**Ecuador. Azuay**: the eastern Cordillera, 1–8 km, north of the village of Sevilla de Oro, 8000–9000 ft, 27 Jul–12 Aug 1945, inflorescence buds, *Camp E-4464* (AAU, F, MO); Sevilla de Oro, ca 1.7 airline km E of Osorancho (El Cisne), N Sevilla de Oro, 2°45'42"S, 78°37'38.1"W, 2804 m, 8 Jun 2012, sterile, *Granados Mendoza et al. 2012-22* (GENT, HOXA, IEB, MEXU, QCNE); same data as preceding, 2°45'42.1"S, 78°37'37.4"W, 2804 m, 8 Jun 2012, sterile, *Granados Mendoza et al. 2012-23* (GENT, HOXA, IEB, MEXU, QCNE); same data as preceding, 2°45'42"S, 78°37'37.3"W, 2805 m, 8 Jun 2012, sterile, *Granados Mendoza et al. 2012-24* (GENT, HOXA, IEB, MEXU, QCNE); ca 1.8 airline km E of Osorancho (El Cisne), N Sevilla de Oro, 2°45'43.3"S, 78°37'35.9"W, 2813 m, 8 Jun 2012, inflorescence buds, *Granados Mendoza et al. 2012-25* (GENT, HOXA, IEB, MEXU, QCNE); **Loja**: Río Jipiro near Hda. Jipiro, about 7 km northeast of Loja, 03°58'S, 79°07'W, 8000 ft, 14–16 Jun 1944, ♂, flower buds, flowers, *Prieto AP-27* (AAU, NY); between Nudo de Sabanillas and Río Cachiyacu at Tambo Cachiyacu, 3000–3500 m, 17 Oct 1943, ♂, flowers, *Steyermark 54551* (F, US); Loja–Zamora, 12 km from Loja, on the finca of Dr. David Espinosa, 03°55'S, 79°09'W, 2400–2600 m, 17 Nov 1988, ♀, fruits, *Ellemann 75381* (AAU, LOJA, MO, QCA); old road Loja-Zamora, El Tiro, 2600 m, 10 Oct 2004, ♂, flowers, *Homeier 1399* (LOJA, QCNE); Loja, 3.5 Km S of Nudo de Sabanillas on road Yangana–Valladolid, 4°26'54.2"S, 79°8'51.3"W, 2798 m, 2 Jun 2012, sterile, *Granados Mendoza et al. 2012-11* (GENT, HOXA, IEB, MEXU, QCNE); **Morona Santiago**: Parque Nacional Sangay-Tinguichaca, 2°13'559632"S, 78°26'364956"W, 2870 m, 29 Aug 2011, sterile, *Duchicela et al. 759* (QCA); Morona, ca. 4.5 airline km WNW of Plan de Milagro on road Plan de Milagro–Gualaceo, 3°0'14.6"S, 78°30'29.2"W, 2158 m, 7 Jun 2012, ♀, fruits, *Granados Mendoza et al. 2012-21* (GENT, HOXA, IEB, MEXU, QCNE); **Napo**: area of the Yanayacu biological station, 5 km SW of Cosanga, 00°36'S, 77°53'W, 2100 m, 27 Sep 2005, ♀, fruits, *Homeier 1680* (QCNE); **Pichincha**: Reserva Florística – Ecológica “ Río Guajalito”, km 59 de la carretera antigua Quito – Sto. Domingo de los Colorados, a 3.5 km al NE de la carretera, estribaciones occidentales del Volcán Pichincha, 0°13'53"S, 78°48'10"W, 1800–2200 m, 9 Aug 1985, ♂, flowers, *Jaramillo & Zak 7900* (NY, QCA, US); same data as preceding, 2200 m, 25 Apr 1987, sterile, *Grijalva 317* (QCA); road from Chiriboga to Santo Domingo, 1910 m, 3 May 1985, ♂, flowers, *Stein 2680* (AAU, NY, QCNE); Cordillera Oriental, Oya Cachi, 3200 m, 26 Oct 1945, ♂, old flowers, *Acosta 11144* (F); **Sucumbios**: Gonzalo Pizarro, near km 14 of Vía a casa de Máquinas of Bosque Protector los Cedros, 0°7'21.9"S, 77°26'42.1"W, 974 m, 9 Jul 2012, sterile, *Granados Mendoza et al. 2012-103* (GENT, HOXA, IEB, MEXU, QCNE); **Tungurahua**: Cordillera el Encanto, ca. 6 airline km SSW of Río Negro, 1°27'33.4"S, 78°13'7"W, 1815 m, 13 Jun 2012, floral buds, *Granados Mendoza et al. 2012-35* (GENT, HOXA, IEB, MEXU, QCNE); same data as preceding, 1°27'34.6"S, 78°12'53.7"W, 1810 m, 13 Jun 2012, inflorescence buds, *Granados Mendoza et al. 2012-36* (GENT, HOXA, IEB, MEXU, QCNE); same data as preceding, 1°27'42.5"S, 78°12'52"W, 1806 m, 13 Jun 2012, inflorescence buds, *Granados Mendoza et al. 2012-37* (GENT, HOXA, IEB, MEXU, QCNE); **Zamora-Chinchipe**: Carretera Loja–Zamora, km 15–18, falda oriental, 3°58'S, 79°7'W, 23 Sep 1982, floral buds, *Balslev 3181* (AAU, MO, NY, QCA); Zamora, 3.7 km on foot path S Romerillos Alto, 4°14'49.9"S, 78°56'9.1"W, 1681 m, 4 Jun 2012, ♂, inflorescence buds, flowers, *Granados Mendoza et al. 2012-16* (GENT, HOXA, IEB, MEXU, QCNE); ca. 4.8 km on foot path S Romerillos Alto, 4°15'0.6"S, 78°55'40.4"W, 1801 m, 4 Jun 2012, sterile, *Granados Mendoza et al. 2012-17* (GENT, HOXA, IEB, MEXU, QCNE); same data as preceding, 4°15'0"S, 78°55'39.9"W, 1795 m, 4 Jun 2012, sterile, *Granados Mendoza et al. 2012-18* (GENT, HOXA, IEB, MEXU, QCNE); same data as preceding, 4°15'0.2"S, 78°55'39.4"W, 1796 m, 4 Jun 2012, ♂, flowers, *Granados Mendoza et al. 2012-19* (GENT, HOXA, IEB, MEXU, QCNE); El Pangui, ca. 5.7 km SSE of Tundayme on dirt road to military base El Condor, 3°36'19.6"S, 78°28'21.1"W, 1092 m, 7 Jun 2012, sterile, dry inflorescences, *Granados Mendoza et al. 2012-20* (GENT, HOXA, IEB, MEXU, QCNE).

**Peru. Amazonas**: Bongará, Jumbilla, Bosque de Protección Alto Mayo, surroundings of Chichilac, 4.79 km NNE of Jumbilla, 5°51'51.33"S, 77°47'16.7"W, 2462 m, 10 Dec 2013, ♀, old fruits, *Samain et al. 2013-130* (GENT, IEB, MEXU, USM); same data as preceding, 4.65 km NNE of Jumbilla, 5°51'56.7"S, 77°46'55.6"W, 2649 m, 30 Jun 2011, ♂, flowers, *Samain et al. 2011-103* (GENT, IEB, MEXU, USM); same data as preceding, 4.57 km NNE of Jumbilla, 5°51'51.9"S, 77°47'16.7"W, 2397 m, 30 Jun 2011, ♀, flower buds, flowers, *Samain et al. 2011-097* (GENT, IEB, MEXU, USM); Bosque de Protección Alto Mayo, surroundings of Chichilac, 4.78 km NNE of Jumbilla, 5°51'52.51"S, 77°47'0.86"W, 2570 m, 10 Dec 2013, ♀, fruits, *Samain et al. 2013-133* (GENT, IEB, MEXU, USM); Bongará, Yambrasbamba, carretera Chiclayo–Tarapoto, km 370.2, antes de Puente Nieva, 5°41'6.1"S, 77°46'56.5"W, 2031 m, 9 Dec 2013, ♂, flower buds, flowers, *Samain et al. 2013-127* (GENT, IEB, MEXU, USM); margen del Río Nieva, km 381 de la Carretera Marginal, 5°41'S, 77°47'W, 1070 m, 11 Jul 1999, ♀, flower developing into fruits, *Sánchez Vega 10031* (F); Florida, 4–8 km E of Pomacocha on road to Rioja, 5°45'S, 77°53'W, 2100–2200 m, 9 Feb 1984, ♂, flowers, *Gentry 45191* (AMAZ, CAS, COL, MEXU, MO); Chachapoyas, Leymebamba, Hacienda San Cristobal del Yeso, 3.08 km NW of Leymebamba, 6°41'34.9"S, 77°49'36.3"W, 2766 m, 20 Jun 2011, ♂, flowers, *Samain et al. 2011-046* (GENT, IEB, MEXU, USM); same data as preceding, Hacienda San Cristobal del Yeso, 2.7 km NNW of Leymebamba, 6°41'39.33"S, 77°49'29.23"W, 2738 m, 8 Dec 2013, ♀, fruits, *Samain et al. 2013-124* (GENT, IEB, MEXU, USM); alrededor de la Laguna de Los Cóndores, parte sur, 6°51.20'S, 77°40.96'W, 2500–2700 m, 16 Aug 1998, ♀, fruits, *Quipuscoa 1218* (F, NY, QCNE); San Francisco de Daguas, 2.4 km NNE of Pipus, Chachapoyas–Molinopampa road, km 30, 6°12'32.3"S, 77°43'22.8"W, 2110 m, 21 Jun 2011, ♀, fruits, *Samain et al. 2011-056* (GENT, IEB, MEXU, USM); Pampa del Tio, camino entre Quitachi y (las) Tinas, 2000 m, 5 Jul 1991, ♂, flowers, *Díaz 4609* (USM); **Cajamarca**: Cutervo, San Andrés de Cutervo, 3.9 km SE of San Andres Cutervo, road Cutervo–Socota–Chiple, 6°16'05.6"S, 78°41'35.4"W, 2513 m, 8 Jul 2011, inflorescence buds, *Samain et al. 2011-141* (GENT, IEB, MEXU, USM); same data as preceding, 3.92 km SE of San Andrés de Cutervo, road Cutervo–Socota–Chiple, 6°16'05.5"S, 78°41'34.5"W, 2518 m, 8 Jul 2011, ♀, inflorescence buds, *Samain et al. 2011-143* (GENT, IEB, MEXU, USM); La Pucarilla, entre Socota y San Andrés, 2500 m, 3 Nov 1991, ♂, flowers, *Sánchez 5947* (F, MO, UC); Bosque Cutervo, Parque Nacional de Cutervo, NW corner of Cordillera Tarros, Chorro Blanco sector, ca. 10 km WNW of San Andres de Cutervo, 6°12'S, 78°46'W, 2650 m, 4 Nov 1990, ♂, inflorescence buds, flowers, *Dillon 6152* (F, UC); Cutervo National Park, Chorro Blanco area, 15 km N of San Andres de Cutervo, 6°10'S, 78°40'W, 2400–2650 m, 13 Sep 1991, ♀, flower buds, flowers, *Gentry 74745* (F, USM); Parque Nacional Cutervo, arriba del Saucedal pasando por Chorro Blanco, 2250 m, 3 Aug 1988, ♂, inflorescence buds, flower buds, flowers, *Díaz 2943* (USM); Cutervo National Park, 12 km NE of San Andres Cutervo, transect 5, 6°10'S, 78°40'W, 2240 m, 11 Sep 1991, sterile, *Gentry 74670* (MO, USM); Madre Mia, entre el Suro y la Flor al NO del Parque, 2400 m, 25 Jun 1992, ♂, inflorescence buds, flower buds, flowers, *Sánchez Vega 6329* (F); Bosque Cutervo, Parque Nacional de Cutervo, NW corner of Cordillera Tarros, Chorro Blanco sector, ca. 10 km WNW of San Andres de Cutervo, 6°12'S, 78°46'W, 2600 m, 5 Nov 1990, ♂, flowers, *Dillon 6184* (F); Hualgayoc, 2 km. above Palmito. Hacienda Taulis, between the Casa Hacienda and Palmito, 2575 m, 31 Aug 1964, ♂, flowers, *Hutchinson 6395* (GH, F, US); San Ignacio, Huarango, poblado Selva Andina, 05°03'37"S, 078°45'13"W, 1798 m, 18 Apr 2007, ♀, fruits, *Perea & Mateo 2929* (MO, QCNE); San Ignacio, 12.83 km SW of San Ignacio, 3.05 km SSW of Alto Ihuamaca, 5°12'52.8"S, 79°05'47.9"W, 2129 m, 6 Jul 2011, ♀, fruits, *Samain et al. 2011-127* (GENT, IEB, MEXU, USM); Nuevo Mundo–Pisaguas, 5°10'S, 68°32'W (the correct coordinates are 78°32'W), 1550 m, ♀, fruits, 13 Nov 1997, *Campos 4632* (F, MEXU, MO, NY, USM); **Pasco**: Oxapampa, Parque Nacional Yanachaga Chemillén, parte alta del Refugio el Cedro, 10°32'S, 75°21'W, 2450–2680 m, 20 Mar 2003, inflorescence buds, *Monteagudo 4762* (F, HOXA, USM); Oxapampa, sector Chacos parte media, 10°38'42.9"S, 75°17'30"W, 2740 m, 4 Nov 2004, floral buds, *Monteagudo et al. 7534* (HOXA); Oxapampa, camino a Paucartambo (a la altura de Mesapata), < 1800 m, 21 Mar 2005, ♀, flower buds, fruits, *Arias et al. 261* (HOXA); Oxapampa, Huancabamba, camino hacia la Laguna San Daniel, Granapazú-Sector San Daniel, Parque Nacional Yanachaga-Chemillén, 10°26'15.6"S, 75°27'9.9"W, 2200 m, 12 Sep 2005, sterile, *Ortiz et al. 950* (HOXA); Oxapampa, Huancabamba, Parque Nacional Yanachaga-Chemillen, sector San Daniel, 10°26'35S, 75°26'16W, 2200–2500 m, 6 Mar 2006, ♂, flowers, *Vásquez et al.* 30989 (HOXA); Oxapampa, Huancabamba, sector Quebrada Yanachaga (paralela a la quebrada), 10°24'08"S, 75°29'18"W, 2097 m, 14 Sep 2004, sterile, *Perea & Mateo 1682* (HOXA); Oxapampa, El Abra, camino Oxapampa–Villa Rica, 10°40'30"S, 75°18'21"W, 2365 m, 24 Apr 2004, ♂, flowers, *Rojas et al. 2242* (HOXA); **San Martín**: Mariscal Cáceres, Huicungo, Río Abiseo National Park, 7°S, 77°W, 2700 m, 22 Jul 1985, ♀, flowers, *Young 1230* (F, NY); near La playa base camp, Río Abiseo National Park, 7°27'N, 77°2'W, 2650 m, 30 Aug 1985, ♂, inflorescence buds, flowers, *Young 1512* (MO); Río Abiseo National Park, ridge top on hill E of that with Gran Pajaten ruins, 2550 m, 14 Aug 1986, ♀, fruits, *Young 4141* (USM); trail between La Playa camp and Papayas camp, Rio Abiseo National Park, 7°S, 77°W, 2650–2750 m, 25 Jul 1987, ♀, flower buds, flowers, *Young 5014* (F, USM); Pardo Miguel, Caserío Jorge Chávez, km 398 de Carretera Marginal, 5°40'S, 77°43'W, 1400 m, 1 Jul 1999, ♀, fruits, *Sánchez Vega 9966* (F); **Rioja**: Pardo Miguel, 7.90 km E of El Progreso, Chiclayo–Tarapoto road, km 369.7, 5°41'18.8"S, 77°47'12.0"W, 2063 m, 23 Jun 2011, ♀, flower buds, flowers, *Samain et al. 2011-067* (GENT, IEB, MEXU, USM); same data as preceding, 7.94 km E of El Progreso, Chiclayo–Tarapoto road, km 369.7, 5°41'16.9"S, 77°47'11.9"W, 2132 m, 23 Jun 2011, ♀, inflorescence buds, flower buds, flowers, fruits, *Samain et al. 2011-068* (GENT, IEB, MEXU, USM); carretera Chiclayo–Tarapoto, km 377.5, 5°35'59.8"S, 77°45'18.7"W, 1867 m, 9 Dec 2013, ♀, fruits, *Samain et al. 2013-129* (GENT, IEB, MEXU, USM); Buenos Aires, along road Pedro Ruiz–Rioja, 5°42'9"S, 77°53'6"W, 2000 m, 21 Mar 1998, ♀, inflorescence buds, flower buds, flowers, *van der Werff 15329* (MEXU, USM).

## Discussion

### Unraveling taxonomic chaos in Central and South American *Hydrangeas*

The review of this morphological species complex surrounding *Hydrangea peruviana* and *H. oerstedii* of mostly red to purple flowered Central and South American climbing Hydrangeas attempts to unravel the taxonomic chaos in the group, based on the most complete sampling to date of herbarium specimens, as well as our own field collections and observations throughout their distribution area. Very importantly, type material was studied in all cases, generally the specimens themselves, rarely high-resolution scans on JSTOR Global Plants or shared by the curators of the respective herbaria. Many taxa had been synonymized, as important characters were, following their first descriptions in the 19^th^ and early 20^th^ centuries (especially by Briquet (1919), the most recent ones described in 1930), not considered of taxonomic value to distinguish species, except for the excellent treatment of the genus in the Flora of Peru (Macbride 1938). Additionally, in this particular group of Hydrangeas, we hypothesize that the authors who published on this group following Macbride (1938) did not have access to the type specimens, hence some misconceptions were merely copied from earlier sources, instead of critically reviewed with the support of the original material. Most of these nine species have been described based on the type specimen only (hence, the importance of studying type material) or on very few collections altogether, but after careful comparison of all currently known collections with type material, we can be certain that all abovementioned taxa deserve recognition as separate species, based on an extensive number of morphological characters, combined with distribution patterns, phenological differences and ecological preferences. Moreover, most of these species are also supported by our ongoing molecular studies (Granados Mendoza 2013; Granados Mendoza et al. unpublished results).

This study, as are those of other “species complexes” we are currently studying, e.g., *Hydrangea asterolasia* Diels, *Hydrangea preslii* Briq. and *Hydrangea* species with branched inflorescences, is extremely complicated, and the result of a decade of field and herbarium work, matching and re-matching specimens with type material and descriptions, comparisons with studies by McClintock (1957), Freire-Fierro (2004) and Christenhusz (2009), and adding many additional observations. These not only concern those related to functional dioecism, but also to plant branching, pubescence, leaf venation, presence or absence of acarodomatia, inflorescence architecture, and morphology of marginal flowers, amongst other characters.

As a result of our study, the number of accepted species in *Cornidia* currently totals 26. A complete key for morphological identification of all Neotropical species can currently not be provided as the species we have not yet treated (with the exception of this study, and those by Samain et al. 2014, 2019) are not yet well-defined at continental scale.

### Classification of species in Hydrangea section Cornidia

In his otherwise excellent revision of the then known species of *Cornidia*, including the description of 11 new species in the section, all unusually elaborate for that time, Briquet (1919) classified all species in two subsections, each with their respective series: subsection Monosegia Briq, with series *Speciosae* Briq. and Aphananthae Briq., and subsection Polysegia Briq. subdivided in series *Synstyleae* Briq. and Chorystyleae Briq. Subsection Monosegia, according to Briquet (1919), is characterized by a single pseudo-umbellate cyme, and its series by the presence or absence of “sterile flowers”, respectively (these flowers are not sterile, but have enlarged sepals; hence, we prefer the term “enlarged marginal flowers”, see Samain et al. 2014). Subsection Polysegia, again according to Briquet (1919), can be recognized by the thyrsoid inflorescence consisting of several pseudo-umbellate cymes and its series amongst others by short stamens and pubescent leaves, and long stamens and glabrous leaves, respectively. Although the morphological classification in subsections at first sight may seem valid, several specimens of different species we have observed both in the field and in herbarium collections show both inflorescence types (single clusters in the axil of a leaf vs. several clusters on a branched inflorescence), indicating that these subsections are not natural, which is also reflected by our ongoing molecular work (Granados Mendoza 2013; Granados Mendoza et al. unpublished results). With respect to the series of subsection Monosegia, we have observed that, although the enlarged marginal flowers generally characterize specific species, they may also be absent in some individuals of those species (although this is not the case in the species studied here), or they may be present, reduced in size or parts, or not reduced, in species that generally do not possess them. With respect to the series of the subsection Polysegia, the first character is the stamen length, but this seems very artificial, as this distinguishes both sexes within a dioecious species: stamens in male individuals have long filaments, whereas these are short in female individuals. Hence, it may be hypothesized that this classification seemed workable based on the relatively few specimens Briquet (1919) had access to at the time, but current morphological observations of an extensive number of specimens, as well as molecular data of a representative number of these, show that this is not the case. Based on our currently available data, we do not have sufficient elements to subdivide Hydrangea section Cornidia into subsections (Granados Mendoza et al. unpublished results).

### Growth form controversies

A surprisingly high number of herbarium labels of the specimens studied here, as well as some references (e.g., Christenhusz 2009), mention growth forms such as hemiepiphytic shrubs, hemiepiphytic lianas, hemiepiphytic trees, epiphytes, shrubs, trees, climbing shrubs, parasitic shrubs or trees, etc. To our knowledge, and according to our observations in the field, all representatives of *Cornidia* are root-climbing lianas. When the base of their stem or their host tree is damaged or cut, they will die very soon because of lack of water and nutrients (personal observations), and lack of support, respectively. This stresses the importance of precise field observations for a correct interpretation of plant habits.

### Distribution patterns

Of the nine species mentioned above, five are relatively widespread, whereas four have a relatively to very restricted distribution. On the one hand, the taxonomic and conservation statuses of two endemic Colombian species, *H. durifolia* and *H. schlimii*, are not completely clear in the absence of more and/or recent collections. A third species, *H. caucana*, endemic to Colombia as well, also does not have recent collections, but the existing ones do allow to understand its distribution pattern in the western cordillera. The fourth species with a restricted distribution is *H. peruviana* from Ecuador. On the other hand, *H. oerstedii* is the most widespread species studied here, known from more than 150 collections from Costa Rica and Panama. Despite the fact that this species grows in montane cloud forest, which is seriously being reduced throughout is whole distribution area (see Bruijnzeel et al. 2010), it seems to be very resilient against disturbance, as we have often observed it along roads and near villages, since it basically only needs its host tree to persist. As it is one of the few *Hydrangea* species that bends downwards with age and because of its conspicuous inflorescences, it may even be favored by local people. *Hydrangea panamensis*, which also is restricted to Costa Rica and Panama, is much less common than *H. oerstedii* (known from 17 collections only) and grows in tropical rainforest. *Hydrangea goudotii* and *H. trianae* are yet two other species described from Colombia. The present study confirms that both extend southwards into Ecuador, and the latter one also into Central Peru, being the most widespread species of this group in South America (known from 109 collections). Finally, *Hydrangea weberbaueri*, described from northern Peru, is shown here to have a continuous distribution from northern Ecuador to northern Peru, with a few collections from central Peru and from northeastern Colombia. The latter, as mentioned above, differ slightly from the southern ones, but can still be referred to this species based on the peculiar leaf vein patterns.

The overall distribution area of the species studied here is from northern Costa Rica to central Peru, which is the area with the highest diversity of Neotropical Hydrangeas, apart from Mexico (Samain et al. 2014, 2019). The few specimens of the countries north of Costa Rica and south of Mexico (Guatemala, Honduras, El Salvador and Nicaragua) belong to the widespread *Hydrangea albostellata* Samain, Najarro & Martínez (Samain et al. 2014) or remain unidentified because of a lack of fertile material, but definitely do not show affinity with the group studied here. Similarly, the few fertile specimens from Venezuela do have white flowers and belong to a group we will be treating in an upcoming study. The few specimens collected southwards of the Selva Central in Peru are generally also morphologically distinct. From both this study and ongoing morphological work in *Cornidia*, it becomes clear that especially Colombia needs to be further explored with respect to *Hydrangea*, as the record in this country seems very fragmentary and several species are known from very few collections or their type locality only.

### Impact of taxonomic studies on conservation actions

Although a few populations of most of the species studied here do occur in protected areas, their long-term conservation is seriously compromised because of the significant threats from which their habitats suffer throughout their distribution area, related to fragmentation, land use change and climate change. Moreover, it is likely their dioecism makes these species even more prone to local extinction because of habitat destruction and fragmentation, as female and male individuals generally do not occur near each other, and their functional biology is not well-understood to date. During our field work, especially in Peru, where Hydrangeas often occur on private properties, in contrast with e.g. Costa Rica and Panama, where many collections were made in areas protected at national level, we have tried to sensitize the land-owners about the uniqueness of this group as a whole. Derived of the current contribution, we will also be able to do so at species level, which is especially important for the threatened taxa.

Finally, it is difficult to believe – though not surprising taking into account our recent discoveries of seven new species in Mexico (Samain et al. 2014, 2019) – that one species, of which we now know it is extremely rare (*H. peruviana*), was considered to encompass eight other taxa which can be recognized by distinctive and species-constant characters, either at the varietal level (*H. oerstedii*), or as synonyms of the typical variety or of var. oerstedii. Moreover, if we look at global biodiversity databases such as GBIF, the situation is even worse, as the majority of the known specimens of the white-flowered *H. seemannii*, endemic to northwestern Mexico, and several species of Central America, northern South America, Peru and Bolivia are also identified as *H. peruviana*, whereas this species is endemic to Ecuador. This latter situation calls for a more critical use of such database information, as e.g., when preparing a distribution map of *H. peruviana* with GBIF data in GeoCAT (Bachmann et al. 2011) for the purpose of an IUCN Red List assessment, one might come up with an Extent of Occurrence (EOO) of nearly 12 million km^2^ based on 599 occurrence points reaching from Canada to Bolivia, whereas the correct EOO is 13,515.020 km^2^ only, based on 7 verified occurrence points in Ecuador. Even the combined EOO of all species of this study does not total 2 million km^2^. As a consequence, the present study exemplifies the urgent need for more interest for profound taxonomic studies in plants in general and funding to carry these out; though intrinsic costs are not high, these studies are very time-consuming, as in many plant families we do not dispose of well-circumscribed units for conservation to mitigate the currently occurring unprecedented loss of biodiversity.

## Supplementary Material

XML Treatment for Hydrangea caucana

XML Treatment for Hydrangea durifolia

XML Treatment for Hydrangea goudotii

XML Treatment for Hydrangea oerstedii

XML Treatment for Hydrangea panamensis

XML Treatment for Hydrangea peruviana

XML Treatment for Hydrangea schlimii

XML Treatment for Hydrangea trianae

XML Treatment for Hydrangea weberbaueri
